# Supplementary material for: Evaluation of pharmacokinetics, safety, and efficacy of [211At] meta-astatobenzylguanidine ([211At] MABG) in patients with pheochromocytoma or paraganglioma (PPGL): A study protocol
Source: PLoS One. 2024 May 28;19(5):e0303623. doi: 10.1371/journal.pone.0303623 (PMC11132457; doi:10.1371/journal.pone.0303623)
Supplement: S1 File — (PDF) [file pone.0303623.s002.pdf]

^{211}At -MABG の褐色細胞腫/パラガングリオーマ患者における

薬物動態、安全性および有効性の評価

（第Ⅰ相試験）

MABG-01

治験実施計画書

試験の相：PhaseⅠ

福島県立医科大学

第 1.3.0 版　2022年 9 月 9 日

略号一覧

略号	略してない表現	説明
ADR	adverse drug reaction	副作用
AE	adverse event	有害事象
ALP	alkaline phosphatase	アルカリフォスファターゼ
ALT（GPT）	alanine transaminase	アラニンアミノトランスフェラーゼ
AST（GOT）	aspartate aminotransferase	アスパラギン酸アミノトランスフェラーゼ
AUC	area under the curve	血中濃度曲線下面積
BNP	brain natriuretic peptide	脳性ナトリウム利尿ペプチド
BUN	blood urea nitrogen	血液尿素窒素
CFR	code of federal regulations	連邦規則集
Cl	chloride	クロール
CR	complete response	完全奏効
CRP	c-reactive protein	C 反応性タンパク
CT	computed tomography	コンピューター断層撮影
CTCAE	common terminology criteria for adverse events	有害事象共通用語基準
CVD	cyclophosphamide, vincristine, dacarbazine	シクロホスファミド、ビンクリスチン、ダカ ルバジン併用療法
DLT	dose limiting toxicity	用量制限毒性
DNA	deoxyribonucleic acid	デオキシリボ核酸
ECOG	Eastern Cooperative Oncology Group	米国東海岸がん臨床試験グループ
eGFR	estimated glemerular filtration rate	推定糸球体濾過量
FT3	free triiodothyronine3	遊離トリヨードサイロニン 3
FT4	free triiodothyronine4	遊離トリヨードサイロニン 4
EORTC	The European Organization for Research and Treatment of Cancer	全ヨーロッパにおけるがん・白血病研究組織
EQ-5D	EuroQol 5 Dimension	―

FAS	full analysis set	最大の解析対象集団
FDA	Food and Drug Administration	米国食品医薬品局
GCP	good clinical practice	医薬品の臨床試験の実施に関する基準
G-CSF	granulocyte colony stimulating factor	顆粒球コロニー刺激因子
γ-GTP	γ-glutamyl transpeptidase	ガンマ-グルタミル・トランスペプチターゼ
HBc	Hepatitis B core	B 型肝炎核
HBs	Hepatitis B surface	B 型肝炎表面
HCV	Hepatitis C Virus	C 型肝炎ウイルス
hCG	human chorionic gonadotropin	血液中ヒト絨毛性ゴナドトロピン
HIV	Human Immunodeficiency Virus	ヒト免疫不全ウイルス
ICD	International Classification of Diseases for Oncology	国際疾病分類腫瘍学
JCOG	Japan Clinical Oncology Group	日本臨床腫瘍研究グループ
K	potassium	カリウム
LDH	lactate dehydrogenase	乳酸脱水素酵素
MedDRA/J	Medical Dictionary for Regulatory Activities/J	ICH 国際医薬用語集日本語版
MEN2	Multiple Endocrine Neoplasia type 2	多発性内分泌腫瘍 2 型
MRI	magnetic resonance imaging	核磁気共鳴画像
MST	median survival time	生存期間中央値
MTD	Maximum tolerated dose	最大耐量
Na	sodium	ナトリウム
NET	norepinephrine transporter	ノルエピネフリントランスポーター
NF1	neurofibromatosis type1	神経線維腫症 1 型
NYHA	New York Heart Association	心不全の重症度分類
ORR	overall response rate	奏効率
OS	overall survival	全生存期間
PCC	pheochromocytoma	褐色細胞腫

PD	progressive disease	進行
PDF	portable document format	ポータブル・ドキュメント・フォーマット
PET	positron emission tomography	陽電子放射断層撮影
PFS	progression free survival	無増悪生存期間
PGL	paraganglioma	パラガングリオーマ
PPGL	pheochromocytoma paraganglioma	褐色細胞腫/パラガングリオーマ
PPS	per protocol set	治験実施計画書適合集団
PR	partial response	部分寛解
RPK	radiopharmacokinetics	放射能薬物動態
PRO	patient reported outcome	患者報告アウトカム
PS	Performance Status	パフォーマンス・ステータス
QA	quality assurance	品質保証
QOL	quality of life	生活の質
RD	recommended dose	推奨投与量
RECIST	response evaluation criteria in solid tumor	固形がんの治療効果判定のためのガイドライン
SAE	serious adverse event	重篤な有害事象
SAS	safety analysis set	安全性解析対象集団
SD	stable disease	（病勢）安定
SpO2	saturation of percutaneous oxygen	血中酸素飽和濃度
STD10	severely toxic dose in 10% of animals	マウスにおける 10%に重篤な毒性が発現する投与量
TSH	thyroid stimulating hormone	甲状腺刺激ホルモン
t1/2	Biological half-life	血中濃度半減期
VHL	von Hippel Lindau	von Hippel Lindau 病
WHO	World Health Organization	世界保健機関

治験デザインの要約

治験名	^{211}At -MABG の褐色細胞腫/パラガングリオーマ患者における薬物動態、安全性および有効性の評価
治験の目的	自家製造した、α線放出核種アスタチン-211（^{211}At）を結合させた 3-アスタトベンジルグアニジン（MABG）を、褐色細胞腫/パラガングリオーマの患者に投与した際の忍容性につき評価し、最大耐量（MTD）および推奨投与量（RD）を決定する。また放射能薬物動態および認容性、安全性を評価し、有効性について探索的に評価する
治験実施の根拠	悪性褐色細胞腫/パラガングリオーマは副腎髄質に発生する腫瘍である。診断、治療におけるエビデンスは希少疾患であるため十分でなく、新たな治療法が望まれている。がん細胞を殺傷する能力が高いα線を放出する核種 ^{211}At を結合させた薬剤、^{211}At-MABG がマウスにおいて悪性褐色細胞腫/パラガングリオーマに対して高い治療効果を示すことが示唆された。本薬のヒト褐色細胞腫/パラガングリオーマにおいても抗腫瘍効果が期待される。
治験の相	第Ⅰ相
治験デザイン	用量漸増試験
対象	褐色細胞腫患者/パラガングリオーマ患者
選択基準	以下のすべての条件を満たすものとする。<br>1) 本人より文書による同意取得が可能な患者<br>2) 褐色細胞腫、パラガングリオーマ、悪性褐色細胞腫、悪性パラガングリオーマいずれかと組織学的または臨床学的に診断されている患者<br>※診断基準については「褐色細胞腫・パラガングリオーマ診療ガイドライン 2018」に従うこととする。<br>3) 以下に定義する褐色細胞腫と診断されている患者（下記①～③のいずれかを満たし、かつ外科的切除や根治的放射線外照射が不可能なものを難治性褐色細胞腫と定義する）<br>① 初発時に原発巣の高度な局所進展を有する褐色細胞腫/パラガングリオーマ<br>② 初発時に遠隔転移を有する悪性褐色細胞腫/悪性パラガングリオーマ

	③ 外科的切除を行うも局所再発または遠隔転移再発を来した褐色細胞腫/パラガングリオーマ<br>4) 同意取得時の年齢が 20 歳以上の患者であること<br>5) ECOG Performance status（PS）が 0〜2 の患者であること<br>6) スクリーニング時に実施された CT 画像で確認された標的病変の 1 つ以上で、スクリーニング時に実施された ^{123}I-MIBG シンチグラフィで集積陽性が確認されている患者<br>7) スクリーニング時の検査値が、以下の基準をすべて満たす患者であること<br>① 骨髄機能<br>(1) 顆粒球コロニー刺激因子（G-CSF）製剤非投与下で白血球数≧3,000 /μL<br>(2) 非輸血下でヘモグロビン≧9.0 g/dL<br>(3) 非輸血下で血小板数≧10×10^4/mm^3<br><br>② 腎機能<br>(1) 推定糸球体濾過量（eGFR）≧30mL/min/1.73m^2<br>③ 肝機能<br>(1) AST≦90 U/L<br>(2) ALT≦126 U/L（男性）、ALT≦69 U/L（女性）<br>(3) LDH＜666 U/L<br>④ 心機能<br>(1) NYHA Functional class：Ⅰ以下<br>⑤ 糖尿病・内分泌疾患<br>(1) HbA1c＜8.0%（NGSP 値）<br>⑥ 呼吸状態<br>(1) 大気中における血中酸素飽和濃度（SpO2）≧96%<br>8) 3 ヶ月以上の生存が期待できる患者<br>9) 核医学治療病室内隔離期間中、摂食、排泄および睡眠の自立が見込める患者<br>10) 標準的な治療法（CVD 療法または ^{131}I-MIBG 治療）で効果がなかった、または他に適切な治療法がない患者
除外基準	以下の項目のいずれかに該当する患者は除外する。<br>1) 活動性の重複がんを有する患者<br>ここでの活動性の重複がんとは、同時性重複がん/多発がんおよび無病期間が 5 年以内の異時性重複がん/多発がんとする。た

だし、局所治療により治癒と判断され、治癒後の無病期間が1年を超えた上皮内がんや粘膜内がん相当の病変は、活動性の重複がん/多発がんに含めない。また、以下に示す家族性褐色細胞腫として合併するがんは活動性の重複がん/多発がんに含めない。

① 多発性内分泌腫瘍 2 型（MEN2）における甲状腺髄様がん

② von Hippel Lindau 病（VHL）における網膜血管芽腫

③ 神経線維腫症 1 型（NF1）における神経線維腫

2) MABG の集積を抑制する薬剤を所定の期間、中止することができない患者

3) 所定の期間α-メチルパラチロシンの服用を中止することができない患者

4) 登録前 8 週間以内に手術、CVD 療法、肝転移に対するカテーテル肝動脈塞栓療法、放射線療法を受けたことがある患者

5) 登録前 12 週間以内に MIBG 治療を受けたことのある患者

6) 前治療時において治療時あるいは治療後に治療と因果関係を否定できないグレード 2 以上の非血液毒性の出現を認め、治験期間中に加療を要する患者

7) 以下のいずれかの感染症がありかつ治験期間中に加療を要する患者

① B 型肝炎ウイルス感染症

② C 型肝炎ウイルス感染症

③ HIV 感染症

④ その他全身治療を要する感染症

8) 副腎皮質ホルモン（プレドニンもしくはプレドニゾロン換算量 10 mg 以上/日）またはその他の免疫抑制剤の持続的な全身投与を必要とする疾患がありかつ治験期間中にも加療を要する患者

9) コントロール困難なカテコラミン発作の既往がある患者

10) 致死性不整脈もしくは心停止の既往のある患者

11) コントロール不良の症候性不整脈、甲状腺機能異常、呼吸器疾患、胸腹水貯留のある患者

12) 冠動脈疾患、アミオダロンによる加療を要する不整脈、重症心弁膜症、大動脈疾患、出血傾向がある疾患・状態がある患者

13) 妊娠中(医師の問診等により妊娠している可能性があると判断された場合にも、当該患者を本試験から除外)、産後 28 日以内または授乳中（授乳中の女性が授乳を中断しても不可）の患者

	14) 妊娠する可能性がある女性患者あるいはパートナーが妊娠する可能性がある男性患者で投薬治療後 6 ヶ月間避妊することに合意できない患者（避妊法としては、ラテックス製コンドーム（男性が使用）、各種ホルモン避妊薬（経口避妊薬等）、子宮内避妊具（IUD）（プロゲステロン無添加 T 型）のいずれか 2 種類以上を併用するか、卵管結紮及び精管切除）<br>15) 同意日の前 3 か月以内に他の治験に参加している患者<br>16) その他、治験責任医師または治験分担医師が本治験の対象として適切ではないと判断した患者
治験薬	3-アスタトベンジルグアニジン(211At)（^{211}At-MABG）（自家製造）
試験デザイン	本治験は、褐色細胞腫/パラガングリオーマ患者を対象に ^{211}At-MABG を単回静脈内ボーラス投与した場合の忍容性について評価し、本剤の RD を決定することを目的とした単群オープン試験である。本剤の投与量は低用量（0.65MBq/kg）、中用量（1.3MBq/kg）および高用量（2.6MBq/kg）とし、低用量から開始して 3+3 デザインに従って用量を検討する。
治験期間	同意取得日～投与後 12 週
併用禁止薬剤／併用禁止療法	1) 手術療法<br>2) 化学療法<br>3) 顆粒球コロニー刺激因子（G-CSF）<br>※使用歴がある場合には最後の使用から 8 日以上経過していること<br>4) ホルモン療法<br>5) 抗体療法<br>6) 放射線療法（核医学治療も含む）<br>7) その他の抗腫瘍療法（いわゆる免疫療法を含む）<br>8) 副腎皮質ステロイド剤の継続的な全身投与（プレドニン換算で 10 mg/日を超えるもの）<br>9) 免疫抑制剤あるいは免疫賦活剤の全身投与<br>10) 治験薬投与部位への副腎皮質ステロイド剤の局所使用<br>11) 抗腫瘍効果を目的とした健康食品（アガリスク等）等の摂取<br>12) 抗不整脈薬（心室性不整脈）<br>13) 交感神経遮断薬（$\alpha\beta$遮断薬、α遮断薬、アドレナリン作動性神経遮断薬）<br>14) カルシウム拮抗薬：アムロジピン、ジルチアゼム等<br>15) 交感神経作動薬（変力性、血管収縮性、β_2 作動性）

16) アドレナリン受容体作動薬

17) 抗うつ薬

18) 抗精神病薬（神経遮断薬）

19) 中枢神経刺激薬

20) 治験薬投与前の輸血

※輸血歴がある場合には、最後の輸血から 29 日以上経過していること

評価項目

【主要評価項目】

安全性（用量制限毒性:DLT、最大耐量：MTD、推奨用量：RD）

【副次的評価項目】

1) 放射能薬物動態（RPK）
2) 尿中放射能排出率
3) 尿中カテコラミン類の奏効率
4) 奏効率（ORR）
5) 無増悪生存期間（PFS）
6) ^{123}I-MIBG シンチグラフィによる腫瘍集積減少効果
7) QOL

調査・観察・検査およびスケジュール

調査/検査項目	投与前				投与日			投与後									
	同意	スクリーニング期間	被験者登録	7日以内	投与前	投与	投与終了後	投与翌日	2日後	3日後	7日後	2週	4週	6週	8週	12週	治験中止時[*14]
		Day-42～-14	～Day-14	Day-7～-1	Day1			Day2	Day3	Day4	Day8	Day15	Day29±7	Day43±7	Day57±7	Day85±7	±14
		入院			入院									入院		入院	入院
文書同意取得	●																
登録			●[*12]														
^{211}At-MABG投与						●											
ヨウ化カリウム投与				●[*13]	●[*13]			●[*13]									
5-HT$_3$受容体拮抗剤投与					●												
被験者背景の調査		●															
併用薬/併用療法の調査			●→													→	●
**全身状態**																	
ECOG Performance Status		●		●				●→				→●	●	●	●	●	●
身長		●															
体重		●		●								●	●	●	●	●	●
診察所見		●		●	●		投与直後、1,2,3,4,6時間後	●→				→●	●	●	●	●	●
バイタルサイン[*1]		●		●	●		投与直後、1,2,3,4,6時間後	●→				→●	●	●	●	●	●
血中酸素飽和度(SpO$_2$)		●		●	●		投与直後、1,2,3,4,6時間後	●→				→●	●	●	●	●	●
**血液生化学検査**																	
血液学的検査[*2]		●		●	●		1,3,6時間後	●		●	●	●	●	●	●	●	●
血液生化学[*3]		●		●	●		1,3,6時間後	●		●	●	●		●		●	●
尿[*4]		●		●										●		●	●
感染症検査[*5]		●															
心電図検査（12誘導）		●			●		投与後５分後、1,3,６時間後	●		●	●	●		●		●	●
心臓超音波検査		●												●		●	●
尿中カテコラミン類検査[*6]		●												●		●	●
^{123}I-MIBGシンチグラフィ[*7]		●												●		●	●
^{123}I-MIBG集積の確認[*8]		●															
QOL調査[*9]		●												●		●	●
**放射能薬物動態**																	
血中放射能濃度測定					●		投与後5分、15分、1,3,６時間後	24時間後	48時間後	72時間後							
尿中放射能濃度測定					●		投与後0-3,3-6,6-12時間	12-24時間	24-48時間	48-72時間							
CT検査[*10]		●												●		●	●
妊娠検査（hCG）[*11]		●															
有害事象			●→													→	●

目標症例数

最大 18 例

治験実施予定期間

2022 年 4 月～2024 年 1 月

目次

略号一覧 .... －1－
治験デザインの要約 .... －4－
1　開発の経緯および背景 .... －12－
　1.1　開発の経緯 .... －12－
　1.2　非臨床試験成績 .... －18－
　1.3　臨床試験成績 .... －20－
　1.4　潜在的なリスクベネフィット評価 .... －20－
2　治験の目的 .... －20－
　2.1　治験の目的 .... －20－
　2.2　治験の種類 .... －20－
3　治験の計画 .... －21－
　3.1　試験デザイン .... －21－
　3.2　治験期間 .... －21－
　3.3　用法・用量の検討および用量制限毒性（Dose Limiting Toxicity：DLT）の定義　－21－
　3.4　用量制限毒性（Dose Limiting Toxicity：DLT）の定義 .... －22－
　3.6　評価項目 .... －23－
　3.7　治験実施予定期間 .... －24－
　3.8　症例数 .... －24－
4　前治療、併用薬剤・併用療法、後治療 .... －25－
　4.1　前治療 .... －25－
　4.2　併用薬剤・併用療法 .... －25－
5　被験者に対する同意・説明文書の作成と改訂 .... －26－
6　対象被験者 .... －26－
　6.1　対象被験者 .... －26－
　6.2　選択基準 .... －26－
　6.3　除外基準 .... －28－
7　同意取得の時期と方法 .... －29－
　7.1　登録前 .... －29－
　7.2　被験者の意思に影響を与える情報入手時 .... －30－
　7.3　同意文書およびその他の説明文書の改訂時 .... －30－
　7.4　被験者の他科および他院受診に際して .... －30－
8　被験者の登録 .... －30－
　8.1　症例登録の手順 .... －30－

8.2 追加登録 - 31 -
9 調査・観察・検査、評価項目および実施時期 - 31 -
9.1 調査・観察検査および実施時期 - 31 -
9.2 同意の取得 - 34 -
9.3 被験者識別コードの割り当て - 34 -
9.4 入院及び核医学治療室への入室 - 34 -
9.5 治験期間中に実施する調査・観察・検査 - 34 -
9.6 評価 - 41 -
10 中止・終了基準 - 43 -
10.1 中止・終了手順 - 43 -
10.2 被験者毎の完了および中止 - 44 -
11 被験者の安全性の確保 - 44 -
11.1 有害事象に関する定義 - 44 -
11.2 有害事象の記録 - 47 -
11.3 有害事象の予測性 - 48 -
11.4 有害事象の発現、本剤投与後に妊娠が判明した場合の措置 - 48 -
12 治験薬情報 - 50 -
12.1 原薬 - 50 -
12.2 治験薬 - 51 -
13 治験薬の管理 - 52 -
13.1 治験薬の交付・保管・管理・回収 - 52 -
13.2 治験薬の無菌性試験 - 52 -
14 統計解析 - 52 -
14.1 症例数 - 53 -
14.2 解析対象集団 - 53 -
14.3 データの取り扱い - 54 -
14.4 解析方法 - 54 -
15 治験実施計画書の遵守および逸脱・変更並びに改訂 - 56 -
15.1 治験実施計画書の承認および改訂 - 56 -
15.2 治験実施計画書の遵守 - 56 -
15.3 治験実施計画書からの逸脱または変更 - 57 -
16 治験の終了または中止および中断 - 57 -
16.1 治験の終了 - 57 -
16.2 治験の早期中止・中断 - 57 -
17 症例報告書 - 58 -
17.1 様式 - 58 -

17.2　記入方法 - 58 -
17.3　提出方法 - 58 -
18 モニタリング - 58 -
18.1　モニタリングの方法 - 58 -
18.2　原資料およびその他の記録の特定 - 59 -
19 治験の品質管理および品質保証 - 59 -
19.1　原資料の直接閲覧の保証 - 60 -
19.2　効果安全性評価委員会 - 60 -
20 記録等の保存 - 60 -
21 金銭の支払いおよび保険 - 61 -
21.1　治験に関する費用と被験者負担について - 61 -
21.2　健康被害に対する補償について - 61 -
22 公表に関する取り決め - 61 -
23 治験の倫理的および科学的実施 - 61 -
23.1　遵守すべき諸規則 - 61 -
23.2　説明文書・同意文書の作成と改訂 - 61 -
23.3　説明と同意（インフォームド・コンセント） - 62 -
23.4　個人情報とプライバシーの保護 - 62 -
24 治験責任医師の責務 - 63 -
25 治験実施体制 - 64 -
25.1　治験実施医療機関 - 64 -
25.2　治験責任医師 - 64 -
25.3　治験・開発計画支援責任者 - 64 -
25.4　治験薬製造者 - 64 -
25.5　統計解析責任者 - 65 -
25.6　薬物動体解析責任者 - 65 -
25.7　効果安全性評価委員 - 65 -
25.8　その他の治験管理組織 - 65 -
26 引用文献 - 66 -
27 付録 - 69 -

1　開発の経緯および背景

1.1　開発の経緯

1.1.1　褐色細胞腫/パラガングリオーマについて

褐色細胞腫/パラガングリオーマは、副腎髄質あるいは傍神経節のカテコラミン産生クロム親和性細胞から発生する腫瘍で、前者を褐色細胞腫（PCC）、後者をパラガングリオーマ（PGL）、両者を総称して褐色細胞腫/パラガングリオーマ（PPGL）と呼ぶ。古くから神経内分泌腫瘍の一つ [1)] とされている。2017 年に発表された内分泌腫瘍の WHO 腫瘍分類 [2)] では潜在的に転移性で「良性」である確実な証拠がないことから、すべての PPGL に悪性腫瘍の疾患コード（ICD-3）が付与され、「良性」の表現は使用しないことになった。すなわち、診断時に明らかな転移性あるいは局所浸潤性病変を認めない場合は単に「PPGL」と診断し、平行して悪性度 [3)] の評価を行うことが推奨される。一方、明らかな転移性あるいは局所浸潤性病変を有する場合は、これらいずれの病態を包括しかつ臨床現場の用語の混乱を避けるため「悪性」の表現を用いる。

PGL には交感神経系由来と副交感神経系由来があり、前者は主に腹部・骨盤部に発生し、ノルアドレナリン産生性で悪性度が高いのに対し、後者は主に頭頸部に発生し、ホルモン産生能が低く、悪性度も低い。

1.1.2　症状について

褐色細胞腫/パラガングリオーマでは、高血圧、動悸、頻脈、胸痛、頭痛、顔面蒼白、発汗、不安など、多彩な臨床症状を示す。代謝面では高血糖、乳酸アシドーシス、体重減少を認めることが多い。高血圧は発作型、持続型、混合型がある。時に種々の誘因（食事、排尿、麻酔、腫瘍摘出、薬物＜β遮断薬、高用量デキサメタゾン、グルカゴン、造影剤、チラミン、メトクロプラミド、三環性抗うつ薬など＞）により高血圧クリーゼを呈する。一方で、無症候性、正常血圧性で副腎偶発腫瘍として発見される例も少なくない。実際に、副腎偶発腫瘍の 5～10%は PCC であり、近年では PCC 前例の約 25%は副腎偶発腫瘍として発見されている。ドパミン産生性 PGL では、正常血圧～低血圧を呈することがある。また、原因不明の起立性低血圧を示す例もある。アドレナリン産生性腫瘍では低血圧やショック症状を呈することがある。その機序として血管内容量の減少、腫瘍壊死によるカテコラミン分泌の急な低下、アドレナリン受容体の脱感作、低カルシウム血症などがあげられる。時に、心筋梗塞、不整脈、大動脈解離、腫瘍内壊死、腫瘍破裂などによりショックを来す例もある。その他、タコツボ心筋症、高血圧性脳症、脳血管疾患による突然死、心不全などもある。

1.1.3　スクリーニング

PPGL の既往歴ないし家族歴、PCC を伴う遺伝性疾患（多発性内分泌腫瘍 2 型（MEN2）、von Hippel Lindau 病（VHL）、神経線維腫Ⅰ型（NF1））、動悸、発汗、頭痛、胸痛などの多様な症状（spells）、発作性高血圧、治療抵抗性高血圧、糖尿病合併の高血圧、副腎偶発腫瘍などは、臨床的に「PPGL ハイリスク群」と位置付けられ、積極的にスクリーニングすべき対象疾患[4)]である。通常の高血圧患者におけるスクリーニングの費用対効果は確立していない。

1.1.4　疫学

褐色細胞腫の正確な有病率は不明である。オーストラリアにおける報告[5)]では、成人 100 万人当たり、年間に 1.55 名とされている。これは、成人の 20%が高血圧と仮定すると、年間、100 万人の高血圧患者の中で褐色細胞腫は約 5 人という計算になる。しかし、多くの患者は、腹痛、嘔吐、呼吸困難、低血圧などの非特異的症状を呈すことから、一生涯のうちに褐色細胞腫と診断されない例も少なくない。わが国では厚生省（当時）「副腎ホルモン産生異常症調査研究班」（竹田班、名和田班）により全国疫学調査が実施されている（表 1-1）。

表 1-1　褐色細胞腫/パラガングリオーマに関する疫学調査の比較

	副腎ホルモン産生異常症調査研究班（竹田班）[*1)]	副腎ホルモン産生異常症調査研究班（名和田班）[*2)]	褐色細胞腫の実態調査と診療指針の作成研究班（成瀬班）[*3)]
調査期間	1973～1982 年（10 年間）	1997 年（1 年間）	2008 年（1 年間）
対象施設	300 床以上の病院	すべての大学病院・一般病院から層別無作為抽出	すべての大学病院・一般病院から層別無作為抽出
対象診療科	内科、小児科、外科、泌尿器科	内科、内分泌代謝科、小児科、脳外科、泌尿器科	内科、循環器科、外科、小児科、泌尿器科、放射線科、小児外科
対象診療科数		4,060	5,912（院内標榜 6,303）
一次調査症例数	862	522	1，649
推計患者数		1,030	2,920

[95%信頼区間]		[860～1,200]	[2,580～3,260]

*1) 厚生省特定疾患内分泌系疾患調査研究班「褐色細胞腫の全国集計および第 3 期ステロイドホルモン産生異常症の全国集計について」

*2) 厚生省特定疾患内分泌系疾患調査研究班「副腎ホルモン産生異常症の全国疫学調査」

*3) 厚生労働科学研究費補助金難治性疾患克服研究事業「褐色細胞腫の実態調査と診療指針の作成」

平成 21 年度に実施された全国疫学調査（成瀬班）から推定される患者数は良性 2,600 例、悪性 320 例となっている。男女差は認められず推定発症年齢は 40～45 歳であるが幅広い年齢層に分布している。症候性（高血圧あり）は約 65%、無症候性は約 35%で、副腎偶発腫瘍としても発見されている。副腎外、両側性、悪性は各々約 10%、家族歴のあるものは約 5%であった。
^{131}I-MIBG あるいは ^{123}I-MIBG シンチグラフィは約 10%で偽陰性であった。

1.1.5 **遺伝性について**

褐色細胞腫は、発症した原因の約 30～40%が遺伝によるもの（家族性腫瘍）と報告されている。SDHB・SDHD・VHL・RET・NF1 と呼ばれる遺伝子をはじめとし、現在では 10 種類以上の、褐色細胞腫と関係した遺伝子の変化が明らかになっている。遺伝子の変化が見つかることで、フォローアップの方針検討や血縁者の発症リスク特定に役立つ。多発性内分泌腫瘍Ⅱ型・Von Hippel Linfau 病（フォン・ヒッペルーリンダウ病）・レックリングハウゼン病などの遺伝子疾患と判明すれば、褐色細胞腫以外の疾患の併発チェックなど、継続的なサーベイランスなどが有効な可能性がある。しかし、遺伝子変化の同定により家族への遺伝の可能性など、考慮すべき点が増える可能性があり、遺伝子検査の前後にはその結果わかることと懸念事項について、十分な遺伝カウンセリングが求められる。

1.1.6 **診断**

副腎腫瘍の精査として、カテコラミンを過剰に産生しているかどうかを評価するために、カテコラミンおよびその代謝物を血中・尿中で測定する。また、腫瘍の位置や広がりを評価するために CT や MRI、^{123}I-MIBG シンチグラフィー、FDG-PET-CT、オクトレオチドシンチグラフィーなどを行う。

1.1.7 **治療**

治療には、腫瘍そのものに対する治療と、カテコラミン過剰症状に対する治療が必要となる。

腫瘍そのものに対する治療の第一選択は手術による腫瘍摘出である。

手術には血圧や体液量管理が重要であり、降圧剤（α遮断薬）、補液の投与など全身管理を慎重に行う。手術後は、術後再発のチェックのために、定期的な画像検査や採血などを行う。

手術で切除困難なケースや、他の内臓に病巣を認めたり、術後に再発をきたした際は、抗がん剤治療を行うことがある。抗がん剤治療の内容としては、CVD 療法（シクロフォスファミド、ビンクリスチン、ダカルバジン）の治療成績が最も多く報告されている。CVD 療法により、約半数の症例で腫瘍の縮小が認められると報告されており、症状改善など、短中期的に QOL 改善に繋がる可能性がある。しかし、効果に関したデータは乏しく、治療法については慎重に決定することが勧められる。

なお、2021 年９月に本邦において ^{131}I-MIBG 製剤が「MIBG 集積陽性の治癒切除不能な褐色細胞腫・パラガングリオーマ」を適応として承認された。

カテコラミン過剰生産による症状のコントロールは、それぞれの症状に対する治療を行う。例えば、高血圧に対しては降圧剤を内服する。しかし、降圧剤のみではコントロールが難しい場合、カテコラミン合成酵素阻害剤（メチロシン）を使用する。高度便秘や腸閉塞を来した場合、フェントラミン（α遮断薬）も使用される。

薬剤投与、腹部の触診、侵襲的な検査・処置をきっかけにカテコラミン分泌が急に上昇し、褐色細胞腫クリーゼと呼ばれる血圧の著しい上昇をきたして、放置すると臓器障害で致命的となる場合があるため、診察や投薬の際には注意が必要となる。

1.1.8 悪性度の評価法

PPGL は潜在的に悪性であることを考慮して、2017 年 WHO の内分泌腫瘍分類[2)]にて PPGL は「悪性腫瘍」の範疇に分類されたことから、長期にわたる慎重な経過観察が必要である[4)]。非クロマフィン組織（骨、肺、肝、リンパ節など）への転移が存在すれば確実に"悪性"と診断されるが、転移巣が名家腕内段階では進行性の増悪、予後不良の指標となる悪性度の評価が重要である。悪性度の評価指標を表 1-2 に示す。

表 1-2 PPGL の悪性度の評価指標

	悪性・進行性増悪・予後不良を示唆する所見
性別	男性
診断年齢	中高年以上
腫瘍サイズ	大（4～5cm 以上）
分泌カテコラミンパターン	ノルアドレナリン性・ドパミン性
転移巣診断の時期	原発巣と同時期（synchronous）
腫瘍の局在・病型	腹部・骨盤部パラガングリオーマ
^{18}F-FDG-PET	集積あり

病理組織マーカー	Ki67 染色陽性率＞5%
	GAPP　7〜10 点
	SDHB 染色陰性
遺伝子マーカー	SDHB 遺伝子変異陽性

1) 臨床所見

男性、初回診断時の年齢（特に 76 歳以上）、腫瘍サイズ（6cm 以上）[6]、ドパミン、ノルアドレナリン過剰[7]、原発巣の手術が不可、などが臨床的な悪性度の指標と報告[3、8]されている。原発巣と転移巣診断の時期も重要[6、9]とされ、原発巣の診断、手術後 6 ヶ月以降に転移が確認された場合と比較して、同時期に診断された例の予後は不良とされる。多重ロジスティック回帰分析により、45 歳以上と初回診断時の転移の存在が独立した生命予後の規定因子との報告もある[10]。

腫瘍の局在部位、病型と悪性度の関連も報告されている。両側性では片側性より悪性の頻度が高く（21.4% vs 10.2%）、PGL は PCC よりも悪性の頻度が高い（40% vs 10%）[9]。PGL が最も重要な予後規定因子との報告[6]もある。PGL の中では腹部・骨盤部 PGL は約 40%が悪性であるのに対して、頭頸部 PGL では 3〜5%である[11]。画像検査（MRI、CT、^{131}I-MIBG シンチグラフィ）で非クロマフィン組織（肝臓、骨、肺、リンパ節など）に病変が存在すれば悪性の根拠となるが、傍大動脈神経節の病変はリンパ節転移との鑑別が困難である。^{18}F-FDG-PET は良性腫瘍よりも転移性腫瘍で陽性率が高く（82%）、特に SDHB 遺伝子変異陽性の PGL で腫瘍検出率が高い[12]が、良性腫瘍（58%）でも取り込みを認め特異性に限界がある。

2) 病理組織マーカー

病理学的に悪性所見とされる被膜浸潤、脈管浸潤は良性 PPGL でも認めるため、鑑別には役立たない。腫瘍増殖能の指標である Ki67 免疫染色性が有用とされ、陽性細胞率が 3%以上は悪性を示唆する[13]。PCC より PGL での陽性頻度が高い。組織所見のスコア化により悪性度を診断する PASS[14]は、PASS 4 点以上では臨床的に悪性、PASS 4 点未満は良性の経過を示すとされるが、予後との関連に否定的な報告[15]もある。Kimura ら[16), 17]はスコアリングスケール（GAPP）による分化度と 5 年生存率、予後が相関することを報告している。

3) 遺伝子マーカー

散発性 PPGL の 20〜30%に遺伝子変異を有する遺伝性であることが報告されている。特に悪性度との関連が深いのが SDHB 遺伝子変異である。SDHB 遺

伝子変異陽性例での悪性の頻度は 50～90%[18] 、悪性 PPGL の 5～50%が SDHB 遺伝子変異陽性で予後不良と報告されている。

4) バイオマーカーの組合わせによる予知法
複数のマーカー、すなわち①原発腫瘍の腫瘍系、②副腎性か副腎外性か、③分泌カテコラミン型（アドレナリン型かノルアドレナリン型か）、④腫瘍の被膜への浸潤の有無、⑤SDHB 遺伝子変異の有無、⑥受容体型チロシンキナーゼであるヒト EGFR2 型（ERBB-2）過剰発現の有無、などの所見のスコア化により、悪性度の半定量的予知が可能であると報告 [19] されている。

1.1.9 **現状治療の問題**

褐色細胞腫・パラガングリオーマ診療ガイドライン 2018[20] によると、手術療法による腫瘍切除術が第一選択となる（1A）。その際、α1 遮断薬などによる十分な術前処置と経験豊富な麻酔科医による厳重な術中管理が不可欠となる（1B）。さらに、常に悪性の可能性を念頭に置き、腫瘍被膜を損傷しないように細心の注意が必要となる（2C）。比較的小さい褐色細胞腫に対しては腹腔鏡下副腎摘除術が標準術式である（1B）が、悪性度が高いと考えられる症例では、開腹手術を選択する（2C）。治癒切除ができない場合でも、原発巣切除術が推奨される（2C）。悪性の頻度が低い家族性褐色細胞腫・パラガングリオーマや対側副腎摘除後の症例では、副腎部分切除術も推奨される（2C）。

悪性褐色細胞腫・パラガングリオーマに対しては、CVD 療法が、実施が比較的容易かつ副作用も軽～中等度であることから、第一選択となる（2C）。CVD 治療の腫瘍容積とカテコラミン過剰の減少効果（完全奏功と部分奏功）は 40～50%である。CVD 治療は悪性褐色細胞腫・パラガングリオーマの無増悪生存期間（PFS）を延長するが、生命予後を改善させる明確なエビデンスはない。

一方、^{123}I-MIBG が集積する悪性褐色細胞腫・パラガングリオーマに対しては、ガイドラインでは以下の記載がある。^{131}I-MIBG 治療が適応となる（1B）。腫瘍縮小を目的とする ^{131}I-MIBG 治療は低用量（～7.4GBq）でも有用であるが、中用量以上（7.4GBq～）がより望ましい（2C）。本邦で施行可能な低用量（～7.4GBq）による ^{131}I-MIBG 治療の複数回投与法は、奏効率を向上させる（2C）。低用量 ^{131}I-MIBG 治療は過剰カテコラミンの抑制に有用である（2C）。^{131}I-MIBG 治療では低用量で 10～20%の Grade 2 以下の血液毒性、中用量で 50%未満の Grade 3 血液毒性、高用量（14.8GBq～）で 80%以上の Grade 3 以上の血液毒性が生じえる。^{131}I-MIBG 治療の際には、甲状腺機能低下症の軽減のために無機ヨウ素による甲状腺ブロックが

推奨される(1B)。国内治験の17例での成績では 7.4GBq 単回投与にて、尿中カテコラミン類の低下を23.5%に認め、RECIST による奏功率は 5.9%であった。

1.1.10 **^{211}At-MABG** について

アスタチン(At)はハロゲン族に属する元素で、ギリシャ語の「不安定」を意味する「astatos(アスタトス)」が名前の由来であり、その名の通りアスタチンは放射線を放出し別の元素に壊変する性質を有する。なかでも ^{211}At はα線を放出する核種であり、安定元素、鉛-207(^{207}Pb)に壊変する(半減期 7.2 時間)。*meta*-astatobenzylguanidine(MABG) は、ノルエピネフリンと似た化学構造を有するベンジルグアニジンという物質に ^{211}At を組み込んだ薬剤であり、褐色細胞腫がノルエピネフリンを取り込むノルエピネフリントランスポーター(NET)の基質となることから、NET を介して褐色細胞腫に取り込まれる。また、褐色細胞腫細胞では NET 発現が通常の細胞に比べて亢進しており、多量の ^{211}At-MABG が細胞内に取り込まれ、また褐色細胞腫細胞特異的に、細胞内の分泌小胞内に貯蔵されることから、^{211}At-MABG は褐色細胞腫細胞に取り込まれ、長時間にわたり細胞内にとどまることから、悪性褐色細胞腫/パラガングリオーマに対する新たな治療の選択肢となることが期待されている。

1.2 非臨床試験成績

1.2.1 薬理

^{211}At-MABG は in vitro においてヒト神経芽細胞腫細胞に対する殺細胞効果を示した。また、ラット褐色細胞腫細胞 PC12 を用いた in vitro 試験において、^{211}At-MABG は用量依存的に DNA 二重鎖切断を生じる細胞の割合を増加し、細胞生存率を低下させた。

PC12 細胞を皮下移植したマウスに ^{211}At-MABG 投与した時、^{211}At-MABG は PC12 細胞に高い集積を示し、PC12 細胞の増殖は用量依存的に抑制された。

1.2.2 **放射能薬物動態試験**

^{211}At-MABG をマウスに単回静脈内投与した時の体内分布傾向は ^{131}I-MIBG と同様な傾向を示し、NET が多く存在する心臓および副腎に高い集積を示した。

表 1-3 ^{211}At-MABG および ^{131}I-MIBG の正常マウスにおける体内分布 [21)]

	% injected dose per gram[a]					
	1 h		4 h		24 h	
tissue	[^{211}At]MABG	[^{131}I]MIBG[b]	[^{211}At]MABG	[^{131}I]MIBG[b]	[^{211}At]MABG	[^{131}I]MIBG[b]
liver	9.17 ± 0.80[c]	7.45 ± 1.06	4.81 ± 0.59	4.08 ± 0.70	1.54 ± 0.11[c]	0.95 ± 0.13
spleen	3.87 ± 0.69	4.55 ± 0.84	3.40 ± 0.42	3.22 ± 0.22	2.65 ± 0.88	1.82 ± 0.14
lungs	7.42 ± 1.84	7.62 ± 1.41	3.84 ± 0.77	3.69 ± 0.87	1.26 ± 0.32	1.10 ± 0.34
heart	20.61 ± 2.71	24.88 ± 3.75	12.97 ± 1.58[c]	16.14 ± 2.60	4.42 ± 0.51	3.75 ± 0.54
kidney	2.83 ± 0.29	2.52 ± 0.18	2.24 ± 0.21[c]	1.62 ± 0.19	1.01 ± 0.05[c]	0.72 ± 0.19
thyroid	2.83 ± 0.61	2.85 ± 0.99	2.24 ± 0.32	2.38 ± 0.50	2.47 ± 0.52	2.60 ± 0.34
blood	0.87 ± 0.05	0.99 ± 0.13	0.52 ± 0.11	0.52 ± 0.08	0.21 ± 0.06[c]	0.10 ± 0.02
adrenals	20.33 ± 5.06	16.85 ± 2.98	16.67 ± 1.91	16.15 ± 4.54	20.79 ± 5.53	26.73 ± 6.10

[a] Mean ± SD (n = 5). [b] No-carrier-added preparation. [c] Uptake of the two agents determined to be statistically significant by a two-sided t test: differences found to be significant are indicated ($p < 0.05$).

我々の実施した単回静脈内投与による正常マウスの薬物動態試験では、^{211}At-MABG は血液中から速やかに組織に移行し、血中放射能濃度は、半減期 $T_{1/2}\alpha$は約 11 分、$T_{1/2}\beta$は 13.6 時間の二相性で消失した。また、^{211}At-MABG 投与後の血液中の放射能分布は、血球画分で高く、また血漿液画分中の遊離未変化体は、投与 60 分後以降では検出されなかった。

^{211}At-MABG 投与後の排泄は、^{123}I-MIBG と同様な傾向を示したが、投与 6 時間後以降では ^{211}At-MABG が ^{123}I-MIBG よりも遅くなる傾向が見られた（24 時間で ^{211}At-MABG ：49.3%ID、^{123}I-MIBG ：60.9%ID）。

1.2.3 **拡張型単回投与毒性試験**

正常マウス（BALB/c）における ^{211}At-MABG の拡張型単回投与毒性試験を実施した。

マウスに 16MBq/kg（低用量）、48MBq/kg（中用量）あるいは 80MBq/kg（高用量）の ^{211}At-MABG を各 50 例（雄２５例、雌 25 例）ずつ単回静脈内投与し、最長 Day35 日までの経過の観察を行った。全体の中で死亡イベントは高用量群の雌(N=25)で 3 例（2 例は切迫安楽死）であった。

一般状態スコアは、用量依存的に悪化し、また、その悪化の期間も延長された。体重の推移も同様に、用量依存的に減少が見られ、高用量群においてはその回復も遅延していたが Day35 までには回復した。

臓器外観および病理組織学的検査では副腎の発赤・浮腫、肝細胞萎縮・炎症細胞浸潤などの異常所見の発現頻度が増加しており、その他、放射線への感受性が高いと考えられる消化管粘膜萎縮や造血組織における造血細胞減少などの異常所見が認められたが、Day35 までに回復あるいは回復傾向を示した。それに伴い、関連する血液学的および血液生化学的検査における項目の変動が認められたが Day35 までには回復した。

以上のように、高用量群において死亡例3例（2例は切迫安楽死）を認めたが、その他に不可逆的な毒性は認められず、マウスにおける 10%に重篤な毒性が発現する

投与量(severely toxic dose in 10% of animals、STD10)は 80MBq/kg 以上であると考えられた。

なお、一般状態の観察において、中枢神経系、呼吸器系および循環器系の重点観察項目を設定し、生命維持に重要な影響を及ぼす器官系に対する影響を検討したが、これらの項目における異常所見は認められなかった。

1.3 臨床試験成績

^{211}At-MABG 注射剤の臨床試験は未実施である。

1.4 潜在的なリスクベネフィット評価

^{211}At-MABG 注射剤の臨床試験は未実施であるが、本剤の非臨床試験及び β 線を放出する類薬 ^{131}I-MIBG の臨床試験成績より、腫瘍縮小効果やカテコラミン類の低下などの臨床効果が期待される。^{11}At は α 線放出核種であり、α線はβ線と比較して透過性が低く、半減期が短いため、本剤を投与された患者をアイソトープ治療室に長期間隔離する必要性がないメリットもある。β線薬剤では投与される放射能量が多くγ線も放出するため、緊急事の対応が難しいのに対し、α線薬剤は投与される放射能量が少ない。

副作用としては、骨髄抑制、消化管毒性、肝障害、カテコラミン放出によるクリーゼ等が予測される。

本試験に参加する他に適切な治療法がない患者にとっては、マウス STD10 の 1/10 のヒト換算量から投与量を検討することでリスクがベネフィットを大きく上回ることはないと考える。

2 治験の目的

2.1 治験の目的

α−線放出核種である ^{211}At を結合した ^{211}At-MABG の褐色細胞腫/パラガングリオーマ患者に投与し、その忍容性から MTD および RD を決定する。また、放射能薬物動態、安全性および探索的に有効性を検討する。

2.2 治験の種類

治験の種類:探索的試験

治験の相:第 I 相

3 治験の計画

3.1 試験デザイン

本治験は、褐色細胞腫/パラガングリオーマ患者を対象に ^{211}At-MABG を単回静脈内投与した場合の忍容性について評価し、本剤の MTD 及び RD を決定することを目的とした単群オープン試験である。本剤の投与量はコホート1（0.65MBq/kg）、コホート2（1.3MBq/kg）およびコホート3（2.6MBq/kg）とし、コホート1から開始して 3+3 デザインに従って RD を検討する。

3.2 治験期間

治験期間は各コホートとも同意取得日から投与後 12 週までとする。

3.2.1 治験治療期間

治験薬の投与日から投与後 12 週までとする。

3.3 用法・用量の検討および用量制限毒性（Dose Limiting Toxicity：DLT）の定義

3.3.1 用法・用量の定義

治験薬は患者に単回静脈内投与（ボーラス）を緩徐に行う。各コホートの投与量および増量計画については表 3-1 に示した。増量は一般的な 3+3 デザインに従う。コホート1より開始し、各コホートにおける予定症例数は 3 例から 6 例であり、2 例 DLT が発生した段階で症例の追加を中止とする。投与量の漸増方法について

図 3−1 に示した。なお、用量レベルにおいて 2/3 あるいは 2/6 例以上となった場合には1レベル下で 6 例までの追加の検討を行う。

表 3−1 レベルごとの投与量

コホート	用量レベル	投与量	予定症例数
コホート1	レベル 1	0.65MBq/kg	3～6 例
コホート2	レベル 2	1.3MBq/kg	3～6 例
コホート3	レベル 3	2.6MBq/kg	3～6 例

図 3−1 投与量の漸増方法

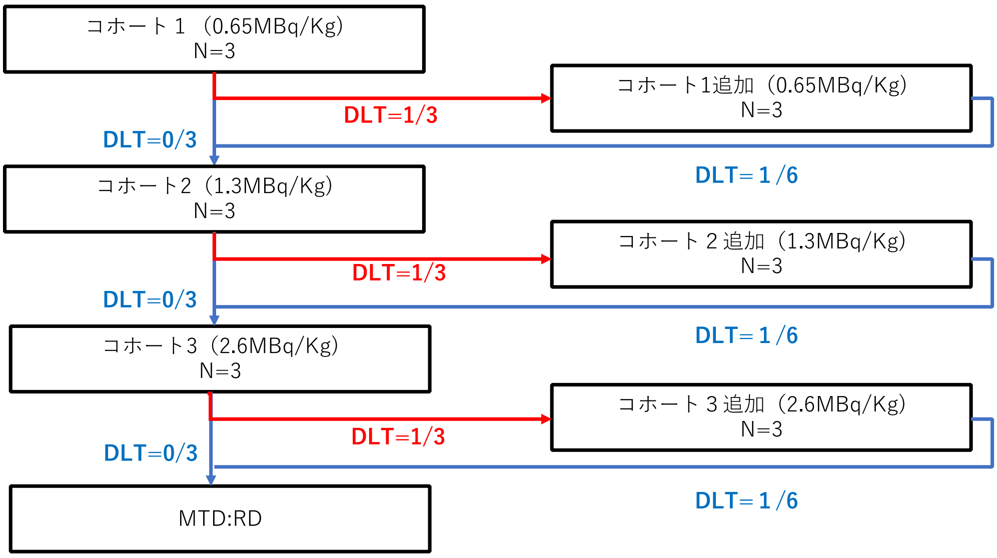

【設定根拠】

初回投与量：非臨床試験の結果において、マウスの 80MBq/kg 群について 50 匹中 3 匹に重篤な毒性が発生したため STD10 を 80MBq/kg とみなし、「「抗悪性腫瘍薬の臨床評価方法に関するガイドライン」の改定（令和 3 年）」より初回投与量はげっ歯類の STD10 の 1/10 のため、治験における初回投与量を 8MBq/kg（マウス）のヒト等価用量とする。

ヒト等価用量に換算した場合 0.65 MBq/kg となることから公比1：2:4 とし、用量レベル1：0.65MBq/kg、用量レベル2：1.3MBq/kg、用量レベル3：2.6MBq/kg とした。

：

3.4　用量制限毒性（Dose Limiting Toxicity：DLT）の定義

DLT の判定期間は治験薬投与から Day43 までとする。

DLT の判定期間が終了するまで次の被験者を登録しない。

DLT は CTCAE Grade4 の血液毒性および Grade3 以上の非血液毒性及び発熱性好中球減少症、赤血球輸血を要する貧血及び血小板輸血を要する血小板減少症とする。

ただし，以下に該当する有害事象を除く。

- 食欲不振又は疲労
- 経管栄養法又は TPN を要しない Grade 3 の悪心又は嘔吐，及び入院期間の延長を伴わない Grade 3 の下痢。ただし，添付文書用量で使用した標準的な制吐剤又は止瀉薬にて発現から 7 日以内に Grade 2 以下に管理可能な事象とする。
- Grade 3 の感染

なお、DLT は被験者ごとに治験責任医師と分担医師が協議の上、最終決定する。

【設定根拠】
マウスの拡張型単回投与毒性試験より、Day5 から Day13 に顕著な一般状態の悪化を示しており、安楽死及び死亡は Day8 と Day9 に認めている。血液検査では Day5 に白血球値が最低を示し、その後回復している。病理学的検討では Day35 において、Day5 および Day14 に認めた小腸粘膜の障害は Day35 では見られなかった。以上の所見からヒトにおいても DLT の判定については 35 日間で十分と考えたが、保守的に 42 日間観察することとした。

3.5　各コホートにおける DLT の評価手順

1)各コホートの 1 例目より、DLT が発現した時点で、効果安全性評価委員会（以下、委員会）にその内容を報告し、委員会を開催する。
2)委員会は、本治験の中止や中断等の措置の必要性について検討する。治験責任医師は委員会の検討結果を参考に、今後の措置を決定する。
3)各コホートにて 3 例目あるいは 6 例目の DLT 評価期間が終了した時点で、効果・安全性評価委員を開催し、本治験の中止や中断等の措置の必要性について検討する。その際、入手可能な全てのデータを確認し、治験責任医師は、効果・安全性評価委員の検討結果を参考に、次コホートへの移行の可否を決定する。

なお、DLT 発現後は、今後の措置が決定するまで新規被験者の登録及び投与期中の被験者の治験薬投与は中断する。

3.6　評価項目

3.6.1　主要評価項目

治験薬を単回投与時の至適用量決定のために用量制限毒性（Dose Limiting Toxicity：DLT）の発現の有無を主要評価項目とする。DLT は CTCAE Grade4 の血液毒性および Grade3 以上の非血液毒性等とする(詳細については、3.4 用量制限毒性（Dose Limiting Toxicity：DLT）の定義　参照)。

3.6.2　副次的評価項目

1)　放射能薬物動態（Radiopharmacokinetics：RPK）
患者に治験薬を投与後の血漿中濃度の推移を測定する。以下のスケジュールに従い採血を行い各コホートにおける薬物動態パラメータとして、C_{max}、AUC、$t_{1/2}$、V_{ss}、CL 等の幾何平均等を算出する。薬物動態解析の詳細は別途規定する薬物動態解析計画書に記す。

2) 尿中放射能排出率
全症例及び各コホートにおける投与後 24 時間までの放射能尿中排出率について時系列で算出する。

3) 尿中カテコラミン奏効率
スクリーニング時に尿中カテコラミン類尿（アドレナリン、ノルアドレナリン、メタネフリン、ノルメタネフリン）のいずれかが基準値上限の 3 倍以上であった患者を対象とし、基準値上限の 3 倍以上であったすべての尿中カテコラミン類について、最良総合効果が CR（効果判定の対象とされたすべての尿中カテコラミン類の値が基準値内まで減少）又は PR（効果判定の対象とされたすべての尿中カテコラミン類の値がベースライン時と比較して 50%を超えて減少）の場合に奏効と判定する

4) 奏効率（Overall Response Rate：ORR）
腫瘍に対して「Response evaluation criteria in solid tumours」（RECIST ver. 1.1）に従って判定した総合効果に基づく奏効率（ORR）を評価する。なお、判定に際して CR 及び PR の確定は行わない。

5) 無増悪生存期間（Progression free survival：PFS）
腫瘍に対して「Response evaluation criteria in solid tumours」（RECIST ver. 1.1）に基づく客観的主要評価を行い、登録日を起算日として死亡または増悪（臨床的な増悪を含む）のいずれか早い方までの期間。

6) ^{123}I-MIBG シンチグラフィによる腫瘍集積減少効果
画像評価の手順書に従い、総合判定効果に基づく、シンチグラフィ総合評価を行う。

7) ＱＯＬ
EORTC QLQ−C30、EQ−5D−5L を測定する。

3.7 治験実施予定期間

2022 年 4 月～2024 年 3 月

3.8 症例数

最大症例数として 18 例（設定根拠は「3.3.1 用法・用量の定義」参照）

4 前治療、併用薬剤・併用療法、後治療

4.1 前治療

本治験に参加する前に受けた PPGL に対する治療を前治療とする。

なお、本治験への登録に際し、PPGL に対する前治療による影響がないこと（選択基準に示した検査値の基準を満たしていること）を確認する。

4.2 併用薬剤・併用療法

症例登録日 30 日前から投与後 12 週までに使用したすべての併用薬剤（漢方薬、市販薬を含む）および同期間に実施したすべての併用療法を併用薬剤・併用療法とする。ただし、希釈用の生理食塩液等は除く。

4.2.1 併用禁止薬剤・併用禁止療法

同意取得時から PD や臨床的増悪が確認されるまでの期間は本治験の有効性および安全性の評価に影響を及ぼすと考えられる下記の薬剤および治療法の使用を禁止する。

1) 手術療法
2) 化学療法
3) 顆粒球コロニー刺激因子（G-CSF）
   ※使用歴がある場合には最後の使用から 8 日以上経過していること
4) ホルモン療法
5) 抗体療法
6) 放射線療法(核医学治療を含む)
7) その他の抗腫瘍療法（いわゆる免疫療法を含む）
8) 副腎皮質ステロイド剤の継続的な全身投与（プレドニン換算で 10 mg/日を超えるもの）
9) 免疫抑制剤あるいは免疫賦活剤の全身投与
10) 抗腫瘍効果を目的とした健康食品（アガリスク等）等の摂取
11) 治験薬投与部位への副腎皮質ステロイド剤の局所使用
12) 抗不整脈薬（心室性不整脈）
13) 交感神経遮断薬（αβ遮断薬、α遮断薬、アドレナリン作動性神経遮断薬）
14) カルシウム拮抗薬：アムロジピン、ジルチアゼム等
15) 交感神経作動薬（変力性、血管収縮性、β_2 作動性）
16) アドレナリン受容体作動薬
17) 抗うつ薬
18) 抗精神病薬（神経遮断薬）
19) 中枢神経刺激薬

20) 治験薬投与前の輸血
　※輸血歴がある場合には、最後の輸血から 29 日以上経過していること。

4.2.2　併用注意薬剤

特に規定を設けないが、新たな併用薬剤が投与された場合は、その具体的な薬剤名、投与日と投与理由について症例報告書に記載する。

4.2.3　併用可能薬剤および推奨される支持療法

登録後、甲状腺ブロックとして、治験薬投与の 1〜3 日前（少なくとも 24 時間より前）から投与後 7 日までヨウ化カリウム 50mg/日の経口投与を行う。

治験薬投与直前に、嘔吐の予防のため5-HT_3 受容体拮抗剤を投与する

4.2.4　後治療

PD や臨床的増悪が確認された後については、後治療について制限しない。

5　被験者に対する同意・説明文書の作成と改訂

説明文書・同意文書は、GCP 省令および業務手順書に従って治験責任医師が作成する。ただし、被験者を意図的に誘導するような記載をしてはならない。また、作成した説明文書・同意文書は治験開始前に治験審査委員会と実施医療機関の長の承認を得る。

治験開始後に治験責任医師が被験者の同意に影響を与える新たな知見を得、説明文書・同意文書の改訂が必要と判断した場合には、それを改訂し、予め治験審査委員会と医療機関の長の承認を得る。被験者の同意に影響を与える新たな知見とは、例えば当該治験薬等に関連する新たな有害事象の情報、あるいは対象疾患に関わる新治療法等の開発に関する情報などを指す。

6　対象被験者

6.1　対象被験者

褐色細胞腫/パラガングリオーマ患者で、「6.2 選択基準」を全て満たし、かつ「6.3 除外基準」に抵触しない患者を対象とする。ただし、性別は問わない。

6.2　選択基準

以下のすべての条件を満たすものとする。

1) 本人より文書による同意取得が可能な患者
2) 褐色細胞腫、パラガングリオーマ、悪性褐色細胞腫、悪性パラガングリオーマのいずれかと組織学的または臨床学的に診断されている患者

※診断については「褐色細胞腫・パラガングリオーマ診療ガイドライン 2018」に従うこととする。

3) 以下に定義する褐色細胞腫と診断されている患者（下記①～③のいずれかを満たし、かつ外科的切除や根治的放射線外照射が不可能なものを難治性褐色細胞腫と定義する）
   ① 初発時に原発巣の高度な局所進展を有する褐色細胞腫/パラガングリオーマ
   ② 初発時に遠隔転移を有する悪性褐色細胞腫/悪性パラガングリオーマ
   ③ 外科的切除を行うも局所再発または遠隔転移再発を来した褐色細胞腫/パラガングリオーマ
4) 同意取得時の年齢が 20 歳以上の患者であること
5) ECOG Performance status（PS）が 0～2 の患者であること
6) スクリーニング時に実施された CT 画像で確認された標的病変の 1 つ以上で、スクリーニング時に実施された ^{123}I-MIBG シンチグラフィで集積陽性が確認されている患者
7) スクリーニング時の検査値が、以下の基準をすべて満たす患者であること
   ① 骨髄機能
      (1) 顆粒球コロニー刺激因子（G-CSF）製剤非投与下で白血球数≧3,000 /μL
      (2) 非輸血下でヘモグロビン≧9.0 g/dL
      (3) 非輸血下で血小板数≧$10×10^4$/mm^3（μL）
   ② 腎機能
      (1) 推定糸球体濾過量（eGFR）≧30mL/min/1.73m^2
   ③ 肝機能（JSCC 標準化対応法）
      (1) AST≦90 U/L
      (2) ALT≦126 U/L（男性）、ALT≦69 U/L（女性）
      (3) LDH＜666 U/L
   ④ 心機能
      (1) NYHA Functional class：Ⅰ以下
   ⑤ 糖尿病・内分泌疾患
      (1) HbA1c＜8.0%（NGSP 値）
   ⑥ 呼吸状態
      (1) 大気中における血中酸素飽和濃度（SpO2）≧96%
8) 3 ヶ月以上の生存が期待できる患者
9) 核医学治療病室内隔離期間中、摂食、排泄および睡眠の自立が見込める患者
10) 標準的な治療法（CVD 療法または ^{131}I-MIBG 治療）で効果がなかった、または他に適切な治療法がない患者

【設定根拠】

1) GCP省令を遵守し、患者が自由意志により治験参加の判断をすることを可能とするため。

2) ,3) 対象疾患が褐色細胞腫/パラガングリオーマであるため。（試験対象疾患を明確にするため）

4) 本人からの同意を前提とするため、20歳以上とした。

5)〜10) 試験成績に影響を及ぼす可能性があるため。

6.3 除外基準

以下の項目のいずれかに該当する患者は除外する。

1) 活動性の重複がんを有する患者
   ここでの活動性の重複がんとは、同時性重複がん/多発がんおよび無病期間が5年以内の異時性重複がん/多発がんとする。ただし、局所治療により治癒と判断され、治癒後の無病期間が1年を超えた上皮内がんや粘膜内がん相当の病変は、活動性の重複がん/多発がんに含めない。また、以下に示す家族性褐色細胞腫として合併するがんは活動性の重複がん/多発がんに含めない。
   (1) 多発性内分泌腫瘍2型（MEN2）における甲状腺髄様がん
   (2) von Hippel Lindau病（VHL）における網膜血管芽腫
   (3) 神経線維腫症1型（NF1）における神経線維腫
2) MABGの集積を抑制する薬剤を所定の期間、中止することができない患者
3) 所定の期間α-メチルパラチロシンの服用を中止することができない患者
4) 登録前8週間以内に手術、CVD療法、肝転移に対するカテーテル肝動脈塞栓療法、放射線療法を受けたことがある患者
5) 登録前12週間以内にMIBG治療を受けたことのある患者
6) 前治療時において治療時あるいは治療後に治療と因果関係を否定できないグレード2以上の非血液毒性の出現を認め、治験期間中に加療を要する患者
7) 以下のいずれかの感染症がありかつ治験期間中に加療を要する患者
   (1) B型肝炎ウイルス感染症
   (2) C型肝炎ウイルス感染症
   (3) HIV感染症
   (4) その他全身治療を要する感染症
8) 副腎皮質ホルモン（プレドニンもしくはプレドニゾロン換算量10 mg以上/日）またはその他の免疫抑制剤の持続的な全身投与を必要とする疾患がありかつ治験期間中にも加療を要する患者
9) コントロール困難なカテコラミン発作の既往がある患者

10) 致死性不整脈もしくは心停止の既往のある患者
11) コントロール不良の症候性不整脈、甲状腺機能異常、呼吸器疾患、胸腹水貯留のある患者
12) 冠動脈疾患、アミオダロンによる加療を要する不整脈、重症心弁膜症、大動脈疾患、出血傾向がある疾患・状態がある患者
13) 妊娠中(医師の問診等により妊娠している可能性があると判断された場合にも、当該患者を試験から除外)、産後 28 日以内または授乳中（授乳中の女性が授乳を中断しても不可）の患者
14) 妊娠する可能性がある女性患者あるいはパートナーが妊娠する可能性がある男性患者で投薬治療後 6 ヶ月間避妊することに合意できない患者（避妊法としては、ラテックス製コンドーム（男性が使用）、各種ホルモン避妊薬（経口避妊薬等）、子宮内避妊具（IUD）（プロゲステロン無添加 T 型）のいずれか 2 種類以上を併用するか、卵管結紮及び精管切除）
15) 同意日の前 3 か月以内に他の治験に参加している患者
16) その他、治験責任医師または治験分担医師が本治験の対象として適切ではないと判断した患者

【設定根拠】
1)～12) 試験成績を適切に評価するため。
13), 14) 本剤の生殖機能に対する危険性および本剤の乳汁移行性および胎児毒性が検討されていないため。
15), 16) 試験成績を適切に評価するため。

7　　同意取得の時期と方法

下記の手順により被験者の自由意志による同意を文書により得る。

7.1　　登録前

1) 治験責任（分担）医師は、登録可能と考えられる被験者候補を治験に参加させる（治験のために実施するあらゆる医療行為を行う）前に、「5 被験者に対する同意・説明文書の作成と改訂」に定める同意文書およびその他の説明文書を用いて十分に説明を行う。また、必要な場合には、治験協力者も補足的な説明を行うことができる。
2) 治験責任（分担）医師は同意を得る前に、被験者候補が質問をする機会と、治験に参加するか否かを判断するのに十分な時間を与える。
3) 治験責任（分担）医師および治験協力者は、被験者候補からの全ての質問に対して、被験者候補が満足するような回答を示す。

4) 被験者候補が治験に参加することを同意した場合、説明を行った治験責任（分担）医師並びに被験者は、同意文書に署名し、日付を記載する。なお、治験協力者が補足的に説明を行った場合は、その治験協力者も署名し、その日付を記載する。
5) 治験責任（分担）医師は、被験者が治験に参加する前に、同意文書の写しおよび説明文書を被験者に手渡し、治験責任医師は治験実施医療機関においてその原本を保存する。

7.2 被験者の意思に影響を与える情報入手時

治験責任（分担）医師は、治験の参加継続に関して被験者の意思に影響を与える可能性のある情報を入手した場合は、その内容を速やかに治験に参加中の被験者に伝えるとともに治験継続の意思を確認し、記録に残す。

7.3 同意文書およびその他の説明文書の改訂時

1) 治験責任医師が同意文書およびその他の説明文書を改訂する場合は、その改訂内容を速やかに治験に参加中の被験者に伝えるとともに治験継続の意思を確認し、記録に残す。
2) 治験審査委員会が承認した改訂後の同意文書およびその他の説明文書を用いて「7.1 登録前」に従って再度、被験者の同意を得る。
なお、同意文書およびその他の説明文書改訂（軽微なものを除く）中は、新規の被験者登録に対する同意取得を一時中断する。

7.4 被験者の他科および他院受診に際して

治験責任（分担）医師は、同意を取得した被験者から、他科または他院を受診しているかを確認する。受診している場合は、被験者の同意を得た上で受診中の他科または他院の担当医師に対し、被験者が本治験に参加する旨を通知しなければならない。

なお、被験者が遠方から受診している場合は、被験者の合意の上で事前に近隣でフォローアップ可能な、かかりつけ医を決め、治験責任（分担）医師が当該医師に事前に連絡をとり、当該被験者の治験に関する情報を共有する。治験期間中、かかりつけ医の診察を受けた場合は、治験責任（分担）医師は十分な情報収集を行う。

8 被験者の登録

8.1 症例登録の手順

1) 治験責任（分担）医師および治験協力者は、適格と判断され同意が得られた被験者の情報を「症例登録票」に記入する。

2) 治験責任医師は、「症例登録票」の記載内容に基づいて適格性を判定し、適格と判定された場合、症例登録を行う。
3) 治験責任(分担)医師あるいは治験協力者は、症例登録の完了を確認後、速やかに本治験で規定された治療を開始する。不適格の場合は、当該被験者本人に本治験への登録が不可である旨を説明する。

8.2 追加登録

各コホートにおいて3例から6例の症例登録を予定するが、登録した症例が DLT 評価期間を終了せずに治験を中止した場合(DLT が発現した場合を除く)は、必要に応じて追加の被験者を登録する。

9 調査・観察・検査、評価項目および実施時期

9.1 調査・観察検査および実施時期

治験期間中は「表 9-1 調査・観察・検査項目一覧」に従って、調査・観察・検査を行い、実施日ならびに結果を症例報告書に記載する。なお、治験実施計画書にて規定した以外の日に実施した検査結果については、有害事象情報に関連する調査・観察・検査の実施日および検査結果を症例報告書に記載すること。

なお、症例登録前に実施する調査・観察・検査は、同意取得後に実施することとするが、規定の観察時期に同意取得前(同意取得前 30 日以内)の調査・観察・検査結果がある場合は、これに替えることができる。

表 9-1 調査･観察･検査項目一覧

調査/検査項目	投与前				投与日			投与後									
	同意	スクリーニング期間	被験者登録	7日以内	投与前	投与	投与終了後	投与翌日	2日後	3日後	7日後	2週	4週	6週	8週	12週	治験中止時*14
		Day-42 ～-14	～Day-14	Day-7 ～-1	Day1			Day2	Day3	Day4	Day8	Day15	Day29 ±7	Day43 ±7	Day57 ±7	Day85 ±7	±14
		入院			入院									入院		入院	入院
文書同意取得	●																
登録			●*12														
^{211}At-MABG投与						●											
ヨウ化カリウム投与				●*13	●*13			●*13									
5-HT$_3$受容体拮抗剤投与					●												
被験者背景の調査		●															
併用薬/併用療法の調査			●→													→	●
**全身状態**																	
ECOG Performance Status		●		●				●→				●	●	●	●	●	●
身長		●															
体重		●		●								●	●	●	●	●	●
診察所見		●		●	●		投与直後、1,2,3,4,6時間後	●→				●	●	●	●	●	●
バイタルサイン*1		●		●	●		投与直後、1,2,3,4,6時間後	●→				●	●	●	●	●	●
血中酸素飽和度(SpO$_2$)		●		●	●		投与直後、1,2,3,4,6時間後	●→				●	●	●	●	●	●
**血液生化学検査**																	
血液学的検査*2		●		●	●		1,3,6時間後	●		●	●	●	●	●	●	●	●
血液生化学*3		●		●	●		1,3,6時間後	●		●	●	●		●		●	●
尿*4		●		●										●		●	●
感染症検査*5		●															
心電図検査（12誘導）		●			●		投与後５分後、1,3,６時間後	●		●	●	●		●		●	●
心臓超音波検査		●												●		●	●
尿中カテコラミン類検査*6		●												●		●	●
^{123}I-MIBGシンチグラフィ*7		●												●		●	●
^{123}I-MIBG集積の確認*8		●															
QOL調査*9		●												●		●	●
**放射能薬物動態**																	
血中放射能濃度測定					●		投与後5分、15分、1,3,６時間後	24時間後	48時間後	72時間後							
尿中放射能濃度測定					●		投与後0-3,3-6,6-12時間	12-24時間	24-48時間	48-72時間							
CT検査*10		●												●		●	●
妊娠検査（hCG）*11		●															
有害事象			●→													→	●

*1 収縮期血圧、拡張期血圧、脈拍数および体温を測定する。血圧、脈拍数は原則座位で、少なくとも5分間安静にした後で測定する。

*2 赤血球数、白血球数、白血球分画（好中球、好酸球、好塩基球、リンパ球、単球）ヘモグロビン、ヘマトクリット、血小板数

*3 AST、ALT、ALP、γ-GTP、LDH、C 反応性蛋白(CRP)、総タンパク、アルブミン、総ビリルビン、総コレステロール、BUN、尿酸、ナトリウム、カリウム、クロル、カルシウム、推算糸球体濾過(e-GFR)、HbA1c、尿性ナトリウム利尿ペプチド、遊離トリヨードサイロニン(FT3)、遊離サイロキシン(FT4)、甲状腺刺激ホルモン(TSH)

*4 タンパク、糖、ウロビリノーゲン、潜血

*5 HBs 抗原、HBs 抗体、HBc 抗体、HCV 抗体(必要に応じてウイルス量測定)、HIV 抗体。治療開始後は、HBV に関しては「免疫抑制・化学療法により発症する B 型肝炎対策ガイドライン」に従いウイルス量のモニタリングを行う。

*6 アドレナリン、ノルアドレナリン、メタネフリン、ノルメタネフリン:24 時間蓄尿を行う。

*7 前面および後面の全身プンマー撮影、並びに SPECT 撮影を実施する。

*8 スクリーニング中の I-123 MIBG シンチグラフィにより、スクリーニング時の CT 画像で確認された標的病変の 1 つ以上で I-123 MIBG の集積を確認する。

*9 調査は、画像検査の結果を被検者に説明する前に、EORTIC-QLQ-C30 および EQ-5D-5L による調査票で実施する。

*10 原則単純 CT により撮影する。

*11 妊娠する可能性がある(外科的処置により不妊の状態にない、閉経前である、別の医学的理由により 12 ヶ月以連続で無月経の状態にない)女性被験者のみ。

*12 被験者の登録は被験薬の発注を兼ねる。

*13 甲状腺ブロックとして、211At-MABG 投与の1～3 日前(少なくとも 24 時間より早く)から投与後 7 日目までヨウ化カリウム 50mg/日の投与を行う。

*14 中止が決定された日±14 日に、被験者の臨床状態を考慮した上で可能な限り実施する。

9.2　　同意の取得

「7 同意取得の時期と方法」に従って、同意を取得する。遅くとも症例登録前検査実施までに文書による同意を取得する。被験者の同意が得られた場合においては、通常診療の一環として実施された調査・観察・検査を治験実施計画書に規定された調査・観察・検査として使用することもできる。同意文書に署名することにより、被験者はこれに同意するものとする。

9.3　　被験者識別コードの割り当て

治験責任（分担）医師は、同意を取得した全ての被験者に、被験者識別コードを付与する。

9.4　　入院及び核医学治療室への入室

スクリーニングは入院で行う。登録後は、投与日前より入院し、投与後は核医学治療室に入院する。

核医学治療室退出後も Day15 までは原則、入院にて経過観察を行う。退院については医師が診察した上で①血液検査に問題がなく治験薬投与後に発生した因果関係の否定できない有害事象が Grade2 以下まで改善していること②被験者が家族と同居している、または、1 日 1 回以上、家族等が自宅に訪問することができる③被験者と直接電話あるいは電子メールにて連絡することが可能であることの全てを条件とする。また、退院時に自宅近隣医療施設へ電話で退院の報告を行い、緊急時の連絡先を含めた診療情報提供書を送付する。Day43、Day85 及び中止時の検査は入院にて行う。

9.5　　治験期間中に実施する調査・観察・検査

9.5.1　被験者背景

1)　調査・観察・検査項目

(1)　同意取得日

同意取得日を症例報告書に記載する。

再同意を取得した場合は、同意取得日および再同意の理由を症例報告書に記載する。

(2)　症例番号、症例登録日

症例登録確認票に記載の症例番号、症例登録日、登録に至らなかった場合は被験者識別コード、登録不可の旨を症例報告書に記載する。

(3)　性別、生年月日

被験者の性別、生年月日を調査し、症例報告書に記載する。

(4) 診断名

　診断名を症例報告書に記載する。

(5) 初回診断日

　褐色細胞腫/パラガングリオーマの初回診断日を症例報告書に記載する。

(6) 前治療歴

　褐色細胞腫/パラガングリオーマに対する前治療の詳細、前治療期間を調査し、症例報告書に記載する。なお、同意取得日直近の前治療については、結果も記載する。

(7) 既往歴

治験期間中の評価に影響を及ぼさないと考えられる疾患を除き、症例登録日より過去 3 年間の疾患名（症状名）および罹病期間を調査し、症例報告書に記載する。

ただし、悪性腫瘍の場合、期間は問わない。

(8) 合併症

　症例登録時に症状の有無を問わず、罹病中の疾患または合併症の疾患名（症状名）、併用薬剤・併用療法の有無および罹病期間を調査し、症例報告書に記載する。ただし、治験期間中の評価に影響を及ぼさないと考えられる疾患は除く。

2) 調査・観察・検査時期

・ 症例登録前：(1)～(8)

9.5.2 **併用薬剤・併用療法・輸血**

1) 調査・観察・検査項目

　症例登録日 30 日前から投与後 12 週までに使用した全ての併用薬剤については、薬剤名、 用法、用量、投与経路、使用期間および使用理由を調査し、症例報告書に記載する。

　症例登録日 30 日前から投与後 12 週までに実施した全ての併用療法については、療法名、頻度、実施期間および実施理由を調査し、症例報告書に記載する。

　症例登録日 30 日前から投与後 12 週までに実施した全ての輸血については、輸血製剤の種類（赤血球濃厚液輸血または血小板濃厚液輸血）、投与単位数、実施日、使用理由を症例報告書に記載する。

2) 調査・観察・検査時期

- 症例登録日から投与後 12 週まで
- 中止時

9.5.3　全身状態

1)　調査・検査項目

PS（付録 1）、身長、体重、自・他覚所見およびバイタルサイン（収縮期および拡張期の血圧、脈拍数、体温）を調査、測定し、検査日とともに症例報告書に記載する。なお、血圧測定については、治験期間中を通して原則座位に統一する。

2)　調査・検査時期

(1)　PS：

- 症例登録前
- 入院日（Day-7～Day-1）
- Day2～Day15、Day29、Day43、Day57、Day85
- 中止時

(2)　身長（cm）：

- 症例登録前

(3)　体重（kg）：

- 症例登録前
- 入院日（Day-7～Day-1）
- Day15、Day29、Day43、Day57、Day85
- 中止時

(4)　診察所見：

治験責任医師もしくは分担医師は診察を行い、以下の身体機能／器官を評価する。

一般的所見、皮膚、頭部、眼、耳、鼻、口、咽喉、肺、心血管、腹部、尿生殖器（随時）、
リンパ、筋骨格／四肢、神経

- 症例登録前
- 入院日（Day-7～Day-1）
- 投与前
- 投与終了直後、1、2、3、4、6 時間後
- Day2～Day15、Day29、Day43、Day57、Day85
- 中止時

(5)　バイタルサイン（収縮期及び拡張期血圧、脈拍、体温）：

- 症例登録前
- 入院日（Day-7～Day-1）
- 投与前
- 投与終了直後、1、2、3、4、6 時間後
- Day2～Day15、Day29、Day43、Day57、Day85

- 中止時

(6) 血中酸素飽和度（SpO_2）

- 症例登録前
- 入院日（Day-7～Day-1）
- 投与前
- 投与終了直後、1、2、3、4、6 時間後
- Day2～Day15、Day29、Day43、Day57、Day85
- 中止時

9.5.4 臨床検査

1) 血液学的検査

(1) 検査項目

赤血球数、白血球数、白血球分画（好中球数、好酸球、好塩基球、リンパ球数、単球）、ヘモグロビン値、ヘマトクリット値、血小板数を検査し、異常変動の有無について検査日とともに症例報告書に記載する。

(2) 検査時期

- 症例登録前
- 入院日（Day-7～Day-1）
- 投与前、投与 1、3、6 時間後
- Day2、Day4、Day8、Day15、Day29、Day43、Day57、Day85
- 中止時

2) 血液生化学検査

(1) 検査項目

AST (GOT)、ALT (GPT) 、ALP 、γ-GTP、LDH、CRP、 総蛋白、アルブミン、総ビリルビン、総コレステロール、BUN、尿酸、Na、K、Cl、Ca、e-GFR、HbA1c、BNP、FT3、FT4、TSH を検査し、異常変動の有無について検査日とともに症例報告書に記載する。

(2) 検査時期

- 症例登録前
- 入院日（Day-7～Day-1）
- 投与前、投与 1、3、6 時間後
- Day2、Day4、Day8、Day15、Day43、Day85
- 中止時

3) 尿検査

(1) 検査項目

蛋白、糖、ウロビリノーゲン、潜血を検査し、異常変動の有無について検査日とともに症例報告書に記載する。

(2) 検査時期

- 症例登録前
- 入院日（Day-7～Day-1）
- Day43、Day85
- 中止時

9.5.5 その他の検査

1) 感染症検査

(1) 検査項目

HBs 抗原、HBs 抗体、HBc 抗体、HCV 抗体（必要に応じてウイルス量測定）、HIV 抗体の検査を実施し、検査日とともに症例報告書に記載する。治療開始後は、HBV に関しては「免疫抑制・化学療法により発症する B 型肝炎対策ガイドライン」に従いウイルス量のモニタリングを行う。

(2) 検査時期

・ 症例登録前

2) 心電図検査（12 誘導）

(1) 検査項目

12 誘導心電図（3 回測定）を測定する。心電図（3 回測定）は、5 分間、仰臥位又は半仰臥位で安静にさせた後、数分間隔で連続して測定する。標準心電図パラメータとして RR、PR、QT 間隔及び QRS 間隔を測定する。異常の有無、所見、臨床上の意義を検査、評価し、検査日とともに症例報告書に記載する。

(2) 検査時期

- 症例登録前
- 投与前、投与後 5 分(±1 分)、投与 1（±10 分）、3（±10 分）、6時間後（±10 分）
- Day2、Day4、Day8、Day15、Day43、Day85
- 中止時

3) 心臓超音波検査

(1) 検査項目

異常の有無、所見、臨床上の意義を検査、評価し、検査日とともに症例報告書に記載する。

(2) 検査時期
- 症例登録前
- Day43、Day85
- 中止時

4) 尿中カテコラミン類検査

(1) 検査項目

　24 時間蓄尿を実施し、尿中アドレナリン、ノルアドレナリン、メタネフリン、ノルメタネフリンを測定し、検査日とともに症例報告書に記載する。

(2) 検査時期
- 症例登録前
- Day43、Day85
- 中止時

5) ^{123}I-MIBG シンチグラフィ

(1) 検査項目

^{123}I-MIBG を投与し、全身シンチグラフィによる病巣への MIBG 集積を評価し、検査日とともに症例報告書に記載する。^{123}I-MIBG シンチグラフィ集積の評価方法については、別途手順書に記載する。

(2) 検査時期
- 症例登録前
- Day43、Day85
- 中止時

6) QOL 調査

(1) 検査項目

EORTC QLQ-C30 および EQ-5D-5L 質問票を使用して PRO を評価し、検査日とともに症例報告書に記載する。

(2) 検査時期
- 症例登録前
- Day43、Day85
- 中止時

7) 放射能薬物動態

(1) ^{211}At-MABG を単回静脈内投与した後の血中放射能濃度採血を実施し、採取日、採血時間を症例報告書に記載する。薬物動態評価用採血の指定時

刻と同じ時刻にバイタルサインの測定が予定されている場合は，放射能薬物動態評価用採血の前に、バイタルサインの測定を実施する。採取した検体については先端臨床研究センターに送付して放射能値を解析する。検体の採取、取扱い、輸送、解析の詳細については別途手順書に記載する。

(2) 検査時期

- 投与前（-30 分）、投与 5 分（±1 分）、投与 15 分（±5 分）、1 時間(±5 分)、3 時間(±10 分)、6 時間(±10 分)、24 時間(±60 分)、48(±60 分)時間、72 時間（±60 分）後

8) 放射能薬物動態(尿中排泄)

(1) ^{211}At-MABG を単回静脈内投与した後の尿を一定時間ごと蓄尿し、採取日、採取時間を症例報告書に記載する。採取した検体については先端臨床研究センターに送付して放射能値を解析する。検体の採取、取扱い、輸送、解析の詳細については別途手順書に記載する。

(2) 検査時期

- 投与前、投与後 0〜3 時間、3〜6 時間、6〜12 時間、12〜24 時間、24〜48 時間、48〜72 時間

9.5.6 **腫瘍関連検査・観察項目**

1) 腫瘍評価（原則、単純 CT を用いる。但し、治験責任医師等の判断で造影剤を使用する場合は添付文書に従うこと）検査

(1) 検査項目

標的病変および非標的病変の評価を登録時と同じ検査法にて行う。胸部、腹部の CT を行い、腫瘍径を測定する。

投与後は、奏効あるいは増悪の判定を RECIST 基準 version1.1[23]（付録 2）に従って分類し、腫瘍効果の判定は治験責任医師が判定する。

全ての患者のファイルおよび放射線画像を症例報告書と原資料との確認のために利用する。

(2) 検査時期

- 症例登録前
- Day43、Day85
- 中止時
- 被験者の臨床症状に応じて、臨床的に必要と判断された場合には、適宜胸部 CT 検査を実施する（RECIST に基づく評価も行う）。

9.5.7 妊娠検査：妊娠の可能性を有する女性のみ

以下の 2 項目のうち 1 項目以上に該当する女性は妊娠の可能性はないと判断し、来院時における妊娠検査は必要としない。

- 不妊手術の既往がある（子宮摘出、両側卵管結紮または両側卵巣摘出）
- 最終月経より 1 年以上経過している

但し、薬剤等による無月経状態と考えられる被験者は妊娠検査を行うこと。

(1) 検査項目

血液中ヒト絨毛性ゴナドトロピン（hCG）濃度測定を実施し、検査実施の有無、検査日、検査結果を症例報告書に記載する。なお、女性被験者において妊娠検査を実施しない場合には、未実施の理由を症例報告書に記載する。

(2) 検査時期

- 症例登録前

9.5.8 本剤の投与状況

投与の有無、投与日、投与時刻について、症例報告書に記載する。

9.5.8.1 ヨウ化カリウムおよび 5-HT$_3$ 受容体拮抗剤の投与状況

投与の有無、投与日、投与時刻について、症例報告書に記載する。

9.5.9 完了、中止

治験の完了または中止、中止理由について、症例報告書に記載する。

9.5.10 入退院の状況

同意取得日から投与後 12 週または治験中止までに、規定された検査・観察のための入院以外の入院が必要となった場合、必要とした期間およびその理由について、症例報告書に記載する。

9.6 評価

9.6.1 有効性の評価

RECIST 基準 version1.1[23)]（付録 2）に従って治験責任医師または治験分担医師により、有効性の評価を行う。

1) 奏功率（ORR）

最良総合効果で CR または PR を示した被験者の割合。

2) 無増悪生存期間（PFS）
登録日を起算日として、増悪（再発/再燃を含む）または理由を問わない死亡までの期間とする。
3) 尿中カテコラミン類奏効率
スクリーニング時に尿中カテコラミン類尿（アドレナリン、ノルアドレナリン、メタネフリン、ノルメタネフリン）のいずれかが基準値上限の 3 倍以上であった患者を対象とし、基準値上限の 3 倍以上であったすべての尿中カテコラミン類について、最良総合効果が CR（効果判定の対象とされたすべての尿中カテコラミン類の値が基準値内まで減少）又は PR（効果判定の対象とされたすべての尿中カテコラミン類の値がベースライン時と比較して 50%を超えて減少）の場合に奏効と判定する
4) I-123 MIBG シンチグラフィによる腫瘍集積減少効果
画像評価の手順書に従い、総合判定効果に基づく、シンチグラフィ総合評価を行う。
5) QOL 評価
EORTC QLQ-C30 および EQ-5D-5L 質問票を使用して PRO を評価する。

9.6.2 安全性の評価

本剤が投与された被験者に生じたあらゆる好ましくない医療上の出来事（臨床検査値の異常変動を含む）を有害事象として取り扱い、安全性の評価を行う。

なお、複数の症状・徴候等が 1 つの診断名（疾患）として説明できる場合には、付随する個別の症状・徴候は記載せず、原則として該当する診断名を有害事象名とする。

＜例＞鼻水、咳、のどの痛み：左記 3 症状が「急性上気道炎」による一連の症状として説明できる場合→「急性上気道炎」

9.6.3 有害事象

治験責任医師または治験分担医師は、被験者の来院日（治験スケジュールに規定されているか否かは問わない）に、被験者の自発的な症状の訴えや問診、診察により、有害事象に関するデータを収集する。治験責任医師または治験分担医師は、収集した自覚症状もしくは他覚症状について医学的見地より有害事象に該当すると判断した場合は「11.2 有害事象の記録」に従って評価する。

9.6.4 臨床検査値の推移

本剤投与開始から投与後 12 週までに生じた臨床的に考慮すべき検査値または検査値の変動があった項目について、「11 被験者の安全性の確保」に従って評価する。

【設定根拠】

一般的な安全性評価項目として、本項目を設定した。

10 中止・終了基準

以下のいずれかに該当した場合、治験責任（分担）医師は速やかにその旨を被験者に通知し、直ちに治験薬投与を中止し、必要に応じて適切な処置を行う。また、有害事象発生時は被験者の安全を確保するための検査等を実施する。

1) 被験者が治験への参加の同意を撤回した場合。
2) 疾患の進行が認められた場合（画像診断の増悪であるのか、臨床的増悪であるのか等、具体的に疾患の進行による中止・終了の理由を症例報告書に記載する。なお、進行が認められた場合において、治験継続を正当化できる臨床的ベネフィットを示す合理的理由がある場合を除く。その場合には、被験者の安全性確保に留意すること。）。
3) 治験中止を要する全身状態の悪化を認めた場合。
4) 重篤な有害事象を認め、治験責任（分担）医師が治験薬の中止が望ましいと判断した場合。
5) 妊娠が判明した場合、避妊をしないあるいは不妊治療を開始する場合。
6) 追跡不能または来院しない場合。
7) 重大な治験実施計画書違反が明らかになった場合。
8) その他、治験責任（分担）医師が本治験の継続が不適切であると判断した場合。
9) 治験責任医師が治験を中止した場合。

10.1 中止・終了手順

10.1.1 被験者への対応

1) 治験責任医師または治験分担医師は治験期間中に当該被験者における治験を中止・終了する場合、当該被験者に速やかにその旨を説明し、必要な観察、検査および評価を行う。
2) 治験責任医師または治験分担医師は、症状の悪化および有害事象の発現等、被験者の安全性上の問題により治験を中止・終了した場合には、被験者に対し適切な処置を施す。
3) 治験を中止・終了した理由を症例報告書に記載する。

10.1.2 治験期間中に来院しなくなった被験者に対する調査

治験期間中に来院しなくなった被験者については追跡調査を行う。追跡調査は被験者に対する電話、ファクシミリ、書簡、訪問等の手段を用いて、来院しなくなった理由、有害事象の有無等について可能な限り調査し、結果を症例報告書に記載する。

10.2 被験者毎の完了および中止

10.2.1 完了例

治験薬の投与および投与後 12 週の観察が終了した症例を完了例として取り扱う。

10.2.2 中止例

「10 中止・終了基準」に合致し、Day1 から Day85 までの治療期間を満了できなかった症例を中止例として取り扱う。

11 被験者の安全性の確保

11.1 有害事象に関する定義

11.1.1 有害事象（AE）

有害事象（AE）とは、本剤との因果関係の有無に関わらず被験者に起こるあらゆる好ましくない医療上の出来事であり、意図しない徴候、臨床検査値の臨床的に有意な変動（表 11-1「CTCAE v5.0 日本語訳 JCOG/JSCO 版」に記載のない項目の重症度判定基準参照）、疾病、症状、合併症の悪化を含む。

有害事象については、同意取得から投与後 12 週までの間に新たに発現した有害事象を症例報告書に記録する。また、中止時においても症例報告書に記載する。PPGL の悪化は、有効性評価のもとで疾患進行として取り扱うこととし、有害事象としない。

全ての有害事象については、ベースラインの状態（Grade）に回復するまで、または回復が認められそうもない場合には症状が臨床的に安定するまで、あるいは当該被験者の追跡調査ができなくなるまで追跡調査を行う。

検査あるいは手術等の処置を講じた場合、処置自体は有害事象とせず、処置をするに至った原因の疾患を有害事象とする。被験者の死亡については、被験者が死亡するに至った原因の疾患を有害事象とする。

本剤を投与したにも関わらず、被験者の状態が無治療時と変わらず、本剤に期待する効果が見られない場合は有害事象としない。

本治験では、有害事象の評価基準として「有害事象共通用語規準 v5.0（CTCAE v5.0 日本語訳 JCOG/JSCO 版）」を用い、その規準に記載されている項目については、その規準における重症度が悪化した場合を、また、規準が記載されていない項目

については、表 11 -1「CTCAE v5.0 日本語訳 JCOG/JSCO 版」に記載のない項目の重症度判定基準 における重症度が、それぞれ悪化した場合を有害事象として取り扱い、症例報告書に記載する。

表 11-1「CTCAE v5.0 日本語訳 JCOG/JSCO 版」に記載のない項目の重症度判定基準

重症度	判定基準
グレード 1	軽度の有害事象<br>軽度；治療を要さない；症状がない画像所見異常／検査値異常。
グレード 2	中等度の有害事象<br>最低限の治療／局所的治療／非侵襲的治療を要する。
グレード 3	高度の有害事象<br>入院や侵襲的治療／インターベンショナルラジオロジー／輸血／治療的内視鏡／手術などを要する顕著な症状を有する。
グレード 4	生命を脅かす、または活動不能/動作不能となる有害事象<br>急性で生命を脅かす代謝性／心血管系の合併症など。集中治療や緊急処置（緊急インターベンショナルラジオロジー／治療的内視鏡／手術など）を要する。
グレード 5	有害事象による死亡

【臨床検査値の異常】

治験実施医療機関における基準値の範囲を外れる値を臨床検査値の異常と定義する。

【臨床的に考慮すべき検査値または検査値の変動】

臨床的に考慮すべき検査値または検査値の変動とは、本剤投与直前の検査値をベースラインと規定し、「有害事象共通用語規準 v5.0（CTCAE v5.0 日本語訳 JCOG/JSCO 版）」[70)]の規準に記載されている項目については、その規準における重症度が悪化した場合を、規準に記載されていない項目については、基準値範囲内→基準値範囲外、基準値範囲外→悪化を示す検査値または検査値の変動をいう。

なお、臨床的に考慮すべき検査値または検査値の変動があった項目について、治験責任医師または治験分担医師は臨床的に有意な変動であるか否かを判断し、症例報告書に記載する。有意でないと判断した場合には、その理由を症例報告書に記載する。

基準値は治験実施医療機関で用いられるものに基づくこととし、治験実施中に変更があった場合には、変更がされた時点以降、変更後の基準値を用いることとする。

11.1.2 重篤な有害事象（SAE）

以下に示す重篤な有害事象の定義は、「治験中に得られる安全性情報の取り扱いについて（薬審発第 227 号　平成 7 年 3 月 20 日）および「ICH-E2A “Clinical Safety Management: Definitions and Standards for Expedited Reporting”（Oct 27, 1994）」を基に定義されているものである。

重篤な有害事象とは、同意取得日から投与後 12 週までの間に被験者に生じた、あらゆる好ましくない医療上の出来事のうち、「表 11-2 重篤な有害事象」に示すものを言う。

表 11-2 重篤な有害事象

1)　死に至るもの	有害事象が死亡の原因である、もしくは死亡に至る要因である事象。死亡は有害事象の転帰であり、その死亡に至らしめた事象。
2)　生命を脅かすもの	有害事象の発現時に被験者を死の危険にさらしていた事象。ただし、その事象がもっと重症であったなら死に至っていたかもしれないという仮定的な意味ではない。
3)　治療のための入院または入院期間の延長が必要となるもの	少なくとも 24 時間の入院が必要となる事象、もしくは予定した入院期間を延長する必要がある事象。ただし、本治験の参加前より計画されていた入院や、社会的理由による入院、疾患の管理のため通常行われる入院（日程調整のための入院、経過入院を含む）は、本項目には該当しない。
4)　永続的または顕著な障害・機能不全に陥るもの	被験者の日常生活に支障をきたす顕著な障害・機能不全。
5)　先天性異常を来すもの	受胎以前もしくは妊娠中に治験薬の曝露を受けた被験者の子供や胎児に発生した事象。
6)　その他、医学的に重要な事象	その他、死亡や生命を脅かしたり入院に至らなくとも、被験者を危険にさらしたり、上記のような結果に至らぬよう、処置を必要とするような重大な事象。

治験責任医師または治験分担医師は、同意取得日から投与後 12 週までの間に新たに発現した重篤な有害事象に関して、「11.4.5 緊急報告」に従い、可能な限り速やかに実施医療機関の長に報告するとともに、治験薬提供者に報告する。

11.1.3 副作用（ADR）

「副作用」とは、投与量にかかわらず、投与された本剤に対するあらゆる有害で意図しない反応（臨床検査値の異常を含む）をいう、すなわち、本剤と有害事象との間の因果関係について、少なくとも合理的な可能性があり、因果関係が否定できない反応をさす。

11.2 有害事象の記録

有害事象が発現した場合は、「表 11-3 有害事象の記録」に従って評価し、症例報告書に記載する。

表 11-3 有害事象の記録

<table>
<tr><td>有害事象名</td><td colspan="2">自覚症状・他覚所見欄に記載された複数の症状・徴候等が1つの診断名（疾患）として説明できる場合には、付随する個別の症状・徴候は記載せず、原則として当該事象に該当する診断名を記載</td></tr>
<tr><td>発現日</td><td colspan="2">新たに症状が発現した日、あるいは投与開始前から認められている症状については悪化を確認した日</td></tr>
<tr><td>重症度</td><td colspan="2">「有害事象共通用語規準 CTCAE v5.0 日本語訳 JCOG/JSCO 版）」[22]の規準に記載されている項目については、その重症度に従い、また「有害事象共通用語規準 CTCAE v5.0 日本語訳 JCOG/JSCO 版）」[22]に記載のない項目については、「有害事象共通用語規準 CTCAE v5.0 日本語訳 JCOG/JSCO 版」[22]に記載のない項目の重症度判定基準（表 11-1）に従い、Grade1～5 の 5 段階に分類する</td></tr>
<tr><td>重篤度</td><td>1. 非重篤
2. 重篤</td><td>「11.1.2 重篤な有害事象（SAE）」参照</td></tr>
<tr><td>処置</td><td>1. なし
2. あり</td><td>「2. あり」の場合は、処置内容を記載</td></tr>
<tr><td>転帰</td><td>1. 回復
2. 軽快
3. 未回復
4. 回復したが後遺症あり
5. 死亡
6. 不明</td><td>転帰日を記載
「1.回復」の場合は回復時期（日）を、「2.軽快、3.未回復、4.回復したが後遺症あり」の場合はその時期または確認時期（日）を、「5.死亡」の場合は死亡日を記載</td></tr>
<tr><td>本剤との因果関係</td><td>1. 関連なし
2. 関連あり</td><td>本剤との因果関係が否定できる場合
本剤との因果関係が否定できない場合</td></tr>
</table>

	3.　不明	情報不足により因果関係を判断できない場合

11.3　有害事象の予測性

11.3.1　予測できない有害事象

「予測できない有害事象」とは、有害事象のうち、治験薬概要書に記載されていない有害事象をいう。なお、記載されていても発生数、発生頻度、発生条件等の発生傾向、性質や重症度等が記載内容と一致しない場合（急性腎不全に対する「間質性腎炎」、肝炎に対する「劇症肝炎」等）は「予測できない」とする。本試験は first in human 試験であることから、試験開始時において全ての事象は予測できない有害事象となる。

11.3.2　予測できる有害事象

「予測できる有害事象」とは、有害事象のうち、治験薬概要書に記載され、かつ、その発生数、発生頻度、発生条件等の発生傾向等が記載内容と一致している有害事象をいう。本試験は first in human 試験であることから、試験開始時においては全ての事象は予測できない有害事象となる。

11.4　有害事象の発現、本剤投与後に妊娠が判明した場合の措置

11.4.1　治験実施計画書からの逸脱または変更

治験実施責任医師又は治験分担医師は、被験者の安全性確保のために治験実施計画書からの逸脱または変更を行うことができる（「15.3 治験実施計画書からの逸脱または変更」）。

11.4.2　有害事象発現時の措置

治験責任医師または治験分担医師は、有害事象が発現した場合、被験者の安全性確保のため必要に応じ被験者に対し、医療処置、ならびに本剤の投与中止等の適切な処置を講ずる。処置が必要となった場合にはその旨を被験者に伝える。治験分担医師が重篤な有害事象発生を知った場合、当該有害事象を知ってから24時間以内に治験責任医師へ報告する。

11.4.3　妊娠判明時の措置

治験責任医師または治験分担医師は、投与日（Day1）から投与 6 ヶ月までに妊娠した女性被験者および男性被験者のパートナーに関する妊娠情報を収集する。

投与日（Day1）から投与 6 ヶ月までの間に、女性被験者または男性被験者のパートナーが妊娠した場合には、治験責任医師または治験分担医師は直ちに当該被験者における治験を中止・終了し、妊娠を知り得てから 24 時間以内に「胎児の薬物曝

露に関する調査（登録用）」に妊娠情報を記載し、治験実施医療機関の長に報告する。

妊娠自体は有害事象または重篤な有害事象とみなされないが、先天異常、自然流産または医学的理由による人工流産は重篤な有害事象とみなし、「11.4.5 緊急報告」に従い、治験実施医療機関の長に報告するとともに、治験薬提供者に報告する。

また、治験責任医師または治験分担医師は妊娠の転帰を確認するために追跡調査（通常、出産日から 8 週まで）を行い、母子の状態に関する情報を「胎児の薬物曝露に関する調査（追跡調査用）」に記載し、24 時間以内に治験実施医療機関の長に報告するとともに、治験薬提供者に報告する。最終的な妊娠の転帰を入手するまで妊娠を追跡するよう、あらゆる努力を払う。

さらに、投与後 6 ヶ月以降に妊娠した結果、重篤な有害事象が発現し、治験責任医師または治験分担医師により本剤との因果関係が否定できないと判断された場合には、重篤な有害事象とみなし、「11.4.5 緊急報告」に従い、24 時間以内に治験実施医療機関の長に報告するとともに、治験薬提供者に報告する。

11.4.4 **評価および記録**

治験責任医師または治験分担医師は、発現した有害事象または判明した妊娠について、原資料（診療録等）に所見・症状・発現日、重症度、処置の有無（有の場合はその内容）、転帰（回復した場合は回復時期、症状が安定した場合はその時期）等を記録する。なお、治験責任医師は、症例報告書に記載された有害事象名を MedDRA/J（Medical Dictionary for Regulatory Activities/J、ICH 国際医薬品用語集／日本版）を用いて、医学的判断から最も適切と思われる用語を選択し、読み替えを行う。

11.4.5 **緊急報告**

治験分担医師が重篤な有害事象発生を知った場合、当該有害事象を知ってから２４時間以内に治験責任医師へ報告する。治験責任医師は、治験実施医療機関の長、規制当局および治験薬提供者への緊急報告を行う。

被験者が遠方から受診しており、すぐに治験実施医療機関を受診できない場合は、事前に決めた、かかりつけ医を受診するよう、被験者に伝える。その際、治験責任医師はそのかかりつけ医に直ちに連絡をとり、適切な処置を依頼するとともに、安全性情報を評価するのに必要な情報収集を行う。

重篤な有害事象に関する報告書式および手順は「安全性情報の取り扱いに関する手順書」に従う。

1) 治験実施医療機関の長への報告

治験責任医師は、重篤な有害事象の発現または妊娠（先天異常、自然流産ま

たは医学的理由による人工流産のみ)を知った場合、「安全性情報の取り扱いに関する手順書」に従い治験実施医療機関の長に報告する。

2) 規制当局への報告

治験責任医師は、医薬品、医療機器等の品質、有効性及び安全性の確保に関する法律(以下、薬機法という)施行規則第 275 条の 3 に規定される報告対象となる有害事象等の発現を知った場合は、当該施行規則に定める期限内に当局へ報告する。

3) 治験薬提供者への報告

治験責任医師は、重篤な有害事象の発現または妊娠(先天異常、自然流産または医学的理由による人工流産のみ)を知った場合、「安全性情報の取り扱いに関する手順書」に従い治験薬提供者に報告する。

11.4.6 新しい情報の提供

治験責任医師は、治験薬提供者等より被験者の安全に悪影響を及ぼし、治験の実施に影響を与え、または治験継続に関する治験審査委員会の承認を変更する可能性のある情報を入手した場合には、治験実施医療機関の長、治験に関与するすべての治験分担医師に速やかに提供する。

11.4.7 治験全体の中止

治験責任医師は、被験者の安全性の確保等の倫理上あるいは医療上やむを得ない事情のため、必要であれば治験全体を中止する(「16.2 治験の早期中止・中断」参照)。

12 治験薬情報

12.1 原薬

12.1.1 一般名

3-astatobenzylguanidine(^{211}At)(*meta*-astatobenzylguanidine(^{211}At))

12.1.2 化学名および構造式

化学名:3-astatobenzylguanidine(^{211}At) (*meta*-astatobenzylguanidine(^{211}At))

分子式:$C_8H_{10}{}^{211}AtN_3$

分子量:359.07

構造式:

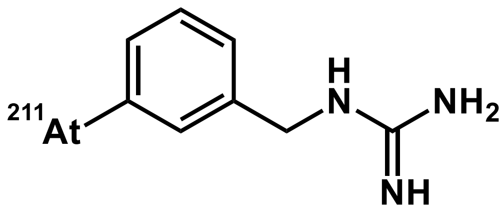

12.2 治験薬

治験薬は、公立大学法人福島県立医科大学ふくしま国際医療科学センター先端臨床研究センターによって自家製造され、品質管理が行なわれる。

治験薬の詳細および取扱いに関しては、治験薬概要書および「治験薬の管理に関する手順書」等を別途参照する。

12.2.1 剤形

本剤は、有効成分 ^{211}At-MABG を含む生理的 pH の静脈内投与注射剤であり、製造過程で添加されたエタノールを 3～5%含み、放射線分解を抑制するためアスコルビン酸ナトリウムを 0.1%含む。本剤の成分・分量を表 12-1 に示す。

表 12-1 ^{211}At-MABG 注射剤の成分・分量

有効成分	3-アスタトベンジルグアニジン(^{211}At)（メタアスタトベンジルグアニジン(^{211}At)）		
安定化剤	日局アスコルビン酸ナトリウム	安定化剤	0.1 w/v%
添加物	塩化ナトリウム（日局生理食塩水として）	等張化剤・溶剤	0.9 w/v%
添加物	エタノール	安定化剤	5 w/v%未満

12.2.2 物理化学的性質

外観・性状：無色～淡黄色の透明の溶液

pH：5.0～9.0

12.2.3 貯法

室温、暗所保存（鉛容器内保存）

12.2.4 有効期限

安定性試験の結果から、有効期限は、物理的半減期を考慮して検定時より 4 時間と設定した（4 時間後、^{211}At-MABG の放射能量は 68%に減少する）。また、室温で 20 時間保存した時の放射化学的純度は 90%以上であった。

12.2.5 治験薬の包装・表示

治験責任医師は、治験薬提供者から治験薬の提供を受ける場合は治験薬提供者に、以下を治験薬の容器に記載を求めるとともにその内容を確認する（本剤は院内製剤であるため被包はない）。

- 治験薬名
- 治験用である旨
- 治験責任医師の氏名および職名ならびに住所
- 科学名又は識別記号
- 製造番号
- 貯蔵方法、有効期間

13 治験薬の管理

13.1 治験薬の交付・保管・管理・回収

治験責任医師は、治験薬製造者から受領した治験薬を「治験薬の管理に関する手順書」等に従って、治験薬管理者へ交付する。

治験薬管理者は、「治験薬の管理に関する手順書」等に従って、治験薬を適切に保管・管理するとともに、治験薬管理表を作成する。また、治験終了後は、未使用の治験薬を返却する。

13.2 治験薬の無菌性試験

本剤は、用事製造される院内製剤であり、また、有効成分の半減期が極めて短い。そのため、無菌性試験は出荷判定後に行われることになる。治験薬投与後に無菌性試験の結果が得られることになるが、この試験結果に問題が認められた場合には、治験薬製造者は速やかに治験責任医師に文書にて結果を通知する。治験責任医師は、当該治験薬が投与された被験者を注意深く観察し、健康状態の維持に十分な対策を講じるとともに、症例報告書に得られた所見と、講じた処置について記録する。

14 統計解析

本項では統計解析計画の概要を記述し、詳細は別途規定する統計解析計画書に記す。統計解析計画書は、データベース固定時に作成されている版を最終版とし、これに従い最終解析を実施する。

被験者背景、安全性、及び PK データについて、コホートごとあるいは必要に応じて時点ごとに要約統計量を算出する。連続データにおける要約統計量には、平均値、中央値、標準偏差、及び範囲（Cmax、AUC、 PK パラメータにおける幾何平均及び幾何学的変異係数等）、カテゴリカルデータについては頻度と割合を算出する。必要に応じて、データの図示も行う。ベースラインから治療後までの変化又はベースラインに対する治療後の変化率の評価は、ベースライン及び治療後に測定を行った被験者で実施する。特に明記しない限り、治験薬初回投与前に測定した直近のデータをベースラインのデータとして使用する。

14.1　症例数

各コホート3例もしくは6例、3コホートを実施し、最大症例数として 18 例

【設定根拠】

「抗悪性腫瘍薬の臨床評価方法に関するガイドライン」に従い、抗悪性腫瘍薬の第Ⅰ相臨床試験における最大耐量（MTD）を推定するための”3+3”デザインにおける最大 3 用量のための例数として設定した。

14.2　解析対象集団

14.2.1 最大の解析対象集団（Full Analysis Set： FAS）

本試験に登録され、治験薬が一度でも投与された症例を「最大の解析対象集団（FAS）」とする。ただし、投与後に適格性がないことが判明した症例、重大な試験実施計画書違反（同意未取得、試験手続き上の重大な違反）の症例については除外する。

14.2.2 治験実施計画書適合集団（Per Protocol Set: PPS）

最大の解析集団（FAS）のうち、以下の除外症例を除く症例。

【除外症例】（治験実施計画書適合除外例）

- 重大な治験実施計画書違反例

  治験期間中における以下の治験実施計画書違反が認められた症例とする。

  1) 選択基準を満たしていない、または除外基準に抵触する症例
  2) 治験期間中に「10 中止・終了基準」に該当したが、中止・終了されなかった症例
  3) 規定の投与量、投与回数または投与方法に不遵守が認められた症例
  4) 併用禁止薬剤を投与または併用禁止療法を実施した症例

14.2.3 **安全性解析対象集団（Safetty Analysis Set：SAS）**

治験薬が投与された被験者のうち、安全性に関して解析すべきデータがない被験者及び GCP 違反例を除いた集団を安全性解析対象集団と定義する。

14.2.4 **DLT 解析対象集団**

治験薬が投与された被験者のうち、安全性解析対象集団から、DLT 評価期間を終了せずに治療期を中止し、かつ DLT が発現しなかった被験者を除いた集団を DLT 解析対象集団とする。

14.2.5 **放射能薬物動態解析対象集団**

治験薬が投与された被験者のうち、いずれかの血漿中放射線濃度測定結果が得られた被験者及び GCP 違反例を除いた症例を対象とする。

14.3 データの取り扱い

データ集計・解析時、原則としてデータの取扱いは以下のとおりとする。

- 検査値、測定値の欠測値に対し補完を行わず、当該のデータを欠測として取り扱う。ただし、感度分析として、欠測の影響を評価する必要が生じた場合には、最悪値の補完などの補助的解析を行う。
- 同一期間内に複数の観測値がある場合は、規定の日に最も近い日付の観測値を用いる。

14.4 解析方法

14.4.1 **有効性の解析**

14.4.1.1 **PFS の解析**

PFS について、FAS 及び PPS にて全体及び各コホートで Kaplan-Meier 法を用い生存率を算出し、生存期間中央値(MST:median survival time)および 95%信頼区間（CI）を算出する。

14.4.1.2 **ORR の解析**

FAS 及び PPS にて全体及び各コホートで最良総合効果で CR または PR を示した被験者の割合（%）および 95%信頼区間（CI）を算出する。

14.4.1.3 **尿中カテコラミン類奏効率**

FAS 及び PPS にて全体及び各コホートでスクリーニング時に尿中カテコラミン類のいずれかが基準値上限の 3 倍以上であった患者が対象に、基準値上限の 3 倍以上であったすべての尿中カテコラミン類について、最良総合効果が CR（効果判定の対象とされたすべての尿中カテコ

ラミン類の値が基準値内まで減少）又は PR（効果判定の対象とされたすべての尿中カテコラミン類の値がベースライン時と比較して 50%を超えて減少）の場合に奏効と判定する。奏効率（%）および 95%信頼区間（CI）を算出する。

14.4.1.4 I-123 MIBG シンチグラフィによる腫瘍集積減少効果

画像評価の手順書に従い、全体及び各コホートで総合判定効果に基づく、シンチグラフィ総合評価（sCR、sPR）の被験者の割合（%）および 95%信頼区間（CI）を算出する。

14.4.1.5 QOL 評価

全体及び各コホートで、EORTC QLQ−C30 についてはスケールおよび項目毎にスコア化して平均値を時系列で示す。EQ−5D−5L の尺度毎のスコアの平均値を時系列で表示する。

14.4.2 安全性の解析

14.4.2.1 有害事象

投与開始日から Day43 まで及び投与終了後 12 週までにおける有害事象の発現頻度を集計し、全体及び各コホートで発現時期、内容、重症度、因果関係別に要約を行う。有害事象発現率は、SAS において有害事象が発現した被験者の割合とする。なお、有効性の解析と同様に詳細は別途、統計解析計画書を作成し規定する。次コホートへの移行の判断のため必要となる情報は必要に応じて算出する。

14.4.2.2 臨床検査値の推移

治験期間を通しての臨床検査値について、安全性解析対象集団において全体及び各コホートで推移図等を作成する。詳細は別途規定する統計解析計画書に記載する。詳細は別途規定する統計解析計画書に記載する。次コホートへの移行の判断のため必要となる情報は必要に応じて算出する。

14.4.3 DLT の解析

データ解析は DLT の判定や次コホートへの移行を判断するため、各コホート中にも必要に応じて実施する。各コホートにおける DLT の事象名、発生割合を算出する。

14.4.4 放射能薬物動態の解析

コホートごとに血清中及び尿中濃度が得られている全ての被験者のデータの一覧を示す。記述統計量は、平均値、標準偏差、中央値及び範囲を表としてコホートごと

に、要約する。薬物動態パラメータを個々に一覧とし、記述統計量をコホートごとに要約する。被験者のデータが，薬物動態の適正な評価に対して不十分な場合（例えば，治験薬の不完全な投与、投与及び採取時間情報の欠測、薬物動態パラメータの算出に不十分な薬物濃度データ）、薬物動態解析から当該被験者を除外する。血清中及び尿中濃度が定量下限未満である、又は欠測値である場合は全て、薬物濃度データ一覧表や SAS データセット中等でこれを明示する。記述統計量では定量下限未満の濃度はゼロとして扱う。解析で除外する被験者及び試料の全てを治験総括報告書に明記する。治験薬の初回投与後の濃度－時間推移平均値及び中央値をプロットする。個別の血清中濃度－時間推移もプロットすることがある。非線形混合効果モデルを用いて血清中濃度－時間データに関する母集団薬物動態解析を行う場合がある。データはその他の選択された治験データと統合することがある。詳細は母集団薬物動態解析計画書に示し、別途作成する報告書に母集団薬物動態解析の結果を示す。曝露量反応解析を実施することもできる。実施する場合は，別途作成する解析計画及び報告書に詳細を記載する。

14.4.5 **中間解析**

本治験では、中間解析は行わない。

15 治験実施計画書の遵守および逸脱・変更並びに改訂

15.1 治験実施計画書の承認および改訂

治験責任医師は、治験開始前に治験実施計画書を治験実施医療機関の長へ提出し、治験の実施に関して治験審査委員会および治験実施医療機関の長の承認を得る。

治験実施計画書の改訂を行う場合、治験責任医師は、変更の妥当性および治験の評価への影響について、必要に応じて統計解析責任者等と協議した上で改訂を決定する。改訂した治験実施計画書を治験実施医療機関の長に提出し、治験審査委員会および治験実施医療機関の長の承認を得なければならない。重大な変更の場合は、被験者保護の観点から症例登録の一時中止を検討し、事前に効果安全性評価委員会の審議を受ける。

改訂の承認を得た後、治験責任医師は、改訂内容を治験分担医師、外部委託する治験管理組織、治験関係者に速やかに伝達する。

15.2 治験実施計画書の遵守

治験責任医師（自ら治験を実施する者）は、治験実施計画書および症例報告書の内容に合意し、また当該治験実施計画書を遵守することについて合意した旨を証す

るため、治験実施計画書またはそれに代わる文書に記名捺印あるいは署名し、日付を記入する。

15.3 治験実施計画書からの逸脱または変更

治験責任（分担）医師は、治験実施計画書からの逸脱行為を全て記録する。治験責任医師は、逸脱した行為のうち軽微なものについては「逸脱（緊急以外）報告書（院内書式 8-2）」を作成し、治験実施医療機関の長および治験審査委員会へ提出し、了承を得る。逸脱した行為のうち、被験者の緊急の危険を回避するためその他医療上やむを得ない理由により治験実施計画書に従わなかったものについてのみ、その理由を説明した記録を作成し直ちに治験実施医療機関の長に提出し、その写しを保管する。

治験責任医師または治験分担医師は、被験者の緊急の危険を回避するためのものである等医療上やむを得ない事情のために、治験審査委員会の事前承認なしに治験実施計画書からの逸脱または変更を行うことができる。その際には治験責任医師は逸脱または変更の内容および理由ならびに治験実施計画書の改訂が適切な場合にはその案を「緊急逸脱報告書（統一書式8）」に記載し、可能な限り早急に治験実施医療機関の長および治験審査委員会に提出し、承認を得る。

16 治験の終了または中止および中断

16.1 治験の終了

全症例の治験スケジュールの完了、全ての症例報告書の提出およびデータ固定、GCP 監査の終了後、治験総括報告書（QA 陳述書含む）の完成をもって、治験終了とする。

治験責任医師は、治験が終了したことを治験分担医師、外部委託する治験管理組織、効果安全性評価委員会および本治験の関係者に報告する。また、治験責任医師は、治験終了後速やかに、治験実施医療機関の長に対して、治験終了を文書で報告する。

16.2 治験の早期中止・中断

本治験開始後に新たに得られた情報により、治験薬の安全性または有効性に関して治験継続に重大な問題があることが明らかになった等の理由で、効果安全性評価委員会から早期中止および中断の提言があった場合、または治験実施施設が GCP 省令または治験実施計画書に違反することにより適正な治験に支障を及ぼしたと認める場合、治験責任医師は、治験の早期中止または中断の判断をおこなう。効果安全性評価委員会の提言に従わない場合は、治験責任医師はその理由を効果安全性評価委員会に報告する。

治験責任医師が治験の早期中止および中断の決定を行った場合は、その理由および以後の対応を直ちに治験分担医師、外部委託する治験管理組織および本治験の関係者に連絡し、中止後の処理にあたる。また、治験責任医師は、治験の早期中止および中断後速やかに、治験実施医療機関の長に対して、治験の早期中止および中断を文書で報告する。

治験責任（分担）医師は、治験が早期中止または中断された場合、理由にかかわらず、本治験に参加した被験者に速やかにその旨を通知し、適切な措置を講じ被験者の安全を確保するための検査等を実施する。

17 症例報告書

17.1 様式

本治験では、紙の症例報告書を使用する。

17.2 記入方法

1) 治験責任（分担）医師は、「症例報告書の記入・修正の手引き」に従い、症例報告書を作成する。
2) 治験責任医師はその内容を点検および確認した後に、署名ページに手書きで日付（署名日）を記載し署名する。
3) 署名後の症例報告書の内容の訂正を行う場合には、再度 2)の手順に従い署名する。

17.3 提出方法

治験責任医師は、作成した症例報告書を保管し、外部委託する治験管理組織へ症例報告書の写しを提出する。提出後の修正は、Data Clarification form(DCF)を用いて行う。記入および提出は「17.2 記入方法」「17.3 提出方法」に準じて行う。

18 モニタリング

18.1 モニタリングの方法

本治験が治験実施計画書および関連する法規等に従って安全かつ適切に実施されていること、およびデータの信頼性が十分に確保されていることを確認する目的で、モニタリング責任者または担当者による原資料と症例報告書の照合を含む直接閲覧のため、施設訪問モニタリングを実施する。

治験責任（分担）医師は、事前に指名したモニタリング責任者および担当者による施設訪問モニタリングを受け入れ、原資料等の全ての治験関連記録を直接閲覧に供しなければならない。

原資料と症例報告書に何らかの矛盾がある場合には、モニタリング責任者または担当者は、その矛盾について治験責任医師に報告し、必要があれば症例報告書の訂正ならびに再提出を依頼する。

モニタリングの実施時期および詳細については、モニタリング計画書に別途定める。

18.2 原資料およびその他の記録の特定

本治験における原資料とは、以下のものをいう。

- 被験者の同意および被験者への情報提供に関する記録
- 診療記録、看護記録、症例登録票、症例登録確認書、本治験で規定された評価・検査・観察記録等、症例報告書作成のもととなった記録
- 本治験で規定された治療に関する記録

ただし、以下の項目については、治験責任（分担）医師により症例報告書に直接入力される場合、症例報告書の記載内容が原データとなる場合がある。

- 選択基準・除外基準の判定
- 既往歴・合併症の重症度
- 併用薬・併用療法の使用理由
- 有害事象の重篤度、重症度、転帰、治験薬との因果関係
- 本治験の中止理由、転帰に関するコメント
- その他のコメント、所見

19 治験の品質管理および品質保証

治験責任医師は、自らが定める業務手順書に基づく品質管理および品質保証のシステムを履行することによって、治験の実施、データの作成、記録および報告が、以下を遵守して行われることを保証する。

- 治験実施計画書
- 薬機法第 14 条第 3 項および第 80 条の 2 に規定する基準
- 医薬品の臨床試験の実施の基準に関する省令（GCP 省令）

また、治験責任医師は、本治験が治験実施計画書および関連する法規等に従って安全かつ適切に実施されていること、および、データの信頼性が十分に確保されていることを確認するために、業務手順書に基づいてモニタリング責任者または担当者による施設訪問モニタリングを実施する。

さらに、監査担当者は、本治験が GCP 省令、治験実施計画書および手順書等を遵守しているか否かを、通常のモニタリングおよび治験の品質管理業務とは独立・分離して評価するために、品質保証活動の一環として第三者の立場から GCP 監査を実施する。

19.1　原資料の直接閲覧の保証

治験実施医療機関の長および治験責任医師は、モニタリング、監査ならびに規制当局および治験審査委員会による調査の際に、原資料等すべての治験関連記録を直接閲覧に供し、これに協力するものとする。

19.2　効果安全性評価委員会

治験責任医師は、治験が安全かつ適切に実施されているかを監視する目的、および、収集されたデータに基づき、DLT 及び RD の判定を行うために効果安全性評価委員会を設置する。また、登録症例について解析対象集団に関係する取扱い、および以下に示す事項に対する対応について効果安全性評価委員会に諮問する。

- 治験実施計画書の重大な変更
- 重篤な有害事象の発現
- モニタリング等で重大な問題が認められた事項
- その他、治験責任医師が効果安全性評価委員会による客観的審議が必要と判断した事項

20　記録等の保存

治験責任医師は、自ら治験を実施する者が保管すべき必須文書を「記録の保存に関する手順書」に従って、以下のいずれか遅い日まで保存する。

1) 当該治験薬に係る製造販売承認日から 5 年が経過した日（開発中止が決定された場合には開発中止が決定された日から 3 年が経過した日）。ただし、薬機法の規定により承認後の再審査を受けなければならない場合で、かつ再審査が終了するまでの期間が 5 年を超えるものについては、再審査が終了する日。

2) 治験の中止または終了後 3 年が経過した日。

治験実施医療機関の長は、治験実施医療機関において保管すべき必須文書を以下のいずれか遅い日まで保存する。

(1) 当該治験薬に係る製造販売承認日（開発の中止若しくは治験の成績が承認申請書に添付されない旨の通知を受けた場合には開発中止が決定された若しくは申請書に添付されない旨の通知を受けた日から 3 年が経過した日）。

(2) 治験の中止または終了後 3 年が経過した日。

21　金銭の支払いおよび保険

21.1　治験に関する費用と被験者負担について

本治験においては、治療期間(治験薬投与日から投与後 12 週・中止日まで)が保険外併用療養費の対象となる。

また本治験においては先端臨床研究センター運営事業補助金(公的資金)を財源として実施する。被験者に対して入院一泊につき1万円、外来受診 1 回につき1万円の負担軽減費を支払う。

21.2　健康被害に対する補償について

治験責任医師および治験実施医療機関は、本治験に関連して被験者に生じた健康被害(治験の実施の準備、管理または実施に係る業務の全部又は一部を委託した場合に生じたものを含む)に対する補償措置として、保険への加入、健康被害の治療に関する医療体制提供の整備、「被験者の健康被害補償に関する手順書」を作成し、これに備える。

22　公表に関する取り決め

本治験で得られた成果は、福島県立医科大学附属病院に帰属する。本治験で得られた成果を論文または学会にて発表する場合は、治験責任医師が取り決める。

23　治験の倫理的および科学的実施

23.1　遵守すべき諸規則

本治験に関与するすべての者は、「世界医師会ヘルシンキ宣言(2013 年修正)」、薬機法、薬機法施行規則、「医薬品の臨床試験の実施の基準に関する省令」(平成 9 年 3 月 27 日厚生省令第 28 号)、「医薬品の臨床試験の実施の基準に関する省令の一部を改正する省令」(令和 2 年 12 月 25 日厚生労働省令第 208 号)、「薬機法施行規則の一部を改正する省令」(平成 29 年 10 月 5 日厚生労働省令第 106 号)および関連する最新の法令・通知に従う。

23.2　説明文書・同意文書の作成と改訂

説明文書・同意文書は、GCP 省令等に従って治験責任医師が作成する。ただし、被験者を意図的に誘導するような記載をしてはならない。また、作成した説明文書・同意文書は治験開始前に治験審査委員会と実施医療機関の長の承認を得る。

治験開始後に治験責任医師が被験者の同意に影響を与える新たな知見を得、説明文書・同意文書の改訂が必要と判断した場合には、それを改訂し、予め治験審査委員会と医療機関の長の承認を得る。被験者の同意に影響を与える新たな知見と

は、例えば当該治験薬等に関連する新たな有害事象の情報、あるいは対象疾患に関わる新治療法等の開発に関する情報などを指す。

23.3 説明と同意（インフォームド・コンセント）

治験責任（分担）医師は、被験者が治験に参加する前に、被験者に対して説明文書を用いて十分説明し、治験への参加について自由意思による同意を被験者から文書として得る。文書による同意を得る際には、被験者が説明文書の内容を十分理解した上で、説明を行った治験責任（分担）医師ならびに被験者は同意文書に各自日付を記入し、記名捺印または署名する。同意文書は治験実施医療機関で保存し、説明文書および同意文書の写しは被験者に渡す。

治験責任（分担）医師は、治験に継続して参加するかどうかについて被験者の意思に影響を与えると思われる情報を入手した場合には、直ちに当該情報を被験者に提供し、これを記録するとともに、被験者が治験に継続して参加するかどうかを確認する。また、治験責任医師が説明文書の改訂を行った場合は、治験責任（分担）医師は、改訂された説明文書を用いて改めて説明し、治験への参加の継続について自由意思による同意を被験者から文書により得る。

23.4 個人情報とプライバシーの保護

治験に関わる関係者は、被験者の個人情報保護について、適用される法令、条例を遵守する。 また関係者は、本治験で知り得た個人情報及び臨床情報などのプライバシーに関する情報は、個人の人格尊重の理念の元、厳重に保護され、慎重に扱われるべきものと認識し、被験者の個人情報およびプライバシー保護に最大限の努力を払い、これに努める。

治験責任（分担）医師が症例登録票および症例報告書等を提出する際には、被験者識別コードまたは登録番号を用い、実施医療機関以外の者が被験者を特定できる情報は記載しない。

外部委託する治験管理組織は、被験者識別コードまたは登録番号を用いてデータ管理を行う。 また、治験責任（分担）医師等が治験で得られた情報を公表する際には、被験者が特定できないよう十分に配慮する。

本治験を行う上で知り得た個人情報及び臨床情報は「個人情報の保護に関する法律」（平成 15 年 5 月 30 日施行法律第 57 号）に準拠し、「福島県個人情報保護条例」（平成 28 年 4 月 1 日施行、以下福島県条例という）第 7 条に基づき、正当な理由なく漏らしてはいけない。また、福島県条例第 10 条に基づき、関係者がその職を退いた後も同様とする。

24　治験責任医師の責務

治験責任医師の主な業務内容を以下に記載する（平成 9 年 3 月 27 日厚生労働省令第 28 号「医薬品の臨床試験の実施の基準に関する省令」第 42〜55 条に従う。）

1) 治験審査委員会への文書の提出および治験実施医療機関の長の指示・決定に従って治験を実施すること
2) 治験分担医師・治験協力者を確保できること
   (1) 分担する業務のリストを作成して、治験実施医療機関の長に提出し、その了承を受けること
   (2) 治験分担医師等に治験に関する情報を与え、指導、監督すること
3) 倫理的・科学的観点および治験実施計画書に定められた選択基準および除外基準に基づき、適切な被験者を選定すること
4) 被験者から治験の参加について文書による同意を取得すること
   (1) 治験薬提供者の協力を得て、同意文書およびその他の説明文書を作成すること
   (2) 同意文書およびその他の説明文書により被験者に適切に説明すること、そしてそれらの文書を被験者に交付すること
   (3) 同意文書に説明した者の署名または記名押印を得ること。補助説明者がいる場合、補助説明者の署名または記名押印も得ること。
   (4) 同意文書の写しを被験者に交付すること
   (5) 被験者の意思に影響を与える新たな情報が得られた場合、直ちに被験者に当該情報を与えること
   (6) 新たな情報が得られた場合、必要に応じて説明・同意文書を改訂し、被験者に治験の継続参加について改めて文書による同意を得ること
5) 被験者へ本剤の投与方法を説明し、その投与状況を確認すること
6) 被験者に対して治験に関連する医療の全ての判断に責任を負うこと
   (1) 他の医師の治療を受けている場合、治験参加の旨をその医師に連絡すること
   (2) 有害事象に対して適切な医療が行われるよう、事前に措置を講じておくこと
   (3) 有害事象が生じ治療が必要な時は、その旨を被験者に通知すること
7) 被験者が途中で参加を取り止めようとする場合、または取り止めた場合には、被験者の権利を十分に尊重した上で、その理由を確認するための努力を払うこと
8) 治験実施計画書から被験者の緊急の危険を回避するため以外の逸脱をしないことおよび逸脱した場合はその記録を作成すること
9) 治験実施計画書から被験者の緊急の危険を回避するための逸脱をした場合は、その記録を作成し、治験実施医療機関の長・治験審査委員会に提出すること

10) 重篤な有害事象が発現した場合、速やかに治験実施医療機関の長へ報告すること
11) 治験が終了（または中止）した場合、治験実施医療機関の長に文書で報告すること
12) 正確な症例報告書を作成すること
    (1) 治験分担医師が作成した症例報告書を点検し、内容を確認すること
    (2) 治験分担医師が行った変更または修正についても点検し、問題がないことを確認すること
    (3) 被験者の識別に症例番号を用いていることを保証すること
13) 治験の実施に関わる治験に係る文書または記録を治験実施医療機関の長の指示に従い保存すること
14) モニター、監査担当者および治験審査委員会、規制当局による調査を受け入れ、それらの求めに応じ、原資料等の関連記録を直接閲覧に供すること

25 治験実施体制

本治験は福島県立医科大学単施設で行う医師主導治験である。

25.1 治験実施医療機関

福島県立医科大学附属病院
〒960-1295 福島県福島市光が丘 1 番地
TEL：024-547-1252 FAX：024-548-2735

25.2 治験責任医師

福島県立医科大学附属病院 核医学科
医師 志賀 哲

25.3 治験・開発計画支援責任者

福島県立医科大学 医療研究推進センター
小早川 雅男

25.4 治験薬製造者

福島県立医科大学 ふくしま国際医療科学センター 先端臨床研究センター
教授 髙橋 和弘
〒960-1295 福島県福島市光が丘 1 番地
TEL：024-547-1674 FAX：024-547-1666

25.5 統計解析責任者

公益財団法人がん研究会有明病院
臨床研究センター 企画品質管理部
副部長 石塚 直樹
〒135-8550 東京都江東区有明 3-8-31

25.6 薬物動体解析責任者

明治薬科大学 薬物動態学研究室
教授 花田 和彦
〒204-8588 東京都 清瀬市 野塩 2-522-1

25.7 効果安全性評価委員

長崎大学 原爆後障害医療研究所
原爆･ヒバクシャ医療部門
アイソトープ診断治療学研究分野
教授 工藤 崇

国立研究開発法人 量子科学技術研究開発機構 量子生命･医学部門
量子医科学研究所 分子イメージング診断治療研究部
部長 東 達也

近畿大学医学部 放射線医学教室
教授 細野 眞

25.8 その他の治験管理組織

以下の外部委託する治験管理組織については別紙1に示す。

・ データマネジメント責任者
・ モニタリング責任者
・ 監査責任者

26　引用文献

1) Solcia E, et al. eds. Tumours of Adrenal and Extra-adrenal Paraganglioma. In:WHO Histological Typing of Endocrine Tumoursm 2nd. IARC Press 2000:38.（I）

2) Lloyd RV ,et al. eds.：WHO Classfication of Tumours of Endocrine Organs In : World Health Organization Classfication of Tumours IARC Press 2017:180.1（I）

3) Hamidi O, et al.：Malignant Pheochromocytoma and Paraganglioma : 272 Patients Over 55 Years. J Clin Endocrinol Metab2017;102:3296-3305.(Ⅳ)

4) Lenders JW,et al.：Endocrine Society.Pheochromocytoma and paraganglioma:an endocrine society cliniycal practice guideline.J Clin Endocrinol Medab 2014;99:1915-1942.(Ⅵ)

5) Hartley L, et al.：Pheochromocytoma in Queensland 1970-1983.Aust NZ J Surg 1985;55:471-475.(Ⅳ)

6) Ayala-Ramirez M，et al.：Clinical risk factors for malignancy and overall survival in patients with pheochromocytomas and sympathetic paragangliomas : primary tumor size and primary tumor location as prognostic indication. J Clin Endocrionol Metab2011; 96（(3）:717-725.(Ⅳ)

7) van der Harst E,et al.：The value of plasma markers for the clinical behaviour of phaeochromocytomas.Eur J Endocrinol 2002;147:85-94.(Ⅳ)

8) Dhir M, et al.：Clinical Predictors of Malignancy in Patients with Pheochromocytoma and Paraganglioma.Ann Surg Oncol 2017；24:3624-3630.(Ⅳ)

9) Goffredo P, et al.：Malignant pheochromocytoma and paraganglioma : a population level analysis of long-term survival over two decades. J Surg Oncol2013;107 : 659-664.(Ⅳ)

10) Burnichon N, et al.：Somatic NF1 inactivation is a frequent event in sporadic pheochromocytoma. Hum Mol Genet 2012;21: 5397-5405.(Ⅳ)

11) Jafri M, et al.：Evaluation of SDHB，SDHD and VHL gene susceptibility testing in the assessment of indviduals with nonsyndromic phaeochromocytoma，paraganglioma and head and neck paragaglioma. Clin Endocrinol(Oxf）2013;78:898-906.(Ⅳ)

12) Timmeers HJ, et al.：Superiority of fluorodeoxyglucose positron emission tomography to other functional imaging techniques in the evaluation of metastatic SDHB-associated pheochromocytoma and paraganglioma.　J Clin Oncol 2007;25:2262-2269.(Ⅳ)

13) Nagura S, et al.：Immunohistochemical estimations of growh activity to predict biological behavior of pheochromocytomas. Mod Pathol 1999;12:1107-111.(Ⅳ)

14) Thompson LD：Pheochromocytoma of the Adrenal Gland Scaled Score（PASS）to separate benign from malignant neoplasms:a clinicopathologic and immunophenotypic study of 100 cases.Am J Surg Pathol 2004;26:551-566.(Ⅳ)

15) Ocal I, et al.：Lack of correlations among histopathological parameters, Ki-67 proliferation index and prognosis in pheochromocytoma patients. Asian Pac J Cancer Prev2014;15:1755.(Ⅳ)

16) Kimura N, et al.：Pathological grading for predicting metastasis in phaeochromocytoma and paraganglioma. Phaeochromocytoma Study Group in Japan.Endocr Relat Cancer 2014;21:405-414. (Ⅳ)

17) Kimura N, et al.：Histological grading of adrenal and extra-adrenal pheochromocytomas and relationship to prognosis: a clinicopathological analysis of 116 adrenal pheochromocytomas and 30 extra-adrenal sympathetic paragangliomas including 38 malignant tumors. EndocrPathol 2005;16:23‐32.(Ⅳ)

18) Frederieke M, et al.：High frequency of SDHB germaline mutations in patients with malignant Catecholamine-producing paragangliomas：implicatopms for genetic testing. J Clin Endocrinol Metab 2006;91:4505-4509.(Ⅳ)

19) Zhong X, et al.：Establishment and evaluation of a novel biomarker-based nomogram for malignant phaeochromocytomas and paragangliomas. Clin Endocrinol（Oxf）2017;87:127‐135. (Ⅳ)

20) 日本内分泌学会「悪性褐色細胞腫の実態調査と診療指針の作成」委員会 編：褐色細胞腫・パラガングリオーマ診療ガイドライン 2018.

21) Vaidyanathan G, Zalutsky MR. 1-(m-[211At]astatobenzyl)guanidine: synthesis via astato demetalation and preliminary in vitro and in vivo evaluation. *Bioconjugate chemistry* 1992; **3**: 499-503.

22) JCOG, JSCO：有害事象共通用語規準v5.0（CTCAE v5.0 日本語訳）

27　　付録

付録 1： ECOG Performance Status（PS）の評価

（日本語訳：NCI-CTC 日本語訳 JCOG 版 第 2 版，2001 年 より）
出典 Common Toxicity Criteria, Version2.0 Publish Date April 30, 1999
http://ctep.cancer.gov/protocolDevelopment/electronic_applications/docs/ctcv20_4-30-992.pdf
JCOG ホームページ http://www.jcog.jp/

Score	定義
0	全く問題なく活動できる。<br>発病前と同じ日常生活が制限なく行える。
1	肉体的に激しい活動は制限されるが、歩行可能で、軽作業や座っての作業は行うことができる。<br>例：軽い家事、事務作業
2	歩行可能で自分の身の回りのことは全て可能だが作業はできない。<br>日中の 50%以上はベッド外で過ごす。
3	限られた自分の身の回りのことしかできない。<br>日中の 50%以上をベッドか椅子で過ごす。
4	全く動けない。<br>自分の身の回りのことは全くできない。<br>完全にベッドか椅子で過ごす。

付録 2： RECIST ガイドライン 改定版 version 1.1

New response evaluation criteria in solid tumors: Revised RECIST guideline (version 1.1)
E.A. Eisenhauer, P. Therasse, J. Bogaerts, L.H. Schwartz, D. Sargent, R. Ford, J. Dancey, S. Arbuck, S.
Gwyther, M. Mooney, L. Rubinstein, L. Shankar, L. Dodd, R. Kaplan, D. Lacombe, J. Verweij
EUROPEAN JOURNAL OF CANCER 45 (2009) 228–247
（日本語訳 JCOG 版 ver.1.0 より）

<u>測定可能腫瘍病変（measurable tumor lesions）：</u>
少なくとも 1 方向で正確な測定が可能であり（測定断面における最大径（長径）を記録する）、
かつ以下のいずれかのサイズ以上のもの。
- CT で 10 mm（CT のスライス厚は 5 mm 以下）
- 臨床的評価としての測径器（caliper）による測定で 10 mm（測径器により正確に測定できない病変は測定不能として記録する）
-

<u>測定可能リンパ節病変（measurable malignant lymph nodes）：</u>
- 胸部 X 線写真で 20 mm。病的な腫大と判断され、かつ測定可能なリンパ節は、CT で評価した短軸の径（短径）が 15 mm 以上（CT のスライス厚は 5 mm 以下を推奨

<u>測定不能（non-measurable）病変：</u>
- 小病変（長径が 10 mm 未満の腫瘍病変または短径が 10mm 以上 15 mm 未満であるリンパ節病変）、および真の測定不能病変を含む、測定可能病変以外のすべての病変。真の測定不能病変とみなされる病変には次のものがある。軟膜髄膜病変、腹水、胸水または心嚢水、炎症性乳がん、皮膚や肺のリンパ管症、視触診では認識できるが再現性のある画像検査法では測定可能ではない腹部腫瘤や腹部臓器の腫大。

<u>病変の測定法</u>
すべての測定値はメートル法で記録する。臨床的評価（視触診）の場合は測径器を用いて測定する。すべてのベースライン評価は、治療開始前で、可能な限り治療開始に近い時期に行う。早くとも治療開始前 4 週以内に実施されなければならない。
標的病変や非標的病変として報告される各病変を記録するにあたっては、ベースラインおよび観察期間を通じて、同一の評価法かつ同一の技術を用いなければならない。追跡する病変が、画像評価はできないが臨床的評価はできるという場合を除いて、常に、臨床的評価ではなく画像診断に基づく評価を行わなければならない。

<u>「標的病変」および「非標的病変」のベースライン評価での記録</u>
ベースライン評価において 2 個以上の測定可能病変を認める場合、すべての浸潤臓器を代表する、合計が最大 5 個（各臓器につき最大 2 病変）までの病変を標的病変として選択し、これらについてベースライン評価での測定値を記録する。
標的病変は、病変のサイズ（最大径が測定可能な病変）に基づいて選択され、すべての浸潤臓器を代表するものであるべきである。さらにこれに加えて、再現性をも

った繰り返し測定が可能な病変でなければならない。時には、最大の病変が再現性のある測定に適さない場合もあるが、その場合は、再現性のある測定が可能な、次に大きな病変を選択すべきである。

リンパ節は、腫瘍の浸潤がない場合でも画像により描出され得る正常な解剖学的構造物であるため、特に言及しておく必要がある。3 項で記述したように、測定可能と定義され、標的病変に選択され得る病的なリンパ節腫大（pathological nodes）とは、CT での短径が 15 mm 以上であるものでなければならない。これらのリンパ節は短径のみをベースライン評価の径の和に加える。リンパ節の短径は、固形がんによる浸潤の有無を判定するために、画像診断医が通常用いている径である。リンパ節のサイズは通常、撮影面上の 2 方向で報告される（CT の場合、これはほぼ常に横断面である。MRI では、横断面、矢状断面、冠状断面のいずれもあり得る）。これらの測定値のうち小さい方の値が短径である。例えば、腹部リンパ節の測定値が 20 mm × 30 mm の場合、短径は 20 mm であり、測定可能なリンパ節病変とされる。この例では、リンパ節のサイズの測定値として 20 mm を記録すべきである。他の病的リンパ節腫大（短径が 10 mm 以上 15 mm 未満）はすべて、非標的病変とされる。ベースライン評価にて短径が 10 mm 未満のリンパ節は病的ではない（病変ではない）とみなされるため、記録または追跡すべきではない。ベースライン評価時の全標的病変の径の和（以下、径和。非リンパ節病変では長径、リンパ節病変では短径）を、ベースライン径和として算出し報告する。上述のとおり、リンパ節の径を径和に含める場合は短径のみを加える。ベースライン径和は、その後の客観的な腫瘍縮小効果における比較対照（基準）として用いられる。標的病変以外の、リンパ節病変を含む他のすべての病変（または病変部位）は非標的病変とし、これもベースライン評価時に記録する。これらの非標的病変は測定の必要はなく、「あり」、「なし」の別、また稀には「明らかな増悪」の有無について評価する。また、同一臓器内の複数の非標的病変を 1 病変として効果判定記録用紙に記録してもよい（「複数の腫大骨盤リンパ節」や「多発肝転移」など）。

<u>標的病変の評価</u>

・ 完全奏効（Complete Response：CR）：
  すべての標的病変の消失。
  標的病変として選択したすべてのリンパ節病変は、短径で 10mm 未満に縮小しなくてはならない。
・ 部分奏効（Partial Response：PR）：
  ベースライン径和に比して、標的病変の径和が 30% 以上減少。
・ 進行（Progressive Disease：PD）：
  経過中の最小の径和（ベースライン径和が経過中の最小値である場合、これを

最小の径和とする）に比して、標的病変の径和が 20%以上増加、かつ、径和が絶対値でも 5 mm 以上増加。

- 安定（Stable Disease：SD）：
  経過中の最小の径和に比して、PR に相当する縮小がなくPD に相当する増大がない。

<u>非標的病変の評価</u>

- 完全奏効（Complete Response：CR）：
  すべての非標的病変の消失かつ腫瘍マーカー値が基準値上限以下。すべてのリンパ節は病的腫大とみなされないサイズ（短径が 10mm 未満）とならなければならない。
- 非 CR/ 非 PD（Non-CR/Non-PD）：
  1 つ以上の非標的病変の残存かつ/または腫瘍マーカー値が基準値上限を超える。
- 進行（Progressive Disease：PD）：
  既存の非標的病変の明らかな増悪

<u>新病変（new lesions）</u>

ベースライン評価では撮影されなかった臓器や部位において、経過の検査で病変が同定された場合、それは新病変とみなされ、増悪と判定される。このような例として、ベースライン評価では体幹部の病変が認められた被験者において、試験中に脳の CT または MRI が実施され、転移が認められた場合がある。この被験者の脳転移は、たとえベースライン評価の脳画像がない場合でも PD の証拠とみなされる。

<u>総合効果</u>

各時点での効果：標的病変（非標的病変の有無にかかわらず）を有する場合

標的病変	非標的病変	新病変	総合効果
CR	CR	なし	CR
CR	Non-CR/non-PD	なし	PR
CR	評価なし	なし	PR
PR	Non-PD or 評価の欠損あり	なし	PR
SD	Non-PD or 評価の欠損あり	なし	SD
評価の欠損あり	Non-PD	なし	NE
PD	問わない	あり or なし	PD
問わない	PD	あり or なし	PD
問わない	問わない	あり	PD

CR：完全奏効、PR：部分奏効、SD：安定、PD：進行、NE：評価不能

各時点での効果：非標的病変のみを有する場合

非標的病変	新病変	総合効果
CR	なし	CR
Non-CR/non-PD	なし	Non-CR/non-PD*
評価なしがある	なし	NE
明らかな増悪	あり or なし	PD
問わない	あり	PD
CR：完全奏効、PD：進行、NE：評価不能<br>*いくつかの試験では有効性評価のエンドポイントとして SD の使用が増えており、測定可能病変がない場合にこのカテゴリーを適用することは推奨されないため、非標的疾患に関しては「安定」よりも「非 CR/非 PD」の方が望ましい		

<u>完全奏効や部分奏効の確定が必要ではない試験における最良総合効果の判定：</u>

こうした試験における最良総合効果は、全時点を通しての最良の効果と定義される（例えば、最初の評価で SD、2 回目の評価で PR、最終評価で PD なら、最良総合効果は PR である）。最良総合効果を SD とする場合には、プロトコールで定められたベースラインからの最短期間の規準をも満たさなければならない。最短期間の規準が満たされないことを除いて SD が最良の効果である場合、最良総合効果はその次の評価により異なる。例えば、最初の評価で SD、2 回目の評価で PD の時、SD の最短期間の規準を満たしていない場合には、最良総合効果は PD となる。同様の被験者が、最初の SD 評価の後に追跡不能となった場合には、最良総合効果は「評価不能」とされる。
