# Supplementary material for: Evaluation of pharmacokinetics, safety, and efficacy of [211At] meta-astatobenzylguanidine ([211At] MABG) in patients with pheochromocytoma or paraganglioma (PPGL): A study protocol
Source: PLoS One. 2024 May 28;19(5):e0303623. doi: 10.1371/journal.pone.0303623 (PMC11132457; doi:10.1371/journal.pone.0303623)
Supplement: S2 File — (PDF) [file pone.0303623.s003.pdf]

Evaluation of pharmacokinetics, safety, and efficacy of ^{211}At - MABG in pheochromocytoma/paraganglioma patients

(Phase I study)
MABG-01

Clinical trial protocol

Study Phase: Phase I

Fukushima Medical University

Version 1.3.0 20
September 9, 2022

略号一覧

略号	略してない表現	説明
ADR	adverse drug reaction	副作用
AE	adverse event	有害事象
ALP	alkaline phosphatase	アルカリフォスファターゼ
ALT（GPT）	alanine transaminase	アラニンアミノトランスフェラーゼ
AST（GOT）	aspartate aminotransferase	アスパラギン酸アミノトランスフェラーゼ
AUC	area under the curve	血中濃度曲線下面積
BNP	brain natriuretic peptide	脳性ナトリウム利尿ペプチド
BUN	blood urea nitrogen	血液尿素窒素
CFR	code of federal regulations	連邦規則集
Cl	chloride	クロール
CR	complete response	完全奏効
CRP	c-reactive protein	C 反応性タンパク
CT	computed tomography	コンピューター断層撮影
CTCAE	common terminology criteria for adverse events	有害事象共通用語基準
CVD	cyclophosphamide, vincristine, dacarbazine	シクロホスファミド、ビンクリスチン、ダカ ルバジン併用療法
DLT	dose limiting toxicity	用量制限毒性
DNA	deoxyribonucleic acid	デオキシリボ核酸
ECOG	Eastern Cooperative Oncology Group	米国東海岸がん臨床試験グループ
eGFR	estimated glemerular filtration rate	推定糸球体濾過量
FT3	free triiodothyronine3	遊離トリヨードサイロニン 3
FT4	free triiodothyronine4	遊離トリヨードサイロニン 4
EORTC	The European Organization for Research and Treatment of Cancer	全ヨーロッパにおけるがん・白血病研究組織
EQ-5D	EuroQol 5 Dimension	―
FAS	full analysis set	最大の解析対象集団

FDA	Food and Drug Administration	米国食品医薬品局
GCP	good clinical practice	医薬品の臨床試験の実施に関する基準
G-CSF	granulocyte colony stimulating factor	顆粒球コロニー刺激因子
γ-GTP	□-glutamyl transpeptidase	ガンマ-グルタミル･トランスペプチターゼ
HBc	Hepatitis B core	B 型肝炎核
HBs	Hepatitis B surface	B 型肝炎表面
HCV	Hepatitis C Virus	C 型肝炎ウイルス
hCG	human chorionic gonadotropin	血液中ヒト絨毛性ゴナドトロピン
HIV	Human Immunodeficiency Virus	ヒト免疫不全ウイルス
ICD	International Classification of Diseases for Oncology	国際疾病分類腫瘍学
JCOG	Japan Clinical Oncology Group	日本臨床腫瘍研究グループ
K	potassium	カリウム
LDH	lactate dehydrogenase	乳酸脱水素酵素
MedDRA/J	Medical Dictionary for Regulatory Activities/J	ICH 国際医薬用語集日本語版
MEN2	Multiple Endocrine Neoplasia type 2	多発性内分泌腫瘍 2 型
MRI	magnetic resonance imaging	核磁気共鳴画像
MST	median survival time	生存期間中央値
MTD	Maximum tolerated dose	最大耐量
Na	sodium	ナトリウム
NET	norepinephrine transporter	ノルエピネフリントランスポーター
NF1	neurofibromatosis type1	神経線維腫症 1 型
NYHA	New York Heart Association	心不全の重症度分類

ORR	overall response rate	奏効率
OS	overall survival	全生存期間
PCC	pheochromocytoma	褐色細胞腫
PD	progressive disease	進行
PDF	portable document format	ポータブル･ドキュメント･フォーマット
PET	positron emission tomography	陽電子放射断層撮影
PFS	progression free survival	無増悪生存期間
PGL	paraganglioma	パラガングリオーマ
PPGL	pheochromocytoma paraganglioma	褐色細胞腫/パラガングリオーマ
PPS	per protocol set	治験実施計画書適合集団
PR	partial response	部分寛解
RPK	radiopharmacokinetics	放射能薬物動態
PRO	patient reported outcome	患者報告アウトカム
PS	Performance Status	パフォーマンス･ステータス
QA	quality assurance	品質保証
QOL	quality of life	生活の質
RD	recommended dose	推奨投与量
RECIST	response evaluation criteria in solid tumor	固形がんの治療効果判定のためのガイドライン
SAE	serious adverse event	重篤な有害事象
SAS	safety analysis set	安全性解析対象集団
SD	stable disease	（病勢）安定
SpO2	saturation of percutaneous oxygen	血中酸素飽和濃度
STD10	severely toxic dose in 10% of animals	マウスにおける 10%に重篤な毒性が発現する投与量
TSH	thyroid stimulating hormone	甲状腺刺激ホルモン
t1/2	Biological half-life	血中濃度半減期
VHL	von Hippel Lindau	von Hippel Lindau 病
WHO	World Health Organization	世界保健機関

Summary of study design

Trial name	Evaluation of pharmacokinetics, safety, and efficacy of ^{211}At-MABG in pheochromocytoma/paraganglioma patients
Purpose of the study	To evaluate the tolerability of in-house 3-astatobenzylguanidine (MABG) conjugated with the alpha-emitting nuclide astatine-211 (^{211}At) in patients with pheochromocytoma/paraganglioma and to determine maximum tolerated dose (MTD) and recommended dose (RD). We will also exploratorily evaluate radiopharmacokinetics, tolerability, safety, and efficacy.
Rationale for conducting clinical trials	Malignant pheochromocytoma/paraganglioma is a tumor arising in the adrenal medulla. Evidence for diagnosis and treatment is not sufficient because it is a rare disease, and new treatment methods are desired. It was suggested that ^{211}At-MABG, a drug that binds to the nuclide ^{211}At that emits α rays, has a high ability to kill cancer cells and a high therapeutic effect on malignant cells of pheochromocytoma/paraganglioma in mice. The antitumor effect of this drug is also expected in human pheochromocytoma/paraganglioma.
Phase of trial	Phase I
clinical trial design	Dose escalation study
subject	Patients with pheochromocytoma/paraganglioma
selection criteria	All of the following conditions shall be met.<br>1) Patients from whom written consent can be obtained<br>2) Patients with histologically or clinically diagnosed pheochromocytoma, paraganglioma, malignant pheochromocytoma, or malignant paraganglioma<br>* The diagnostic criteria will be in accordance with the "2018 Clinical Practice Guidelines for Pheochromocytoma and Paraganglioma."<br>3) Patients diagnosed with pheochromocytoma are defined below (refractory pheochromocytoma is

defined as those who satisfy any of the following ① to ③ and are unable to undergo surgical resection or radical external irradiation)

① Pheochromocytoma/paraganglioma with an extensive local extension of the primary tumor at first presentation

② Malignant pheochromocytoma/malignant paraganglioma with distant metastasis at first presentation

③ Pheochromocytoma/paraganglioma with local recurrence or distant metastasis despite surgical resection

4) Aged 20 years or older at the time of informed consent

5) Patients with an ECOG Performance status (PS) of 0-2

6) ^{123}I-MIBG aggregation positive as determined by scintigraphy at the screening in one or more target lesions confirmed by CT imaging

7) Patients who meet all of the following criteria with test values at screening

① bone marrow function
   - (1) ≥ 3,000/μL without administration of granulocyte colony-stimulating factor (G-CSF)
   - (2) Non-transfusion hemoglobin ≥ 9.0 g/dL
   - (3) Non-transfused platelet count ≥ 10 x 10^4/mm^3

② Renal function
   - (1) Estimated glomerular filtration rate (eGFR) ≥ 30 mL/min/1.73m^2

③ Liver function
   - (1) AST ≤ 90 U/L
   - (2) ALT ≤ 126 U/L (male), ALT ≤ 69 U/L (female)
   - (3) LDH < 666 U/L

④ Heart function
   - (1) NYHA Functional class: I or lower

	⑤ Diabetes/endocrine disease<br>(1) HbA1c<8.0% (NGSP value)<br>⑥ breathing condition<br>(1) Atmospheric blood oxygen saturation (SpO2) ≥ 96%<br>8) Patients expected to survive for 3 months or longer<br>9) Patients who are expected to be independent in eating, excretion, and sleeping during the nuclear medicine treatment hospital isolation period<br>10) Patients for whom standard therapy (CVD therapy or ^{131}I-MIBG therapy) failed or that have no other appropriate therapy
Exclusion criteria	Exclude patients who meet any of the following criteria:<br>1) Patients with multiple active cancers<br>Active multiple cancers here are defined as synchronous multiple cancers and metachronous multiple cancers with a disease-free interval of 5 years or less. However, lesions equivalent to carcinoma in situ or carcinoma in situ that are judged to be cured by local treatment and have a disease-free period of more than 1 year after cure are not included in active multiple cancers. In addition, the following cancers associated with familial pheochromocytoma are not included in active double/multiple cancers.<br>① Medullary thyroid carcinoma in multiple endocrine neoplasia type 2 (MEN2)<br>② Retinal hemangioblastoma in von Hippel Lindau disease (VHL)<br>③ Neurofibromas in neurofibromatosis type 1 (NF1)<br>2) Patients who cannot stop administration of a drug that suppresses MABG accumulation for a predetermined period<br>3) Patients unable to stop taking α-methylparatyrosine for a predetermined period<br>4) Patients who have undergone surgery, CVD therapy, catheter hepatic artery embolization for liver

	metastasis, or radiotherapy within 8 weeks before enrollment<br>5) Patients who have received MIBG treatment within 12 weeks before enrollment<br>6) Patients who have developed Grade 2 or higher non-hematological toxicity during or after prior treatment, for which a causal relationship to treatment cannot be ruled out, and who require treatment during the study period<br>7) Patients who have any of the following infections and require medical treatment during the study period<br>① Hepatitis B virus infection<br>② Hepatitis C virus infection<br>③ HIV infection<br>④ Other infectious diseases requiring systemic treatment<br>8) Patients with a disease that requires continuous systemic administration of adrenocortical hormone (prednisone or prednisolone equivalent dose of 10 mg/d or more) or other immunosuppressants and treatment during the study period<br>9) Patients with a history of uncontrolled catecholamine seizures<br>10) Patients with a history of fatal arrhythmia or cardiac arrest<br>11) Patients with uncontrolled symptomatic arrhythmia, thyroid dysfunction, respiratory disease, and pleural effusions and ascites<br>12) Patients with coronary artery disease, arrhythmia requiring treatment with amiodarone, severe valvular disease, aortic disease, and bleeding tendency<br>13) Patients that are pregnant (even if it is determined that there is a possibility of pregnancy by a doctor's interview, the patient will be excluded from this study), within 28 d after giving birth, or breast-feeding (including women who have temporarily stopped breast-feeding)

	14) Female patients of childbearing potential or male patients with partners of childbearing potential who are unable to agree to contraception for 6 months after drug treatment (any 2 of the following: contraception methods include latex condoms (used by men), oral contraceptives, intrauterine device (IUD) (progesterone-free T type) used in combination; or tubal ligation and vasectomy)<br>15) Patients participating in other clinical trials within 3 months before the consent date<br>16) Other patients who are judged by the investigator or subinvestigator to be unsuitable for this study
Investigational new drug	3-astatobenzylguanidine (^{211}At) (^{211} At-MABG) (in-house)
study design	A single-arm open-label trial will evaluate the tolerability of a single intravenous bolus dose of 211At-MABG in patients with pheochromocytoma/paraganglioma and determine the RD of this agent. The doses of this drug are low (0.65 MBq/kg), medium (1.3 MBq/kg), and high (2.6 MBq/kg), starting with the low dose and investigating the dose according to a 3+3 design.
trial period	From the date of informed consent to 12 weeks after administration
Prohibited concomitant drugs/therapies	1) Surgical therapy<br>2) Chemical treatment<br>3) Granulocyte colony-stimulating factor (G-CSF)<br>* If the patient has a history of use, it must be at least 8 d since the last use.<br>4) Hormone therapy<br>5) Antibody therapy<br>6) Radiotherapy (including nuclear medicine treatment)<br>7) Other antitumor therapies (including so-called immunotherapy)<br>8) Continuous systemic administration of corticosteroids (>10 mg/d equivalent to prednisone)

	9) Systemic administration of immunosuppressants or immunostimulants<br>10) Topical use of corticosteroids at study drug administration sites<br>11) Ingestion of health food (Agaric) for the antitumor effect<br>12) Antiarrhythmic drugs (ventricular arrhythmias)<br>13) Sympatholytics (αβ blockers, α blockers, adrenergic neuroleptics)<br>14) Calcium antagonists: amlodipine, diltiazem<br>15) Sympathomimetics (inotropic, vasoconstrictive, β_2 -agonist)<br>16) Adrenergic receptor agonist<br>17) Antidepressants<br>18) Antipsychotics (neuroleptics)<br>19) Central nervous system stimulants<br>20) Blood transfusion before study drug administration<br>* If you have a history of blood transfusion, at least 29 d have passed since the last blood transfusion
Endpoints	[Primary endpoint]<br>Safety (dose-limiting toxicity: DLT, maximum tolerated dose: MTD, recommended dose: RD)<br>[Secondary endpoints]<br>1) Radiopharmacokinetics (RPK)<br>2) Urinary radioactivity excretion rate<br>3) Response rate of urinary catecholamines<br>4) Response rate (ORR)<br>5) Progression-free survival (PFS)<br>6) ^{123}I-MIBG scintigraphy on reducing tumor accumulation<br>7) Quality of life

Investigation/Observation/Test and Schedule

Investigations/processes	Before administration				Day of administration			After administration									
	COI	Screening	Registry	within 7 days	Before administration	Administration	After administration	next day	2 days after	3 days after	7 days after	14 days after	4 weeks after	6 weeks after	8 weeks after	12 weeks after	Suspension*14
		Day-42 ~-14	~Day-14	Day-7 ~-1	Day1			Day2	Day3	Day4	Day8	Day15	Day29 ±7	Day43 ±7	Day57 ±7	Day85 ±7	±14
		hospital ac			Hospital admission									hosp		Hos	入院
Informed consent	●																
Registry			●*12														
^{211}At-MABG administration						●											
Potassium Iodide(KI) administration				●*13	●*13			●*13									
5-HT_3 recepter antagonist administration					●												
Subject's background		●															
Concomitant drugs/Comobities			●→	→	→	→	→	→	→	→	→	→	→	→	→	→	●
**General condition**																	
ECOG Performance Status		●		●				●→	→	→	→	●	●	●	●	●	●
Height		●															
Weight		●		●								●	●	●	●	●	●
physical examination findings		●		●	●		1,2,3,4,6 hours after administration	●→	→	→	→	●	●	●	●	●	●
Bitals*1		●		●	●		1,2,3,4,6 hours after administration	●→	→	→	→	●	●	●	●	●	●
Blood oxygen saturation level (SpO_2)		●		●	●		1,2,3,4,6 hours after administration	●→	→	→	→	●	●	●	●	●	●
**Blood serum biochemistry test**																	
Hematology test*2		●		●	●		1,3,6 hours after administration	●		●	●	●	●	●	●	●	●
Blood biochemistry*3		●		●	●		1,3,6 hours after administration	●		●	●	●		●		●	●
Urine*4		●		●										●		●	●
Infection test*5		●															
Electocardiodiagram (12-lead ECG)		●			●		5 minutes and 1,3,6 hours after administration	●		●	●	●		●		●	●
Cardiac ultrasonography		●												●		●	●
Urine catecolamine*6		●												●		●	●
^{123}I-MIBG scintigraphy*7		●												●		●	●
^{123}I-MIBG accumulation*8		●															
QOL*9		●												●		●	●
**Radiopharmacokinetics**																	
Blood radioactivity concentration					●		5, 15 minutes and 1,3,6 hours after administration	24 hours	48 hours	72 hours							
Urine radioactivity concentration					●		during 0-3, 3-6, 6-12 hours after administration	12-24 hour	24-48 hour	48-72 hour							
Computed tomography (CT)*10		●												●		●	●
Pregnancy test (hCG) *11		●															
Aedverse events			●→	→	→	→	→	→	→	→	→	→	→	→	→	→	●

Target number of cases	Up to 18 cases
Scheduled clinical trial period	April 2022 to January 2024

Table of Contents

List of abbreviations .... - 1 -
Summary of study design .... - 4 -
1 History and background of development .... - 14 -
1.1 History of development .... - 14 -
1.2 Non-clinical study results .... - 21 -
1.3 Clinical trial results .... - 23 -
1.4 Potential risk-benefit assessment .... - 23 -
2 Purpose of the study .... - 23 -
2.1 Purpose of the study .... - 23 -
2.2 Type of trial .... - 24 -
3 Trial planning .... - 24 -
3.1 Study design .... - 24 -
3.2 Trial period .... - 24 -
3.3 Investigation of dosage and administration and definition of dose limiting toxicity .... - 24 -
3.4 Definition of Dose Limiting Toxicity (DLT) .... - 25 -
3.6 Endpoints .... - 27 -
3.7 Scheduled clinical trial period .... - 28 -
3.8 Number of cases .... - 28 -
4 Pretreatment, concomitant drug/concomitant therapy, posttreatment .... - 28 -
4.1 Pretreatment .... - 28 -
4.2 Concomitant drug/therapy .... - 28 -
5 Preparation and revision of consent/explanatory documents for subjects .... - 30 -
6 Target subjects .... - 30 -
6.1 Target subjects .... - 30 -
6.2 Selection criteria .... - 30 -
6.3 Exclusion criteria .... - 32 -
7 Obtaining consent .... - 33 -
7.1 Before registration .... - 33 -
7.2 When obtaining information that affects the subject's will .... - 34 -
7.3 When consent documents and other explanatory documents are revised. - 34 -
7.4 Subjects visiting other departments and hospitals .... - 34 -
8 Enrollment of subjects .... - 35 -
8.1 Procedure for case registration .... - 35 -

8.2 Additional registration - 35 -
9 Investigation/observation/test, evaluation items and timing - 35 -
9.1 Investigation/observation test and timing of implementation - 35 -
9.2 Obtaining consent - 38 -
9.3 Assigning subject identification code - 38 -
9.4 Hospitalization and admission to nuclear medicine unit - 38 -
9.5 Surveys, observations, and tests - 38 -
9.6 Evaluations - 46 -
10 Discontinuation/withdrawal criteria - 48 -
10.1 Discontinuation/withdrawal procedures - 48 -
10.2 Completion and discontinuation by subject - 49 -
11 Ensuring subject safety - 49 -
11.1 Definition of adverse events - 49 -
11.2 Recording of adverse events - 52 -
11.3 Predictability of adverse events - 53 -
11.4 Measures to be taken when adverse events occur or when pregnancy is confirmed after administration of this drug - 54 -
12 Investigational drug information - 56 -
12.1 Drug substance - 56 -
12.2 Investigational drug - 57 -
13 Investigational drug management - 58 -
13.1 Delivery, storage, management and collection of investigational drugs - 58 -
13.2 Investigational drug sterility testing - 58 -
14 Statistical analysis - 59 -
14.1 Number of cases - 59 -
14.2 Analysis target population - 59 -
14.3 Data handling - 60 -
14.4 Analysis methods - 61 -
15 Protocol compliance and deviations/changes and revisions - 63 -
15.1 Protocol approval and revision - 63 -
15.2 Protocol compliance - 63 -
15.3 Protocol deviations or changes - 63 -
16 Study completion, discontinuation and termination - 64 -
16.1 End of trial - 64 -
16.2 Early discontinuation/termination of clinical trials - 64 -
17 Case report form - 65 -

17.1 Type ... - 65 -
17.2 How to fill out ... - 65 -
17.3 Submission method ... - 65 -
18 Monitoring ... - 65 -
18.1 Monitoring methods ... - 65 -
18.2 Identification of source documents and other records ... - 66 -
19 Clinical trial quality control and quality assurance ... - 66 -
19.1 Guaranteed direct access to source documents ... - 67 -
19.2 Efficacy and safety evaluation committee ... - 67 -
20 Storage of records, etc. ... - 67 -
21 Monetary payments and insurance ... - 68 -
21.1 Costs and subject burdens related to clinical trials ... - 68 -
21.2 Compensation for health damage ... - 68 -
22 Disclosure arrangements ... - 68 -
23 Ethical and scientific conduct of clinical trials ... - 68 -
23.1 Rules to follow ... - 68 -
23.2 Preparation and revision of explanatory documents and consent documents . - 69 -
23.3 Explanation and consent (informed consent) ... - 69 -
23.4 Personal information and privacy protection ... - 70 -
24 Investigator responsibilities ... - 70 -
25 Clinical trial implementation system ... - 72 -
25.1 Clinical trial medical institution ... - 72 -
25.2 Investigator ... - 72 -
25.3 Clinical trial/development plan support manager ... - 72 -
25.4 Investigational drug manufacturer ... - 72 -
25.5 Chief of statistical analysis ... - 73 -
25.6 Pharmacokinetic analysis manager ... - 73 -
25.7 Efficacy and safety evaluation committee ... - 73 -
25.8 Other clinical trial management organizations ... - 73 -
26 Citations ... - 74 -
27 Appendix ... - 77 -

1 History and background of development

1.1 History of development

1.1.1 About pheochromocytoma/paraganglioma

Pheochromocytoma/paraganglioma is a tumor that arises from catecholamine-producing chromaffin cells of the adrenal medulla or paraganglioma. The former is called pheochromocytoma (PCC), the latter is paraganglioma (PGL), and both are collectively called pheochromocytoma/paraganglioma (PPGL). It has long been regarded as a neuroendocrine tumor[1)]. Because of the lack of conclusive evidence of potentially metastatic and 'benign' status in the WHO tumor classification of endocrine tumors published in 2017, all PPGLs have been assigned a disease code of malignancy (ICD-3), and it has been decided not to use the term “benign.” In other words, if no obvious metastatic or locally invasive lesions are observed at the time of diagnosis, it is recommended that the patient be diagnosed as “PPGL” and evaluated for malignancy in parallel[3)]. However, when there are clearly metastatic or locally invasive lesions, the term “malignant” is used to encompass both of these pathologies and to avoid confusion in terminology in clinical practice.

PGLs are derived from the sympathetic nervous system and the parasympathetic nervous system. The former mainly develops in the abdomen and pelvis, is noradrenaline-producing, and has a high degree of malignancy, whereas the latter occurs primarily in the head and neck region, has low hormone production capacity, and is less malignant.

1.1.2 Symptoms

Pheochromocytoma/paraganglioma presents various clinical manifestations, including hypertension, palpitations, tachycardia, chest pain, headache, pallor, sweating, and anxiety. Hyperglycemia, lactic acidosis, and weight loss are often observed on the metabolic side. With respect to hypertension, there are paroxysmal, persistent, and mixed cancer types. Hypertensive crisis sometimes occurs because of various triggers (diet, urination, anesthesia, lumpectomy, and drugs (β blockers, high-dose dexamethasone, glucagon, contrast media, tyramine, metoclopramide, tricyclic antidepressants)). However, there are many cases of asymptomatic, normotensive adrenal gland incidental tumors. In fact, 5-10% of adrenal incidental tumors are PCCs, and in recent years, about 25% of pre-PCC cases have been discovered as adrenal incidental tumors. Dopaminergic PGLs may present with normotension to hypotension. There are also cases showing orthostatic hypotension of unknown causes. Adrenergic tumors may present with hypotension and symptoms of shock. Mechanisms include

decreased intravascular volume, sudden decrease in catecholamine secretion because of tumor necrosis, desensitization of adrenergic receptors, and hypocalcemia. Occasionally, shock may occur because of myocardial infarction, arrhythmia, aortic dissection, intratumoral necrosis, or tumor rupture. Other causes include Takotsubo cardiomyopathy, hypertensive encephalopathy, sudden death because of cerebrovascular disease, and heart failure.

1.1.3 **Screening**

Personal or family history of PPGL, a hereditary disease with PCC (multiple endocrine neoplasia type 2 (MEN2), von Hippel Lindau disease (VHL), neurofibroma type I (NF1)), palpitations, sweating, headache, chest pain, episodic hypertension, treatment-resistant hypertension, diabetes-complicated hypertension, adrenal gland incidental tumors, etc[4)]. The cost-effectiveness of screening in patients with normal hypertension has not been established.

1.1.4 **Epidemiology**

The exact prevalence of pheochromocytoma is unknown. A report from Australia [5)] puts it at 1.55 cases per million adults per year. Assuming that 20% of adults have hypertension translates to approximately five cases of pheochromocytoma per year in every 1 million hypertensive patients. However, because many patients present with nonspecific symptoms such as abdominal pain, vomiting, dyspnea, and hypotension, many patients are not diagnosed with pheochromocytoma during their lifetime. In Japan, a nationwide epidemiological survey was conducted by the Ministry of Health and Welfare's Adrenal Hormone Production Abnormality Research Group (Takeda Group, Nawada Group) (Table 1-1).

Table 1pheochromocytoma/paraganglioma

	Research Group for Adrenal Hormone Production Disorders (Takeda group) [*1)]	Research Group for Adrenal Hormone Production Disorders (Nawada group) [*2)]	Pheochromocytoma research group (Naruse group) [*3)]
Investigation period	1973-1982 (10 years)	1997 (1 year)	2008 (1 year)
Target facility	Hospitals with 300 beds or more	Stratified random sampling from all	Stratified random sampling from all

		university hospitals and general hospitals	university hospitals and general hospitals
Target department	Internal Medicine, Pediatrics, Surgery, Urology	Internal Medicine, Endocrinology, Pediatrics, Neurosurgery, Urology	Internal Medicine, Cardiology, Surgery, Pediatrics, Urology, Radiology, Pediatric Surgery
Number of target clinical departments		4,060	5,912 (Hospital signage 6,303)
Number of primary survey cases	862	522	1,649
Estimated number of patients [95% CI]		1,030 [860–1,200]	2,920 [2,580–3,260]

*1) Ministry of Health and Welfare, Ministry of Health, Labor and Welfare Research Group for Specified Endocrine Diseases "Regarding the national total of pheochromocytoma and the national total of tertiary steroid hormone production disorder."
*2) Ministry of Health, Labor and Welfare Research Group for Specified Endocrine Diseases "National Epidemiological Survey of Adrenal Hormone Production Disorders."
*3) Health, Labor and Welfare Science Research Grants Research Project to Overcome Intractable Diseases "Fact-finding survey of pheochromocytoma and preparation of clinical guidelines."

Based on the nationwide epidemiological survey conducted in 2009 (Naruse group), the estimated number of patients is 2,600 benign and 320 malignant cases. There is no gender difference, and the estimated age of onset is 40-45 years, but it is distributed across a wide range of age groups. About 65% are symptomatic (with hypertension), approximately 35% are asymptomatic, and also found as adrenal gland incidental tumors. About 10% were extra-adrenal, bilateral, and malignant, and about 5% had a family history.
^{131}I-MIBG or ^{123}I-MIBG scintigraphy was false-negative in approximately 10%.

1.1.5 **Heredity**

About 30 to 40% of the causes of pheochromocytoma are reported to be hereditary (familial tumor). More than 10 gene alterations associated with pheochromocytoma have now been identified, including genes called SDHB, SDHD, VHL, RET, and NF1. Finding genetic alterations is useful for determining follow-up policies and identifying

the risk of developing the disease in relatives. If a genetic disease such as multiple endocrine neoplasia type II, Von Hippel Linfau disease, or Recklinghausen disease is found, continuous surveillance, such as checking for concurrent diseases other than pheochromocytoma, may be valid. However, identification of genetic alterations may raise additional considerations, such as the possibility of inheritance to family members, and sufficient genetic counseling is required before and after genetic testing regarding findings and concerns.

1.1.6 **Diagnosis**

Catecholamines and their metabolites are measured in blood and urine to assess whether catecholamines are overproduced as a detailed examination of adrenal tumors. In addition, CT, MRI, ^{123}I-MIBG scintigraphy, FDG-PET-CT, and octreotide scintigraphy are performed to evaluate the location and spread of the tumor.

1.1.7 **Treatment**

Treatment requires treatment of the tumor itself and treatment of catecholamine excess symptoms.

The primary treatment for the tumor itself is the surgical removal of the tumor.

Blood pressure and fluid volume control are important for surgery, and systemic management, such as administration of antihypertensive agents (α-blockers) and fluid replacement, is carefully performed. After surgery, regular imaging tests and blood sampling are performed to check for postoperative recurrence.

Anticancer drug treatment may be performed in cases where resection by surgery is difficult, lesions are found in other internal organs, or recurrence occurs after surgery. As for the contents of anticancer drug treatment, the results of CVD therapy (cyclophosphamide, vincristine, dacarbazine) have been reported the most. It has been reported that CVD therapy reduces tumor size in about half of the cases and may lead to improvement in QOL in short to medium term, such as improvement in symptoms. However, data on efficacy are scarce, and careful treatment decisions are advised.

In September 2021, a ^{131}I-MIBG formulation was approved in Japan to indicate "MIBG accumulation-positive unresectable pheochromocytoma/paraganglioma."

For control of symptoms because of overproduction of catecholamines, treat each symptom. For example, antihypertensive drugs are taken internally for hypertension. However, if it is difficult to control with antihypertensive drugs, a catecholamine synthase inhibitor (methyrosine) is used. Phentolamine (a blocker) is also used in severe constipation or intestinal obstruction.

Drug administration, palpation of the abdomen, and invasive examinations/treatments can cause a sudden increase in catecholamine secretion, resulting in a marked increase in blood pressure called a pheochromocytoma crisis, which can be fatal because of organ damage if left untreated. Therefore, caution should be exercised during diagnosis and administration.

1.1.8 Evaluation of malignancy

Considering that PPGLs can be potentially malignant, PPGL was classified as a "malignant tumor" in the 2017 WHO Classification of Endocrine Tumors 2), so careful long-term follow-up is necessary[4]). The presence of metastases to non-chromaffin tissues (bone, lung, liver, lymph nodes) is a positive diagnosis of "malignant." However, evaluating the degree of malignancy, an index of progressive exacerbation, and poor prognosis is important when the metastatic lesion is in the arm stage. The grade evaluation index is shown in Table 1-2.

Table 1-2: PPGL malignancy evaluation index

	Findings suggestive of malignancy/progressive deterioration/poor prognosis
Sex	Male
Age at diagnosis	Middle-aged and older
Tumor size	Large (4-5 cm or more)
Secretory catecholamine pattern	Noradrenergic and dopaminergic
Timing of diagnosis of Metastases	Synchronous with the primary tumor
Tumor localization/type	Abdominal/pelvic paraganglioma
^{18F} -FDG-PET	Accumulated
Histopathological marker	Ki67 staining positive rate>5%
	GAPP 7-10 points
	Negative SDHB staining
Gene marker	SDHB gene mutation positive

1) Clinical findings

Male, age at initial diagnosis (especially 76 years or older), tumor size (6 cm or more) [6)], excess dopamine and norepinephrine [7)], and inoperability of the primary tumor have been reported to be clinical indicators of malignancy [3,8)]. Timing of

diagnosis of primary and metastatic lesions is also important [6,9]). The prognosis of cases diagnosed simultaneously is considered poorer than cases in which metastasis is confirmed 6 months or more after the primary tumor diagnosis and surgery. Multiple logistic regression analysis has reported that the age of 45 years or older and the presence of metastasis at the initial diagnosis are independent determinants of life prognosis10).

Relationships have also been reported between tumor localization, disease type, and malignancy. Bilateral is more malignant than unilateral (21.4% vs. 10.2%), and PGL is more malignant than PCC (40% vs. 10%) [9]). There is also a report[6)] that PGL is the most important prognostic factor. Among PGLs, about 40% of abdominal/pelvic PGLs are malignant, whereas 3-5% of head and neck PGLs are malignant [11)]. Imaging studies (MRI, CT, ^{131}I-MIBG scintigraphy) are evidence of malignancy in non-chromaffin tissues (liver, bone, lung, lymph nodes), but para-aortic ganglion lesions are difficult to distinguish from lymph node metastasis. ^{18}F-FDG-PET has a higher positive rate in metastatic tumors than in benign tumors (82%), and the tumor detection rate is particularly high in SDHB gene mutation-positive PGL12). However, uptake was observed even in benign tumors (58%), limiting specificity.

2) Histopathological marker

Capsular invasion and vascular invasion, which are pathologically malignant findings, are also found in benign PPGL, so they are not useful for differentiation. Ki67 immunostaining, an index of tumor proliferation ability, is considered useful, and a positive cell rate of 3% or more suggests malignancy [13]). Positive frequency is higher in PGL than in PCC. PASS [14)], which diagnoses the degree of malignancy by scoring histological findings, indicates clinical malignancy with a PASS score of 4 or more and benign with a PASS score of less than 4, but there are negative reports regarding its relationship with prognosis[15)]. Kimura et al. [16)], [17)] reported that the degree of differentiation by scoring scale (GAPP) correlated with a 5-year survival rate and prognosis.

3) Gene markers

It has been reported that 20-30% of sporadic PPGLs are hereditary with a genetic mutation. SDHB gene mutation is particularly closely associated with malignancy. The frequency of malignancy in SDHB gene mutation-positive cases is reported to

be 50-90% [18], and 5-50% of malignant PPGL are SDHB gene mutation-positive and have a poor prognosis.

4) Predictive method by a combination of biomarkers
It has been reported that it is possible to semi-quantitatively predict the degree of malignancy through the scoring of multiple markers, including findings such as (1) tumor system of the primary tumor, (2) adrenal or extra-adrenal, (3) secretory catecholamine type (adrenergic or noradrenergic type), (4) presence or absence of invasion into the tumor capsule, (5) presence or absence of SDHB gene mutation, and (6) presence or absence of overexpression of human eGFR type 2 (ERBB-2), a receptor tyrosine kinase

1.1.9 **Problems with current treatment**

According to the pheochromocytoma/paraganglioma, clinical practice guidelines 2018[20], tumor resection by surgical therapy is the first choice (1A). Adequate preoperative treatment with an α1 blocker and strict intraoperative management by an experienced anesthesiologist is essential (1B). Furthermore, always keeping in mind the possibility of malignancy, great care must be taken not to damage the tumor capsule (2C). Laparoscopic adrenalectomy is the standard surgical procedure for smaller pheochromocytomas (1B), but laparotomy is preferred in cases considered to be aggressive (2C). Primary tumor resection is recommended even if curative resection is impossible (2C). Partial adrenalectomy is recommended for less malignant familial pheochromocytoma/paraganglioma and cases after contralateral adrenalectomy (2C).

CVD therapy is the first choice for malignant pheochromocytoma/paraganglioma because it is relatively easy to implement and has mild to moderate side effects (2C). The reduction in tumor volume and catecholamine excess of CVD treatment (complete and partial responses) is 40-50%. CVD treatment prolongs progression-free survival (PFS) in malignant pheochromocytoma/paraganglioma, but there is no clear evidence that it improves life prognosis.

However, the guidelines for malignant pheochromocytoma/paraganglioma in which ^{123}I-MIBG accumulates are as follows. ^{131}I-MIBG treatment is indicated (1B). ^{131}I-MIBG therapy for tumor shrinkage is useful even at low doses (~7.4 GBq), but moderate or higher doses (~7.4 GBq) are more desirable (2C). Multiple doses of ^{131}I-MIBG treatment with a low dose (~7.4 GBq) that can be performed in Japan improves the response rate (2C). Low-dose ^{131}I-MIBG treatment is useful in suppressing excess

catecholamines (2C). ^{131}I-MIBG treatment elicited ≤Grade 2 hematologic toxicity in 10%–20% of cases at low doses, < Grade 3 hematologic toxicity at moderate doses in 50% of cases, and ≥ Grade 3hematologic toxicity at high doses (14.8 GBq~) in ≥80% cases. A thyroid block with inorganic iodine is recommended to reduce hypothyroidism during ^{131}I-MIBG treatment (1B). Results of domestic clinical trials in 17 patients showed that a single dose of 7.4 GBq reduced urinary catecholamines by 23.5%, and the response rate, according to RECIST, was 5.9%.

1.1.10 **About ^{211}At-MABG**

Astatine is a halogen, and its name comes from the Greek word " astatos," which means "unstable." As the name suggests, astatine emits radiation and decays into another element. Among its isotopes, ^{211}At is a nuclide that emits α rays and decays to the stable element lead-207 (^{207}Pb) (half-life 7.2 h). *meta*-astatobenzylguanidine (MABG) is a drug in which ^{211}At is incorporated into a substance called benzylguanidine, which has a chemical structure similar to norepinephrine. MABG is a substrate for the norepinephrine transporter (NET), which takes up norepinephrine, and is taken up by pheochromocytoma cells via NET. In pheochromocytoma cells, NET expression is enhanced compared to normal cells, and a large amount of ^{211}At-MABG is taken into the cells. Because ^{211}At-MABG is stored in pheochromocytoma cells and remains in the cells for a long time, it is expected to be a new treatment option for malignant pheochromocytoma/paraganglioma.

1.2 Non-clinical study results

1.2.1 Pharmacology

^{211}At-MABG has shown a cytotoxic effect on human neuroblastoma cells in vitro. In an in vitro study using rat pheochromocytoma cell PC12, ^{211}At-MABG dose-dependently increased the proportion of cells with DNA double-strand breaks and decreased cell viability.

^{211}At-MABG was administered to mice with subcutaneous transplantation of PC12 cells, ^{211}At-MABG showed high accumulation in PC12 cells and inhibited the proliferation of PC12 cells in a dose-dependent manner.

1.2.2 **Radiopharmacokinetic studies**

^{211}At-MABG in mice showed a tendency similar to that of ^{131}I-MIBG, showing high accumulation in the heart and adrenal glands where NETs are abundant.

Table 1-3 Biodistribution of ^{211}At-MABG and ^{131}I-MIBG in normal mice21)

	% injected dose per gram[a]					
	1 h		4 h		24 h	
tissue	[^{211}At]MABG	[^{131}I]MIBG[b]	[^{211}At]MABG	[^{131}I]MIBG[b]	[^{211}At]MABG	[^{131}I]MIBG[b]
liver	9.17 ± 0.80[c]	7.45 ± 1.06	4.81 ± 0.59	4.08 ± 0.70	1.54 ± 0.11[c]	0.95 ± 0.13
spleen	3.87 ± 0.69	4.55 ± 0.84	3.40 ± 0.42	3.22 ± 0.22	2.65 ± 0.88	1.82 ± 0.14
lungs	7.42 ± 1.84	7.62 ± 1.41	3.84 ± 0.77	3.69 ± 0.87	1.26 ± 0.32	1.10 ± 0.34
heart	20.61 ± 2.71	24.88 ± 3.75	12.97 ± 1.58[c]	16.14 ± 2.60	4.42 ± 0.51	3.75 ± 0.54
kidney	2.83 ± 0.29	2.52 ± 0.18	2.24 ± 0.21[c]	1.62 ± 0.19	1.01 ± 0.05[c]	0.72 ± 0.19
thyroid	2.83 ± 0.61	2.85 ± 0.99	2.24 ± 0.32	2.38 ± 0.50	2.47 ± 0.52	2.60 ± 0.34
blood	0.87 ± 0.05	0.99 ± 0.13	0.52 ± 0.11	0.52 ± 0.08	0.21 ± 0.06[c]	0.10 ± 0.02
adrenals	20.33 ± 5.06	16.85 ± 2.98	16.67 ± 1.91	16.15 ± 4.54	20.79 ± 5.53	26.73 ± 6.10

[a] Mean ± SD (n = 5). [b] No-carrier-added preparation. [c] Uptake of the two agents determined to be statistically significant by a two-sided t test: differences found to be significant are indicated ($p < 0.05$).

In a single-dose intravenous administration pharmacokinetic study in normal mice that we conducted, ^{211}At-MABG was rapidly translocated from the blood into tissues; the blood radioactivity concentration decayed biphasically with a half-life of $T_{1/2}\alpha$ of about 11 min and a half-life of $T_{1/2}\beta$ of 13.6 h. After administration of ^{211}At-MABG, radioactivity distribution in the blood was high in the blood cell fraction, and no free unchanged drug was detected in the plasma fluid fraction after 60 min of administration. Excretion after administration of ^{211}At-MABG showed a tendency similar to that of ^{123}I-MIBG, but 6 h after administration, ^{211}At-MABG tended to be slower than ^{211}At -MABG: 49.3 % ID, ^{123}I-MIBG: 60.9% ID).

1.2.3 **Extended single-dose toxicity study**

An extended single-dose toxicity study of ^{211}At-MABG in normal mice (BALB/c) was performed.

A single dose of 16 MBq/kg (low dose), 48 MBq/kg (medium dose), or 80 MBq/kg (high dose) of ^{211}At-MABG was administered intravenously to 50 mice each (25 males and 25 females). The course was observed for up to 35 d. Among all, there were three death events in high-dose females (N=25); two were euthanized under threat. Performance status scores worsened dose-dependently, and the duration of the worsening was prolonged. Similarly, changes in body weight also decreased in a dose-dependent manner, and recovery was delayed in the high-dose group but recovered by Day 35.

Organ appearance and histopathological examination showed an increase in the frequency of abnormal findings such as adrenal redness/edema, hepatocyte atrophy/inflammatory cell infiltration, and gastrointestinal mucosal atrophy, considered highly sensitive to radiation. Abnormal findings, such as a decrease in hematopoietic cells and hematopoietic tissue, were observed, but they recovered or tended to recover

by Day 35. Concomitant changes in related hematological and blood biochemical test items were noted but resolved by Day 35.

As described above, three cases of death (two cases of urgency euthanasia) were observed in the high-dose group. However, no other irreversible toxicity was observed, and the severely toxic dose in 10% of animals (STD10) was considered to be ≥80 MBq/kg.

In addition, in observing the general condition, the central nervous system, respiratory system, and circulatory system were set as important observation items. The effects on the organ systems that have an important influence on life support were determined.

1.3 Clinical trial results

The ^{211}At-MABG injection has not been clinically tested.

1.4 Potential risk-benefit assessment

Clinical trials of ^{211}At-MABG injection have not been conducted. However, clinical effects such as tumor reduction effect and reduction of catecholamines are expected from non-clinical studies of this drug and clinical trial results of ^{131}I-MIBG, a similar drug that emits β-rays. ^{211}At is an α-emitting nuclide, and because α rays have lower penetrability and a shorter half-life than β rays, there is no need to isolate patients treated with this drug for long periods in an isotope treatment room. When β-ray agents are used, a large amount of radioactivity is administered, and γ-rays are also emitted, making it difficult to respond to emergencies.

Possible side effects include myelosuppression, gastrointestinal toxicity, liver damage, and crisis because of catecholamine release.

For patients that will participate in this study, who have no other appropriate treatment, in light of the dosage we will use based on 1/10 of the human-equivalent dose of the mouse STD10, we do not believe that the risks greatly outweigh the benefits.

2 Purpose of the study

2.1 Purpose of the study

^{211}At-MABG bound with 211At, an α-emitting nuclide, will be administered to pheochromocytoma/paraganglioma patients, and the MTD and RD are determined from the tolerability. We will also examine radiopharmacokinetics, safety, and exploratory efficacy.

2.2 Type of trial

Type of study: Exploratory study
Clinical Trial Phase: Phase I

3 Trial planning

3.1 Study design

This is a single-arm, open-label trial evaluating the tolerability of a single intravenous dose of 211At-MABG in patients with pheochromocytoma/paraganglioma. The purpose is to determine the MTD and RD of this drug. The dose of this drug will be Cohort 1 (0.65 MBq/kg), Cohort 2 (1.3 MBq/kg), and Cohort 3 (2.6 MBq/kg), starting with Cohort 1 and investigating RD according to a 3+3 design.

3.2 Trial period

The trial period for each cohort will be from the date of obtaining informed consent to 12 weeks after administration.

3.2.1 Study treatment period

The treatment period was from the day of administration of the study drug to 12 weeks after administration.

3.3 Investigation of dosage and administration and definition of dose-limiting toxicity (DLT)

3.3.1 Definition of dosage and administration

Patients will receive a single slow intravenous (bolus) dose of the study drug. The dose and dose escalation schedule for each cohort are Table 3: Dosage per levelTable 3-1. Dose escalation will follow a common 3+3 design. Starting with cohort 1, the planned number of cases in each cohort is three to six, and the addition of cases will be stopped when two cases of DLT occur. Fig. 3-1 shows the dose escalation method. If the number of cases falls below 2/3 or 2/6 at the dose level, up to six additional cases will be examined at one lower level.

Table 3: Dosage per level

Cohort	Dose level	Dose	Planned number of cases
Cohort 1	Level 1	0.65MBq/ kg	3-6 cases
Cohort 2	Level 2	1.3MBq/ kg	3-6 cases

Cohort 3	Level 3	2.6MBq/ kg	3-6 cases

Figure 3: Dose escalation method

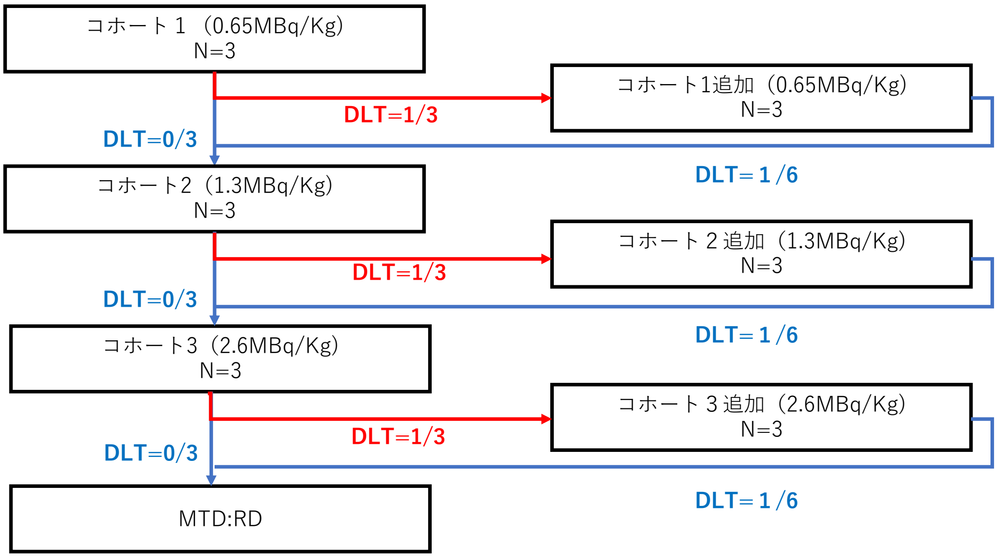

[Rationale]
Initial dose: Non-clinical results showed severe toxicity in 3 of 50 mice in the 80 MBq/kg group. Therefore, the STD10 is considered 80MBq/kg, and the initial dose is 1/10 of the rodent STD10 according to the "Revision of the Guidelines for Clinical Evaluation of Anticancer Drugs" (2021). Therefore, the human-equivalent dose of 8 MBq/kg (in mice) will be used as the initial dose in the clinical trial.
When converted to a human-equivalent dose, this is 0.65 MBq/kg, so the common ratio is 1:2:4, and the dose levels will be as follows: dose level 1: 0.65 MBq/kg, dose level 2: 1.3 MBq/kg, dose level 3: 2.6 MBq/kg.

3.4 Definition of DLT evaluation period shall be from study drug administration to Day 43.

No further subjects will be enrolled until the DLT adjudication period is over.
DLT includes CTCAE Grade 4 hematological toxicity and Grade ≥3 non-hematological toxicity and febrile neutropenia, anemia requiring red blood cell transfusion, and thrombocytopenia requiring platelet transfusion.
However, the following adverse events are excluded.

- Loss of appetite or fatigue.

- Grade 3 nausea or vomiting not requiring gavage or TPN, and Grade 3 diarrhea without prolonged hospitalization. However, the event must be manageable to Grade 2 or less within 7 d after onset with standard antiemetics or antidiarrheals used at the package insert dosage.
- Grade 3 infection.

The final DLT will be determined after consultation between the investigator and subinvestigator for each subject.

[Rationale]
An extended single-dose toxicity test in mice showed marked deterioration of general condition from Day 5 to Day 13, and euthanasia and death were observed on Day 8 and Day 9. A blood test showed the lowest white blood cell count on Day 5, and it has since recovered. In the pathological examination on Day 35, the disorder of the small intestinal mucosa observed on Days 5 and 14 was not observed on Day 35. Based on the above findings, we considered that 35 d would be sufficient for determining DLT in humans, but we conservatively decided to observe for 42 d.

3.5 DLT evaluation procedure in each cohort

1) From the first case of each cohort when DLT appears, report the details to the Efficacy and Safety Evaluation Committee (hereinafter referred to as the committee) and hold a committee meeting.
2) The committee will consider the necessity of measures such as discontinuation or interruption of the clinical trial. The investigator will refer to the results of the review by the committee and decide on future measures.
3) At the end of the DLT evaluation period for the 3rd or 6th subject in each cohort, an efficacy/safety evaluation committee will convene to consider the necessity of measures such as discontinuation or interruption of this study. At that time, all available data will be confirmed, and the investigator will decide whether to proceed to the next cohort, referring to the results of the review by the Efficacy and Safety Evaluation Committee.

Furthermore, after determining DLT, enrollment of new subjects and administration of the study drug to subjects during the treatment period will be terminated until future measures are decided.

3.6 Endpoints

3.6.1 Primary endpoint

The primary endpoint is the presence or absence of DLT to determine the optimal dose for single administration of the study drug. DLT is defined as CTCAE Grade 4 hematologic toxicity and Grade 3 or higher non-hematological toxicity (For details, see 3.4 Definition of DLT). **(**reference).

3.6.2 Secondary endpoints

1) Radiopharmacokinetics (RPK)
   To measure changes in plasma concentration after administration of an investigational drug to patients. Blood will be collected according to the following schedule, and the geometric mean of C_{max}, AUC, $t_{1/2}$, V_{ss}, and CL will be calculated as pharmacokinetic parameters in each cohort. The details of the pharmacokinetic analysis shall be described in the separate pharmacokinetic analysis plan.

2) Urinary radioactivity excretion rate
   Urinary excretion of radioactivity up to 24 h after administration in all cases, and each cohort will be calculated chronologically.

3) Urinary catecholamine response rate
   Patients with urinary catecholamineuria (adrenaline, noradrenaline, metanephrine, normetanephrine) ≥3 times the upper limit of normal at screening are eligible. For all urinary catecholamines at ≥3 times the upper limit of normal at screening, the response is determined when the best overall response of these catecholamines achieves CR (decrease in values of all urinary catecholamines subject to evaluation of efficacy to within the standard values) or PR (decrease of more than 50% relative to baseline in all urinary catecholamine levels included in the efficacy assessment).

4) Overall response rate (ORR)
   ORR will be assessed according to Response Evaluation Criteria in solid tumors (RECIST ver. 1.1) for tumors. In addition, CR and PR will not be confirmed at the time of judgment.

5) PFS

After carrying out a primary objective evaluation of the tumor based on RECIST ver. 1.1, this value will be defined as the time from the date of enrollment to death or progression (including clinical progression), whichever comes first.

6) ^{123}I-MIBG scintigraphy on reducing tumor accumulation
   A comprehensive evaluation of scintigraphy based on overall judgment effect is performed according to the procedure manual for image evaluation.
7) Quality of life
   Measure EORTC QLQ-C30, EQ-5D-5L.

3.7 Scheduled clinical trial period

April 2022 to March 2024.

3.8 Number of cases

The maximum number of cases is 18 (See " 3.3.1Definition of dosage and administration).

4 Pretreatment, concomitant drug/concomitant therapy, post-treatment

4.1 Pretreatment

Treatment for PPGL received before participation in this study is considered prior treatment. In addition, when enrolling in this study, confirm that prior treatment does not affect PPGL (the test value criteria shown in the selection criteria are met).

4.2 Concomitant drug/therapy

All concomitant drugs (including Chinese herbal medicines and over-the-counter drugs) used from 30 d before the case registration date to 12 weeks after administration and all concomitant therapies performed during the same period are considered concomitant drugs and concomitant therapies. However, physiological saline for dilution is excluded.

4.2.1 Prohibited concomitant drugs/therapies

From the point at which informed consent is obtained and until the confirmation of PD or clinical deterioration, the use of the following drugs and treatments considered to affect the efficacy and safety evaluation of this trial is prohibited.

1) Surgical therapy
2) Chemical treatment
3) Granulocyte colony-stimulating factor (G-CSF)

* If you have a history of use, it must be at least 8 d since the last use.

4) Hormone therapy
5) Antibody therapy
6) Radiation therapy (including nuclear medicine treatment)
7) Other antitumor therapies (including so-called immunotherapy)
8) Continuous systemic administration of corticosteroids (>10 mg/d equivalent to prednisone)
9) Systemic administration of immunosuppressants or immunostimulants
10) Ingestion of health food (e.g., Agaric) for the antitumor effect
11) Topical use of corticosteroids at study drug administration sites
12) Antiarrhythmic drugs (ventricular arrhythmias)
13) Sympatholytics (αβ blockers, α blockers, adrenergic neuroleptics)
14) Calcium antagonists: amlodipine, diltiazem
15) Sympathomimetics (inotropic, vasoconstrictive, β_2-agonist)
16) Adrenergic receptor agonist
17) Antidepressants
18) Antipsychotics (neuroleptics)
19) Central nervous system stimulants
20) Blood transfusion before study drug administration

* If you have a history of blood transfusion, at least 29 d have passed since the last blood transfusion.

4.2.2 **Precautions for concomitant use**

Although there are no specific rules, if a new concomitant drug is administered, the particular name of the drug, the date of administration, and the reason for administration should be described in the case report form.

4.2.3 **Concomitant medications and recommended supportive care**

After enrollment, subjects will receive 50 mg/d of potassium iodide orally from 1 to 3 d (at least 24 h before) to 7 d after administration of the study drug as a thyroid block. To prevent vomiting, they will receive a 5-HT3 receptor antagonist immediately before administering the study drug.

4.2.4 **Post-treatment**

There are no restrictions on follow-up treatment after PD or clinical deterioration has been confirmed.

5 Preparation and revision of consent/explanatory documents for subjects

The investigator will prepare the explanatory document/consent document in accordance with the GCP ministerial ordinance and operating procedures. However, descriptions that intentionally guide the subject must not be made. In addition, the prepared explanatory document and consent form shall be approved by the Institutional Review Board and the head of the medical institution before the start of the clinical trial.

If the investigator obtains new findings that affect the subject's consent after the start of the trial and determines that it is necessary to revise the explanatory document/consent document, revise it and notify the clinical trial review board and the medical institution in advance. Get approval from the head. New knowledge affecting subject consent refers to, for example, information on new adverse events related to the investigational drug or information on developing new treatments for the target disease.

6 Target subjects

6.1 Target subjects

Patients with pheochromocytoma/paraganglioma who meet all the "6.2 Inclusion Criteria" and do not meet any of the " 6.3Exclusion criteria" are eligible. Subject gender does not matter.

6.2 Selection criteria

All of the following conditions shall be met.

1) Patients from whom written consent can be obtained.
2) Patients with histologically or clinically diagnosed pheochromocytoma, paraganglioma, malignant pheochromocytoma, or malignant paraganglioma
   * Diagnosis should be made in accordance with the “2018 Clinical Practice Guidelines for Pheochromocytoma and Paraganglioma.”
3) Patients diagnosed with pheochromocytoma are defined below (refractory pheochromocytoma is defined as those who satisfy any of the following ① to ③ and cannot undergo surgical resection or radical external irradiation).
   ① Pheochromocytoma/paraganglioma with an extensive local extension of the primary tumor at first presentation
   ② Malignant pheochromocytoma/malignant paraganglioma with distant metastasis at first presentation
   ③ Pheochromocytoma/paraganglioma with local recurrence or distant metastasis despite surgical resection

4) Patients aged 20 years or older at the time of informed consent.
5) Patients with an ECOG Performance status (PS) of 0-2.
6) ^{123}I-MIBG aggregation positive as determined by scintigraphy at the screening in one or more target lesions confirmed by CT imaging.
7) Patients who meet all of the following criteria with test values at screening.
   ① bone marrow function
      (1) ≥ 3,000/μL without administration of granulocyte colony-stimulating factor (G-CSF)
      (2) Non-transfusion hemoglobin ≥ 9.0 g/dL
      (3) Non-transfused platelet count ≥ 10 x 104 / mm3 (μ L)
   ② Renal function
      (1) Estimated glomerular filtration rate (eGFR) ≥ 30 mL/min/ 1.73m^2
   ③ Liver function (JSCC standardized method)
      (1) AST ≦ 90U / L
      (2) ALT ≤ 126 U/L (male), ALT ≤ 69 U/L (female)
      (3) LDH < 666 U/L
   ④ heart function
      (1) NYHA Functional class: I or lower
   ⑤ Diabetes/endocrine disease
      (1) HbA1c<8.0% (NGSP value)
   ⑥ breathing condition
      (1) Atmospheric blood oxygen saturation (SpO2) ≥ 96%
8) Patients are expected to survive for 3 months or longer.
9) Patients who are expected to be independent in eating, excretion, and sleeping during the nuclear medicine treatment hospital isolation period.
10) Patients for whom standard therapy (CVD therapy or 131I-MIBG therapy) failed or that have no other appropriate therapy.

[Rationale]

1) To comply with GCP ordinances and allow patients to decide whether to participate in clinical trials freely.

2),3) Because the target disease is pheochromocytoma/paraganglioma. (to clarify the disease to be tested).

4) Subjects must be 20 years or older to obtain their consent.

5) to 10) Because these factors may affect test results.

6.3 Exclusion criteria

Exclude patients who meet any of the following criteria:

1) Patients with active multiple cancer
   Active multiple cancers here refer to synchronous multiple cancers and metachronous multiple cancers with a disease-free interval of 5 years or less. However, lesions equivalent to carcinoma in situ or carcinoma in situ that are judged to be cured by local treatment and have a disease-free period of more than 1 year after cure are not included in active multiple cancers. In addition, the following cancers associated with familial pheochromocytoma are not included in active double/multiple cancers.
   (1) Medullary thyroid carcinoma in multiple endocrine neoplasia type 2 (MEN2)
   (2) Retinal hemangioblastoma in VHL
   (3) Neurofibromas in neurofibromatosis type 1 (NF1)
2) Patients who cannot stop the dosage of a drug that suppresses the accumulation of MABG for a predetermined period
3) Patients unable to stop taking α-methylparatyrosine for a prescribed period
4) Patients who have undergone surgery, CVD therapy, catheter hepatic artery embolization for liver metastasis, or radiotherapy within 8 weeks before enrollment
5) Patients who have received MIBG treatment within 12 weeks before enrollment
6) Patients who have developed Grade 2 or higher non-hematological toxicity during or after prior treatment, for which a causal relationship to treatment cannot be ruled out, and who require treatment during the study period
7) Patients who have any of the following infections and require medical treatment during the study period
   (1) Hepatitis B virus infection
   (2) Hepatitis C virus infection
   (3) HIV infection
   (4) Other infectious diseases requiring systemic treatment
8) Patients with a disease that requires continuous systemic administration of adrenocortical hormone (prednisone or prednisolone equivalent dose of 10 mg/d or more) or other immunosuppressants and that requires treatment during the study period
9) Patients with a history of uncontrolled catecholamine seizures
10) Patients with a history of fatal arrhythmia or cardiac arrest
11) Patients with uncontrolled symptomatic arrhythmia, thyroid dysfunction, respiratory disease, and pleural and ascites

12) Patients with coronary artery disease, arrhythmia requiring treatment with amiodarone, severe valvular disease, aortic disease, and bleeding tendency
13) Patients that are pregnant (even if it is determined that there is a possibility of pregnancy by a doctor's interview, the patient will be excluded from this study), within 28 d after giving birth, or breast-feeding (including women who have temporarily stopped breast-feeding)
14) Female patients of childbearing potential or male patients with partners of childbearing potential who are unable to agree to contraception for 6 months after drug treatment (any 2 of the following: contraception methods include latex condoms (used by men), oral contraceptives, intrauterine device (IUD) (progesterone-free T type) used in combination; or tubal ligation and vasectomy)
15) Patients participating in other clinical trials within 3 months before the consent date
16) Other patients who are judged by the investigator or subinvestigator to be unsuitable for this study

[Rationale]
1) to 12) To appropriately evaluate test results.
13), 14) The hazards of this drug to reproductive functions and its transferability into breast milk and fetal toxicity have not been investigated.
15), 16) To appropriately evaluate test results.

7 Obtaining consent

Subjects' voluntary consent will be obtained in writing by the following procedure.

7.1 Before registration

1) The investigator (subinvestigator) must comply with "5Preparation and revision of consent/explanatory documents for subjects" before allowing subject candidates who are considered to be eligible for enrollment to participate in the clinical trial (performing all medical actions for the clinical trial). Provide sufficient explanation using the prescribed consent document and other explanatory documents. In addition, if necessary, the clinical trial collaborators can also provide supplementary explanations.
2) Before obtaining consent, the investigator (subinvestigator) will allow the prospective subject to ask questions and have sufficient time to decide whether to participate in the study.
3) The investigator (subinvestigator) and clinical trial collaborator provide answers to all questions from the candidate subject to the satisfaction of the subject candidate.

4) If the candidate subject agrees to participate in the trial, the investigator (subinvestigator) who provided the explanation and the subject sign and date the consent form. If the clinical trial collaborator provides a supplementary explanation, the clinical trial collaborator shall also sign and enter the date.
5) The investigator (subinvestigator) will hand over a copy of the consent form and the explanatory document to the subject before the subject participates in the trial, and the investigator will keep the original at the study site.

7.2 When obtaining information that affects the subject's will

Suppose the investigator (subinvestigator) obtains the information that may affect the subject's intention to continue participating in the study. In that case, the investigator (subinvestigator) shall promptly inform the subject participating in the study of the information and inform the subject of the intention to continue the study. Confirm and record.

7.3 When consent documents and other explanatory documents are revised

1) When the investigator revises the informed consent form and other explanatory documents, the revised details shall be promptly communicated to the subjects participating in the clinical trial, and their intention to continue the trial shall be confirmed and documented.
2) Subject consent will be obtained again according to Section 7.1 Pre-Enrollment using the revised Institutional Review Board-approved informed consent form and other explanatory documentation.
   During revising the informed consent form and other explanatory documents (excluding minor revisions), consent acquisition for new subject registration will be temporarily terminated.

7.4 Subjects visiting other departments and hospitals

The investigator (subinvestigator) will confirm whether the subject has received consent from another department or hospital. If the subject is undergoing a medical examination, the subject's consent must be obtained, and the physician in charge of the other department or hospital undergoing the examination must be notified that the subject will participate in this clinical trial.

In addition, if the subject is visiting from a distant place, a family doctor who can follow-up in the vicinity will be decided in advance with the consent of the subject, and the investigator (subinvestigator) will contact the doctor in advance and Share

information about a subject's clinical trial. The investigator (subinvestigator) will collect sufficient information when a primary care physician examines the subject during the study period.

8 Enrollment of subjects

8.1 Procedure for case registration

1) The investigator (subinvestigator) and clinical trial collaborators fill in the "Case Registration Form" with information on subjects determined to be eligible and obtain consent.
2) The investigator will determine eligibility based on the contents of the "case registration form" and, if determined to be eligible, will register the case.
3) The investigator (subinvestigator) or clinical trial collaborator shall immediately start the treatment specified in this clinical trial after confirming the completion of case registration. If the subject is ineligible, explain to the subject that they cannot be enrolled in this study.

8.2 Additional registration

We plan to enroll 3 to 6 patients in each cohort, but if the enrolled patients discontinue the trial before completing the DLT evaluation period (excluding cases where DLT occurs), we will additionally enroll subjects.

9 Investigation/observation/test, evaluation items, and timing

9.1 Investigation/observation test and timing of implementation

During the clinical trial period, investigations, observations, and examinations will be conducted according to “Table 9-1 List of investigations, observations, and examination items.” The implementation date and results will be described in the case report form. The dates and results of investigations, observations, and tests related to adverse event information should be described in the case report form for tests conducted on days other than those specified in the protocol.

Investigations, observations, and examinations conducted before case registration shall be completed after consent is obtained. However, if there are survey/observation/test results before obtaining consent (within 30 d before obtaining consent) at the prescribed observation time, they can be substituted.

Table 9/observation1test items

Investigations/processes	Before administration				Day of administration			After administration									
	COI	Screening	Registry	within 7 days	Before administration	Administration	After administration	next day	2 days after	3 days after	7 days after	14 days after	4 weeks after	6 weeks after	8 weeks after	12 weeks after	Suspension[*14]
		Day-42 [illegible]	~Day-14	Day-7 ~-1	Day1			Day2	Day3	Day4	Day8	Day15	Day29 ±7	Day43 [illegible]	Day57 ±7	Day85 [illegible]	±14
		hospital ac		Hospital admission										hosp		Hos	入院
Informed consent	●																
Registry			●[*12]														
^{211}At-MABG administration						●											
Potassium Iodide(KI) administration				●[*13]	●[*13]			●[*13]									
5-HT_3 recepter antagonist administration					●												
Subject's background		●															
Concomitant drugs/Comobities			●→													→	●
**General condition**																	
ECOG Performance Status		●		●				●→				●	●	●	●	●	●
Height		●															
Weight		●		●								●	●	●	●	●	●
physical examination findings		●		●	●		1,2,3,4,6 hours after administration	●→				●	●	●	●	●	●
Bitals[*1]		●		●	●		1,2,3,4,6 hours after administration	●→				●	●	●	●	●	●
Blood oxygen saturation level (SpO_2)		●		●	●		1,2,3,4,6 hours after administration	●→				●	●	●	●	●	●
**Blood serum biochemistry test**																	
Hematology test[*2]		●		●	●		1,3,6 hours after administration	●		●	●	●	●	●	●	●	●
Blood biochemistry[*3]		●		●	●		1,3,6 hours after administration	●		●	●	●		●		●	●
Urine[*4]		●		●										●		●	●
Infection test[*5]		●															
Electocardiodiagram (12-lead ECG)		●			●		5 minutes and 1,3,6 hours after administration	●		●	●	●		●		●	●
Cardiac ultrasonography		●												●		●	●
Urine catecolamine[*6]		●												●		●	●
^{123}I-MIBG scintigraphy[*7]		●												●		●	●
^{123}I-MIBG accumulation[*8]		●															
QOL[*9]		●												●		●	●
**Radiopharmacokinetics**																	
Blood radioactivity concentration					●		5, 15 minutes and 1,3,6 hours after administration	24 hours	48 hours	72 hours							
Urine radioactivity concentration					●		during 0-3, 3-6, 6-12 hours after administration	12-24 hour	24-48 hour	48-72 hour							
Computed tomography (CT)[*10]		●												●		●	●
Pregnancy test (hCG) [*11]		●															
Aedverse events			●→													→	●

*[1] Measures systolic blood pressure, diastolic blood pressure, pulse rate, and body temperature. Blood pressure and pulse rate should be measured in a sitting position after at least 5 min of rest.

* [2] Red blood cell count, white blood cell count, white blood cell differential (neutrophils, eosinophils, basophils, lymphocytes, monocytes) hemoglobin, hematocrit, platelet count

*[3] AST, ALT, ALP, γ-GTP, LDH, C -reactive protein (CRP), total protein, albumin, total bilirubin, total cholesterol, BUN, uric acid, sodium, potassium, chloride, calcium, accumulated glomerular filtration (e-GFR), HbA1c, urinary natriuretic peptide, free triiodothyronine (FT3), free thyroxine (FT4), thyroid stimulating hormone (TSH)

*[4] protein, sugar, urobilinogen, occult blood

*[5] HBs antigen, HBs antibody, HBc antibody, HCV antibody (viral load measurement if necessary), HIV antibody. After starting treatment, viral load should be monitored according to the "Guidelines for Hepatitis B Developed by Immunosuppression and Chemotherapy" for HBV.

*[6] Adrenaline, noradrenaline, metanephrine, normetanephrine: Collect urine for 24 h.

* [7] Anterior and posterior full-body Pummer imaging and SPECT imaging.

* [8] I- 123MIBG scintigraphy during screening confirms uptake of I- 123MIBG in one or more target lesions confirmed by CT imaging at screening.

* [9] The survey will be conducted with EORTIC-QLQ-C30 and EQ-5D-5L questionnaires before explaining the imaging results to the subject.

* [10] As a general rule, plain CT is used.

* [11] Only female subjects of childbearing potential (not surgically infertile, premenopausal, not amenorrheic for at least 12 consecutive months for another medical reason).

* [12] Enrollment of subjects also serves as an order for study drugs.

* [13] As a thyroid block, administer potassium iodide 50 mg/d from 1 to 3 d (at least earlier than 24 h) to 7 d after administration of ^{211}At-MABG.

* [14] Will be conducted to whatever extent possible after considering the subject's clinical condition within ±14 d of the day when the discontinuation was decided.

9.2 Obtaining consent

Consent shall be obtained in accordance with "7Obtaining consent" Obtain written informed consent at the latest by the time the pre-registration test is performed. If consent is obtained from the subject, investigations, observations, and examinations conducted as part of routine medical care may be used as investigations, observations, and examinations specified in the protocol. Subjects consent to this by signing a consent form.

9.3 Assigning subject identification code

The investigator (subinvestigator) will assign a subject identification code to all subjects who consent.

9.4 Hospitalization and admission to the nuclear medicine unit

The screening will be done in the hospital. After registration, the patient will be hospitalized from the day before administration and will be admitted to the nuclear medicine treatment room after administration.

Even after leaving the nuclear medicine treatment room, in principle, the patient is hospitalized for follow-up until Day 15. Regarding discharge, if after examination by a doctor, (1) there are no abnormalities in blood tests and the adverse events that occurred after administration of the study drug for which a causal relationship cannot be ruled out have improved to Grade 2 or less. Family members can visit their homes at least once a day. In addition, at the time of discharge, a report of discharge is made by telephone to a medical facility near the home, and a medical information provision form, including emergency contact information is sent. Examinations on Day 43, Day 85, and at the time of discontinuation will be performed at the hospital.

9.5 Surveys, observations, and tests

9.5.1 Subject background

1) Survey/observation/test items

(1) Consent date

Include the date of informed consent on the case report form.

If re-consent is obtained, the date of consent acquisition and the reason for re-consent should be stated in the case report form.

(2) Case number, case registration date

The case number, case registration date, and subject identification code if registration was unsuccessful, as described in the case registration confirmation

form, and the fact that registration is not possible should be entered in the case report form.

(3) Gender, Date of Birth

The sex and date of birth of the subject will be investigated and recorded in the case report form.

(4) Diagnosis Name

The diagnosis name should be recorded on the case report form.

(5) Date

The date of initial diagnosis of pheochromocytoma/paraganglioma should be noted on the case report form.

(6) Prior treatment history

The case report will investigate and document details of prior treatment for pheochromocytoma/paraganglioma and the duration of prior treatment. In addition, the results of the previous treatment should also be described immediately before the consent acquisition date.

(7) Medical history

Determine disease names (symptom names) and durations for the past 3 years from the date of case registration, excluding diseases considered not to affect the evaluation during the clinical trial period, and describe them in the case report form.

However, in the case of malignant tumors, the period does not matter.

(8) Complications

Regardless of the presence or absence of symptoms at the time of patient registration, the name of the disease or complication (symptom name), the presence or absence of concomitant drugs/concomitant therapy, and the duration of illness should be investigated and described in the case report form. However, diseases that are not considered to affect the evaluation during the trial period can be excluded.

2) Investigation/observation/test period

・ Before case registration: (1) to (8)

9.5.2 Concomitant drugs, concomitant therapy, blood transfusion

1) Survey/observation/test items

For all concomitant drugs used from 30 d before the date of enrollment to 12 weeks after administration, determine the name of the drug, dosage, route of administration, period of use, and reason for use, and describe them in the case report form.

For all concomitant therapies performed from 30 d before the date of case registration to 12 weeks after administration, determine the therapy name, frequency, implementation period, and implementation reason, and describe them in the case report form.

For all blood transfusions performed from 30 d before the patient registration date to 12 weeks after administration, describe the type of transfusion product (red blood cell concentrate or platelet concentrate transfusion), dosage units, implementation date, and reason for use in the case report form.

2) Investigation/observation/test period
- From the day of patient registration to 12 weeks after administration
- Withdrawal

9.5.3 **General condition**

1) Survey/test items

PS (Appendix 1), height, weight, subjective and objective findings, and vital signs (systolic and diastolic blood pressure, pulse rate, body temperature) will be investigated and measured, and recorded in the case report form together with the examination date. Blood pressure measurement should be performed in a sitting position throughout the study period.

2) Investigation/test period

(1) PS:
- Before case registration
- Day of hospitalization (Day 7 to Day 1)
- Day 2 to Day 15, Day 29, Day 43, Day 57, and Day 85
- Withdrawal

(2) Height (cm):
- Before case registration

(3) Weight (kg):
- Before case registration
- Day of hospitalization (Day 7 to Day 1)
- Day 15, Day 29, Day 43, Day 57, and Day 85
- Withdrawal

(4) Examination findings:

The investigator or subinvestigator will perform a physical examination to assess the following bodily functions/organs:

General findings, skin, head, eyes, ears, nose, mouth, throat, lungs, cardiovascular, abdomen, urogenital (as needed), lymphatic system, musculoskeletal/limb, and nerve

- Before case registration
- Day of hospitalization (Day 7 to Day 1)
- Before administration
- Immediately after administration, 1, 2, 3, 4, and 6 h later
- Day 2 to Day 15, Day 29, Day 43, Day 57, and Day 85
- Withdrawal

(5) Vital signs (systolic and diastolic blood pressure, pulse, temperature):

- Before case registration
- Day of hospitalization (Day 7 to Day 1)
- Before administration
- Immediately after administration, 1, 2, 3, 4, and 6 h later
- Day 2 to Day 15, Day 29, Day 43, Day 57, and Day 85
- Withdrawal

(6) Blood oxygen saturation (SpO_2)

- Before case registration
- Day of hospitalization (Day 7 to Day 1)
- Before administration
- Immediately after administration, 1, 2, 3, 4, and 6 h later
- Day 2 to Day 15, Day 29, Day 43, Day 57, and Day 85
- Withdrawal

9.5.4 **Clinical examinations**

1) Hematology

(1) Test items

The following should be noted in the case report form along with the examination date: red blood cell count, white blood cell count, differential white blood cell count (neutrophil count, eosinophil count, basophil count, lymphocyte count, monocyte count), hemoglobin value, hematocrit value, and platelet count are checked for abnormal changes.

(2) Test time

- Before case registration
- Day of hospitalization (Day 7 to Day 1)
- Before administration, 1, 3, and 6 h after administration

- Day 2, Day 4, Day 8, Day 15, Day 29, Day 43, Day 57, Day 85
- Withdrawal

2) Blood biochemistry test
   (1) Test item
   Test for AST (GOT), ALT (GPT), ALP, γ-GTP, LDH, CRP, total protein, albumin, total bilirubin, total cholesterol, BUN, uric acid, Na, K, Cl, Ca, e-GFR, HbA1c, BNP, FT3, FT4, and TSH, and record the presence or absence of abnormal changes in the case report form together with the examination date.
   (2) Test time
   - Before case registration
   - Day of hospitalization (Day 7 to Day 1)
   - Before administration, 1, 3, 6 h after administration
   - Day 2, Day 4, Day 8, Day 15, Day 43, Day 85
   - Withdrawal

3) Urinalysis
   (1) Test item
   Test for protein, sugar, urobilinogen, and occult blood, and describe the presence or absence of abnormal changes in the case report form together with the examination date.
   (2) Test time
   - Before case registration
   - Day of hospitalization (Day 7 to Day 1)
   - Day 43 and Day 85
   - Withdrawal

9.5.5 Other tests

1) Infectious disease test
   (1) Test item
   Tests for HBs antigen, HBs antibody, HBc antibody, HCV antibody (viral load measurement if necessary), and HIV antibody will be performed, and the test date will be recorded in the case report form. After starting treatment, viral load should be monitored according to the "Guidelines for Hepatitis B Developed by Immunosuppression and Chemotherapy" for HBV.
   (2) Test time
   - Before case registration

2) ECG (12-lead)

(1) Test item

Measure a 12-lead ECG (3 measurements). Electrocardiograms (3 measurements) are taken continuously at intervals of several minutes after resting in the supine or semi-supine position for 5 min. RR, PR, QT, and QRS intervals are standard ECG parameters. Examine and evaluate the presence/absence of abnormalities, findings, and clinical significance, and record them in the case report form together with the test date.

(2) Test time

- Before case registration
- Before administration, 5 min after administration (± 1 min), 1 (± 10 min), 3 (± 10 min), 6 h after administration (± 10 min)
- Day 2, Day 4, Day 8, Day 15, Day 43, Day 85
- Withdrawal

3) echocardiography

(1) Test item

Examine and evaluate the presence/absence of abnormalities, findings, and clinical significance, and record them in the case report form together with the test date.

(2) Test time

- Before case registration
- Day 43, Day 85
- Withdrawal

4) Urinary catecholamine test

(1) Test item

Collect urine for 24 h, measure urinary adrenaline, noradrenaline, metanephrine, and normetanephrine, and record in the case report form with the examination date.

(2) Test time

- Before case registration
- Day 43, Day 85
- Withdrawal

5) ^{123}I-MIBG scintigraphy

(1) Test item
^{123}I-MIBG and evaluate MIBG accumulation in the lesion by whole-body scintigraphy. The method for evaluating ^{123}I-MIBG scintigraphy uptake will be described in a separate protocol.

(2) Test time
- Before case registration
- Day 43, Day 85
- Withdrawal

6) Quality of life survey

(1) Test item
PROs will be assessed using the EORTC QLQ-C30 and EQ-5D-5L questionnaires and documented on the case report form with examination dates.

(2) Test time
- Before case registration
- Day 43, Day 85
- Withdrawal

7) Radiopharmacokinetics

(1) ^{211}At-MABG, a blood sample for radioactivity concentration will be collected, and the date and time of blood collection will be recorded in the case report form. If the vital sign measurement is scheduled at the same time as the pharmacokinetic blood sampling, perform the vital sign measurement before the radiopharmacokinetic blood sampling. Collected specimens will be sent to the Advanced Clinical Research Center for radioactivity analysis. The details of sample collection, handling, transportation, and analysis are described in separate procedures.

(2) Test time
- Before administration (-30 min), 5 min after administration (±1 min), 15 min after administration (±5 min), 1 h (±5 min), 3 h (±10 min), 6 h (±10 min), After 24 h (±60 min), 48 h (±60 min), 72 h (±60 min)

8) Radiopharmacokinetics (urinary excretion)

(1) ^{211}At-MABG, collect urine at regular intervals and record the date and time of collection in the case report form. Collected specimens will be sent to the Advanced Clinical Research Center for radioactivity analysis. The details of

sample collection, handling, transportation, and analysis are described in separate procedures.

(2) Test time

- Before administration, 0–3, 3–6, 6–12 h, 12–24 h, 24–48 h, and 48–72 h after administration

9.5.6 Tumor-related examination/observation items

1) Tumor evaluation test (in principle, simple CT should be used. However, if the investigator decides to use a contrast medium, follow the package insert.)

(1) Test items

Target and non-target lesions will be assessed using the same modalities as at enrollment. CT of the chest and abdomen is performed to measure the tumor diameter.

After administration, response or progression is classified according to RECIST criteria version 1.1 [23)] (Appendix 2), and the investigator determines tumor response.

All patient files and radiographic images will be available for case report form and source document verification.

(2) Test time

- Before case registration
- Day 43, Day 85
- Withdrawal
- CT examination will be performed as appropriate (including evaluation based on RECIST) if deemed clinically necessary.

9.5.7 Pregnancy tests: only for women of childbearing potential

Women who meet one or more of the following two items are judged to have no possibility of pregnancy and do not require a pregnancy test at the time of the visit.

- History of surgical sterilization (hysterectomy, bilateral tubal ligation, or bilateral oophorectomy)
- More than 1 year has passed since the last menstrual period

However, subjects considered to be in an amenorrheic state caused by drugs should undergo a pregnancy test.

(1) Test item

Blood human chorionic gonadotropin (hCG) concentration will be measured, and the presence or absence of the test, test date, and test results

will be recorded in the case report form. In addition, if a pregnancy test is not performed on a female subject, the reason for non-testing shall be described in the case report form.

(2) Test time

- Before case registration

9.5.8 Administration status of this drug

The presence or absence of administration, the date of administration, and the time of administration should be described in the case report form.

9.5.8.1 Administration status of potassium iodide and 5-HT_3 receptor antagonists

The presence or absence of administration, the date of administration, and the time of administration should be described in the case report form.

9.5.9 Completion and discontinuation

The subject's completion or discontinuation of the trial and the reasons for discontinuation should be described in the case report form.

9.5.10 Status of admission and discharge

Suppose hospitalization other than for the specified examinations and observations is required from the date of informed consent to 12 weeks after administration or until discontinuation of the study. In that case, the period required and the reason for the required hospitalization shall be described in the case report form.

9.6 Evaluations

9.6.1 Efficacy evaluations

The investigator or subinvestigator will evaluate efficacy according to RECIST criteria version 1.1 [23)] (Appendix 2).

1) Response rate (ORR)
   Proportion of subjects with CR or PR at the best overall response.
2) PFS
   Defined as the time from registration to progression (including relapse/relapse) or death for any reason.
3) Urinary catecholamine response rate.
   For all urinary catecholamines (adrenaline, noradrenaline, metanephrine, normetanephrine) with ≥3 times the upper limit of normal, the response is

determined when the best overall response of the urinary catecholamines ≥ 3 times the upper limit of normal achieves CR (decrease in values of all urinary catecholamines subject to evaluation of efficacy to within the standard values) or PR (decrease of more than 50% relative to baseline in all urinary catecholamine levels included in the efficacy assessment).

4) Effect of reducing tumor accumulation by I-123MIBG scintigraphy
Comprehensive evaluation of scintigraphy based on overall judgment effect is performed according to the procedure manual for image evaluation.

5) QOL rating
EORTC QLQ-C30 and EQ-5D-5L questionnaires will be used to assess PROs.

9.6.2 **Safety evaluations**

Any adverse medical events (including abnormal changes in laboratory test values) in subjects administered this drug will be treated as adverse events, and safety will be evaluated.

If multiple symptoms/signs can be explained as one diagnosis (disease), the associated symptoms/signs should not be listed, and in principle, the relevant diagnosis should be used as the name of the adverse event.

<Example> Runny nose, cough, sore throat: If the three symptoms on the left can be explained as a series of symptoms because of "acute upper respiratory tract inflammation" → "acute upper respiratory tract inflammation."

9.6.3 **Adverse events**

The investigator or subinvestigator will collect data on adverse events through voluntary complaints, interviews, and physical examinations on the day of the subject's visit (whether or not specified in the study schedule). If the investigator or subinvestigator determines that the collected subjective or objective symptoms correspond to adverse events from a medical point of view, they will 11.2

9.6.4 **Changes in clinical test values**

Items with clinically relevant laboratory values or changes in laboratory values occurring from the start of administration of this drug to 12 weeks after administration will 11Ensuring subject safety

[Rationale]

This item was set as a general safety evaluation item.

10 Discontinuation/withdrawal criteria

If any of the following apply, the investigator (subinvestigator) will promptly notify the subject, immediately discontinue administration of the study drug, and take appropriate measures as necessary. In addition, when an adverse event occurs, tests will be conducted to ensure the safety of the subject.

1) Subject withdraws consent to participate in the study.
2) If disease progression is observed. (The specific reason for discontinuation or termination because of disease progression, such as an exacerbation of diagnostic imaging or clinical exacerbation, should be described in the case report form. However, situations, where there are reasonable grounds for clinical benefit justifying the continuation of the study in the event of progression are excepted. In such cases, ensure the safety of the subject.)
3) When a worsening of the general condition requiring discontinuation of the study is observed.
4) If a serious adverse event is observed and the investigator (subinvestigator) determines that it is desirable to discontinue the study drug.
5) If a patient becomes pregnant, if they fail to use contraception, or if they start fertility treatment.
6) Loss of follow-up or non-visit.
7) When a serious protocol violation becomes apparent.
8) In addition, if the investigator (subinvestigator) determines that it is inappropriate to continue the study.
9) If the investigator discontinues the study.

10.1 Discontinuation/withdrawal procedures

10.1.1 Working with subjects

1) If the investigator or subinvestigator discontinues or terminates the study in the subject during the study period, the investigator or subinvestigator shall promptly explain the matter to the subject and conduct necessary observations, examinations, and evaluations.
2) If the investigator or subinvestigator discontinues or terminates the trial because of a safety issue in the subject, such as worsening of symptoms or an adverse event, the subject will be given appropriate treatment.
3) The reason for discontinuing or terminating the study should be described in the case report form.

10.1.2 **Investigation of subjects who stopped coming to the clinic during the study period**

Subjects who do not visit the clinic during the study period will be followed up. In the follow-up survey, the subjects will be asked, as much as possible, about the reasons for not coming to the hospital, the presence or absence of adverse events using means such as telephone, facsimile, letter, visit, etc., and the results will be described in the case report form.

10.2 Completion and discontinuation by subject

10.2.1 **Example of completion**

Cases that have completed the administration of the study drug and 12-week post-administration observation are treated as completed cases.

10.2.2 **Example of withdrawal**

A "10Discontinuation/withdrawal criteria" and failing to complete the treatment period from Day 1 to Day 85 are considered discontinued cases.

11 Ensuring subject safety

11.1 Definition of (AEs)

11.1.1 **AEs**

An AE is any untoward medical occurrence in a subject, regardless of whether or not it is causally related to the drug, including unintended signs and clinically significant changes in laboratory test values (See severity criteria for items not listed in Table 11- 1 "CTCAE v5.0 Japanese translation JCOG/JSCO version"), including exacerbation of disease, symptoms, and complications.

Regarding AEs, new AEs occurring between obtaining informed consent and up to 12 weeks after administration will be recorded in the case report form. Discontinuation should also be recorded in the case report form. Worsening PPGL will be treated as disease progression under efficacy assessments, not as an AE.

All AEs will be followed until resolution to baseline status (in terms of Grade), clinical stabilization of symptoms if recovery is unlikely, or until the subject is no longer available for follow-up investigation.

When treatment such as examination or surgery is taken, the treatment itself is not treated as an AE, but the disease that led to the treatment is treated as an AE. Regarding the death of a subject, the disease-causing the death of the subject is regarded as an AE.

If the subject's condition is the same as before treatment and the expected effects of this drug are not observed despite the administration of this drug, it will not be regarded as an AE.

This study used the Common Terminology Criteria for Adverse Events v5.0 (CTCAE v5.0 Japanese translation JCOG/JSCO version) as the evaluation criteria for AEs. When the severity worsens, and for items for which criteria are not listed, the severity criteria for items not listed in Table 11-1 “CTCAE v5.0 Japanese translation JCOG/JSCO version” are used. Any worsening of the severity of each event will be treated as an AE and described in the case report form.

Table 111

Severity	Criteria
Grade 1	Mild adverse events<br>Mild; treatment not indicated; abnormal imaging/laboratory findings asymptomatic.
Grade 2	Moderate adverse events<br>Minimal/local/non-invasive treatment required.
Grade 3	Severe adverse events<br>Patients with significant symptoms requiring hospitalization, invasive treatment/interventional radiology/blood transfusion/therapeutic endoscopy/surgery.
Grade 4	Life-threatening or incapacitating/disabling adverse events<br>Including acute and life-threatening metabolic/cardiovascular complications. Requires intensive care or emergency treatment (e.g., emergency interventional radiology/therapeutic endoscopy/surgery).
Grade 5	Death because of adverse events

[Abnormal laboratory test values]

Abnormal laboratory values are defined as values outside the range of reference values at the clinical trial site.

[Laboratory values or fluctuations in laboratory values that should be considered clinically relevant]

For laboratory values or changes in laboratory values that should be considered clinically relevant, first, the laboratory value immediately before administration of this drug is defined as the baseline. Relative to baseline, for the items listed in the criteria of the "CTCAE v5.0 Japanese translation JCOG/JSCO version,"[70] a clinically relevant change refers to cases in which the severity of the criteria has worsened. For items not

listed in the criteria, movement from within the reference value range to outside the reference value range provided the movement represents a worsening test value relative to baseline, indicates a clinically relevant change in test values.

The investigator or subinvestigator should determine whether or not the changes in laboratory values or clinically relevant changes in laboratory values are clinically significant and describe them in the case report form. The reason should be stated in the case report form if they are judged insignificant.

The standard values shall be based on those used at the clinical trial site, and if there are any changes during the clinical trial, the revised standard values shall be used from the time of the change.

11.1.2 **Serious adverse events (SAEs)**

The definitions of SAEs shown below are based on “Handling of Safety Information Obtained During Clinical Trials (Notification No. 227 of the Evaluation and Licensing Division, March 20, 1995)” and ICH-E2A “Clinical Safety Management: Definitions and Standards for Expedited Reporting” (Oct 27, 1994).

A SAE refers to any unfavorable medical event that occurred in a subject between the date of informed consent and 12 weeks after administration, as shown in "Table 11-2: Serious Adverse Events"

Table 11-2: Serious Adverse Events

1) Death event	An event in which the adverse event caused or contributed to death. Death is an outcome of an adverse event that led to death.
2) Life-threatening event	Events that placed the subject at risk of death at the time of the adverse event. However, it does not hypothetically mean that if the event had been more severe, it might have resulted in death.
3) Events requiring hospitalization for treatment or an extension of the period of hospitalization	Events requiring hospitalization for at least 24 h or an extension of the planned hospital stay. However, hospitalizations planned before participation in this study, hospitalizations for social reasons, and hospitalizations normally performed for disease management (including hospitalizations for schedule adjustments and follow-up hospitalizations) fall under this item.

4) Events associated with permanent or marked disability/malfunction	Significant disability or dysfunction that interferes with the subject's daily life.
5) Events that cause congenital anomalies	Events occur in children or fetuses of subjects exposed to the study drug before conception or during pregnancy.
6) Other medically significant events	Any other serious event that does not result in death, life-threatening, or hospitalization but endangers the subject or requires action to prevent the above consequences.

The investigator or subinvestigator should report SAEs newly occurring between the date of consent acquisition and 12 weeks after administration as soon as possible to the directors of participating medical institutions and the Investigational Product Provider following "11.4.5 Expedited Urgent report

11.1.3 **Adverse drug reactions (ADR)**

Adverse drug reactions refer to any adverse and unintended reaction (including laboratory abnormalities) to an administered investigational drug, regardless of dose, i.e., a causal relationship between an investigational drug and an AE. In other words, it refers to reactions for which there is at least a reasonable possibility, and causality cannot be denied.

11.2 Recording of AEs

An AE will be evaluated according to "Table 11 -3 Record of Adverse Events" and described in the case report form.

Table 11-3: Records of Adverse Events

Adverse event name	If multiple symptoms/signs are listed in the Subjective Symptoms/Objective Findings column and can be explained as a single diagnosis (disease), do not list the accompanying individual symptoms/signs, and in principle, enter the name of the diagnosis that corresponds to the event.
onset date	The day on which the symptoms newly appeared or the day on which the deterioration of the symptoms observed before the start of administration was confirmed

Severity	Follow the severity scale for items listed in the Common Terminology Criteria for Adverse Events (CTCAE v5.0 Japanese translation JCOG/JSCO version)22). For items not listed in the Common Terminology Criteria for Adverse Events (CTCAE v5.0 Japanese translation JCOG/JSCO version)[22)], classified into five grades from Grade 1 to 5 according to the severity criteria for items not listed in the Common Terminology Criteria for Adverse Events v5.0 Japanese translation of JCOG/JSCO22) (Table 11-1).	
Criticality	1. Non-critical<br>2. Critical	See 11.1.2 Serious Adverse Events (SAEs)
Treatment	1. No<br>2. Yes	If "2. Yes", describe the action taken
Outcome	1. Recovered<br>2. Improvement<br>3. Unrecovered<br>4. Recovered but with sequelae<br>5. Death<br>6. not clear	State outcome date<br>In the case of "1. Recovered," indicate the time of recovery (d). In the case of "2. Resolved, 3. Not recovered, 4. Recovered but with sequelae," indicate the time or confirmation date (d) of "5. Death." Enter the date of death.
Causal relationship with this drug	1. Not relevant<br>2. Related<br>3. Not clear	1. When a causal relationship with this drug can be denied<br>2. When a causal relationship with this drug cannot be denied<br>3. When causality cannot be determined because of a lack of information

11.3 Predictability of AEs

11.3.1 Unforeseeable AEs

An “unforeseeable AEs” is not described in the investigator brochure. In addition, even if it is described, if the number of occurrences, the frequency of occurrence, the tendency of occurrences, such as the conditions of occurrence, nature, and severity, do not match the description ("interstitial nephritis" for acute renal failure, "fulminant hepatitis" for hepatitis”), such AEs shall be considered “unforeseeable.” Because this study is a first-in-human study, all events are unforeseeable AEs at the start of the study.

11.3.2 **Foreseeable AEs**

“Foreseeable AEs” refers to an AEs that is described in the clinical trial drug brochure and that the number of occurrences, frequency of occurrence, and occurrence trends of occurrence conditions are consistent with the described content. Because this study is the first-in-human study, all events are unpredictable AEs at the start of the study.

11.4 Measures to be taken when AEs occur or when pregnancy is confirmed after administration of this drug

11.4.1 **Protocol deviations or changes**

Investigators or subinvestigators may make protocol deviations or changes to ensure subject safety (“15.3 Protocol Deviations or Changes”).

11.4.2 **Measures when AEs occur**

If an AE occurs, the investigator or subinvestigator will take appropriate measures, such as medical treatment and discontinuation of the administration of this drug to the subject as necessary to ensure safety of the subject. In addition, the subject will be notified if any treatment is required. If the investigator becomes aware of a SAE, they will report it to the investigator within 24 h of becoming aware of it.

11.4.3 **Measures when pregnancy is confirmed**

The investigator or subinvestigator will collect pregnancy information on female subjects and partners of male subjects who become pregnant from the day of dosing (Day 1) to 6 months of dosing.

If between the first day of administration (Day 1) to 6 months of administration, a female subject or a partner of a male subject becomes pregnant, the investigator or subinvestigator should immediately discontinue and withdraw that subject from the study and inform them of the pregnancy. Further, within 24 h of determining pregnancy, they should fill in pregnancy information on the "Fetal Drug Exposure Survey (For Enrollment)" and report to the director of the clinical trial site.

Pregnancy itself is not considered an AE or SAE. However, congenital anomaly, spontaneous abortion, or induced abortion for medical reasons is regarded as a SAE, and according to " 11.4.5 Urgent reporting," it shall be reported to the head of the clinical trial site and the study drug provider.

The investigator or subinvestigator will also conduct a follow-up (usually from the date of delivery to 8 weeks) to ascertain the outcome of the pregnancy. Information on

the maternal and child status will be recorded in the "Fetal drug exposure survey (for follow-up)" and reported to the study site director and the study drug provider within 24 h. Every effort will be made to follow pregnancies until definitive pregnancy outcomes are available.

Furthermore, if a SAE occurs as a result of becoming pregnant 6 months or more after administration, and the investigator or subinvestigator determines that a causal relationship to this drug cannot be denied, it shall be considered a SAE. It will be reported to the directors of the study sites and the study drug provider within 24 h, according to “11.4.5 Expedited Reporting.”

11.4.4 **Evaluation and recording**

The investigator or subinvestigator will document the following for AEs or known pregnancies: findings/symptoms/onset date, severity, presence/absence of treatment (if yes), outcome (recovery time if recovered, and time if symptoms stabilized) in source materials (medical records). Investigators should use MedDRA/J (Medical Dictionary for Regulatory Activities/J, ICH International Pharmaceutical Glossary/Japanese version) for the names of AEs described in case report forms. Select the most appropriate term based on medical judgment and replace it as needed.

11.4.5 **Urgent report**

If the investigator becomes aware of a SAE, they will report it to the investigator within 24 h of becoming aware of it. The investigator will make an urgent report to the head of the study site, regulatory authorities, and the study drug provider.

If the subject is coming from a distance and cannot visit the study site immediately, the subject will be advised to see a predetermined primary care physician. At that time, the investigator will immediately contact the primary care physician to request appropriate treatment and collect the necessary information to evaluate safety information.

The report format and procedures for SAEs follow the “Procedures for Handling Safety Information.”

1) Reporting to the head of the clinical trial site
   If the investigator becomes aware of a SAE or pregnancy (congenital malformation, spontaneous abortion, or medically induced abortion only), they shall report it to the directors of clinical trial sites following the “Procedure for Handling Safety Information.”

2) Reporting to regulatory authorities
   Investigators are subject to reporting stipulated in Article 275-3 of the Enforcement Regulations of the Act on Ensuring Quality, Efficacy and Safety of Pharmaceuticals and Medical Devices (hereinafter referred to as the Pharmaceuticals and Medical Devices Act). If an AE occurs, report it to the authorities within the deadline specified in the relevant enforcement regulations.
3) Report to study drug provider
   If the investigator becomes aware of a SAE or pregnancy (congenital anomaly, spontaneous abortion, or medically induced abortion only), the investigator should follow the “procedures for handling safety information.” to the Investigational Product Provider.

11.4.6 **Providing new information**

If the investigator obtains information from the investigational drug provider that may adversely affect the safety of the subject, affect the conduct of the clinical trial, or change the approval of the clinical trial continuation by the Institutional Review Board. That information shall promptly be provided to the directors of clinical trial sites and all subinvestigators involved in the trial.

11.4.7 **Discontinuation of the entire trial**

The investigator will discontinue the entire clinical trial if necessary because of unavoidable ethical or medical reasons, such as ensuring the safety of the subject (see "16.2 Early termination/discontinuation of the clinical trial").

12 Investigational drug information

12.1 Drug substance

12.1.1 **Common name**

3-astatobenzylguanidine(^{211}At) (*meta* -astatobenzylguanidine (^{211}At))

12.1.2 **Chemical name and structure**

Chemical name: 3-astatobenzylguanidine(^{211}At) (*meta* -astatobenzylguanidine (^{211}At))
Molecular Formula: $C_8H_{10}{}^{211}AtN_3$
Molecular weight: 359.07
Structural formula:

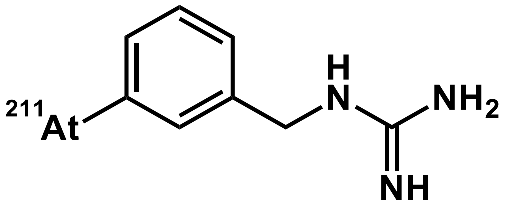

12.2 Investigational drug

Investigational drugs are manufactured in-house by the Advanced Clinical Research Center, Fukushima Medical University Public University Corporation Fukushima Medical University, and quality control is performed.

For details and handling of the investigational drug, refer to the investigator's brochure and the "Procedures for the Management of the Investigational Drug" separately.

12.2.1 **Dosage form**

This drug is a physiological pH intravenous injection containing the active ingredient ^{211}At-MABG. It also contains 3-5% ethanol added during manufacturing and 0.1% sodium ascorbate to inhibit radiolysis. The ingredients and amounts of this drug are shown in Table 12-1.

Table 12 -1: Ingredients in ^{211}At-MABG injection and their amounts

Active ingredient	3-Astatobenzylguanidine (^{211}At) (*meta*-astatobenzylguanidine (^{211}At))		
Stabilizer	JP Sodium Ascorbate	Stabilizer	0.1w/v%
Additive	Sodium chloride (as Japanese Pharmacopoeia saline)	Tonicity agent/solvent	0.9w/v%
Additive	Ethanol	Stabilizer	less than 5 w/v%

12.2.2 **Physicochemical properties**

Appearance/Properties: Colorless to a pale yellow transparent solution; pH: 5.0–9.0.

12.2.3 **Storage method**

The storage method includes room temperature and dark storage (storage in a lead container).

12.2.4 **Date of expiry**

Based on the stability test results, the expiry date was set at 4 h from the time of assay in consideration of the physical half-life (after 4 h, the amount of radioactivity of ^{211}At-MABG decreased to 68%). Moreover, the radiochemical purity was over 90% when stored at room temperature for 20 h.

12.2.5 **Packaging and labeling of investigational drugs**

When the investigator receives the investigational drug from the investigational drug provider, the investigator should request the investigational drug provider to indicate the following on the container of the investigational drug and confirm the contents (because this drug is an in-hospital formulation, it is not pre-packaged).

- Investigational drug name
- A statement that it is for clinical trials
- Investigator's name, title, and address
- Scientific name or identifier
- Serial number
- Storage method, shelf life

13 Investigational drug management

13.1 Delivery, storage, management, and collection of investigational drugs

The investigator shall deliver the Investigational Product received from the Investigational Product manufacturer to the Investigational Product Manager following the "Procedures for Management of Investigational Product."

The Investigational Product Manager shall store and manage the Investigational Product appropriately in accordance with the "Procedures for Investigational Product Management" and prepare the Investigational Product Management Table. In addition, after the completion of the clinical trial, the unused investigational drug will be returned.

13.2 Investigational drug sterility testing

This drug is an in-hospital preparation manufactured on a case-by-case basis, and the half-life of the active ingredient is extremely short. Therefore, the sterility test will be performed after the shipping confirmation. After administration of the study drug, the results of the sterility test will be obtained. If any problem is found in the test results, the study drug manufacturer will promptly notify the investigator of the results in writing. The investigator will carefully observe the study drug-administered subjects,

take adequate measures to maintain their health, and record any findings and actions taken in case report forms.

14 Statistical analysis

This section describes the outline of the statistical analysis plan, and the details are described in the separately specified statistical analysis plan. The version of the statistical analysis plan created when the database was fixed will be the final version, and the final analysis will be performed according to this version.

As appropriate, summary statistics will be calculated for subject demographics, safety, and PK data by cohort or time point. Summary statistics for continuous data include means, medians, standard deviations, and ranges (e.g., Cmax, AUC, geometric mean, and geometric coefficient of variation for PK parameters), and for categorical data, frequencies and proportions will be calculated. The data will also be illustrated as necessary. Assessment of change from baseline to post-treatment or post-treatment percent change relative to baseline is performed in subjects with baseline and post-treatment measurements. Unless otherwise stated, the most recent data measured before the first dose of the study drug will be used as baseline data.

14.1 Number of cases

Three or six cases per cohort, three cohorts, maximum of 18 cases

[Rationale]

In accordance with the "Guidelines for Clinical Evaluation of Anticancer Drugs," the number of subjects was set for a maximum of three doses in a “3+3” design for estimating the maximum tolerated dose (MTD) in phase I clinical trials of antineoplastic agents.

14.2 Analysis target population

14.2.1 Full analysis set (FAS)

Patients enrolled in this study who received at least one dose of the study drug will be considered the FAS. However, ineligible cases after administration and serious protocol violations (not obtaining consent, serious violations of study procedures) are excluded.

14.2.2 Per-protocol set (PPS)

Cases from the full analysis set (FAS) exclude the following cases.

[Excluded cases] (excluded cases conforming to the protocol)

- Examples of Serious Protocol Violations

  If the following protocol violations are observed during the clinical trial period, they will be considered serious protocol violations.

  1) Cases not meeting inclusion criteria or meeting exclusion criteria
  2) Cases that met "10 Discontinuation/withdrawal criteria" during the trial period but were not discontinued/terminated
  3) Cases in which non-compliance with the prescribed dose, administration frequency, or administration method is observed
  4) Patients who received prohibited drugs or received prohibited therapy

14.2.3 **Safety analysis set (SAS)**

The SAS is defined as the group of subjects who received the study drug, excluding subjects with no safety data to be analyzed and GCP violation cases.

14.2.4 **Population for DLT analysis**

Among the subjects who received the investigational drug, the DLT analysis setting excludes those who discontinued the treatment period without completing the DLT evaluation and did not develop DLT from the SAS.

14.2.5 **Target population for radiopharmacokinetic analysis**

Of the subjects who received the study drug, subjects who obtained any plasma radiation concentration measurement results, and subjects who did not violate GCP were included.

14.3 Data handling

The data will be handled as follows at the time of data aggregation and analysis.

- Missing test and measured values are not imputed, and the relevant data are treated as missing. However, if it becomes necessary to evaluate the impact of missing data as a sensitivity analysis, supplementary analysis, such as the imputation of the worst value, will be performed.
- If there are multiple observations within the same period, use the observation date closest to the specified date.

14.4 Analysis methods

14.4.1 **Efficacy analysis**

14.4.1.1 **Analysis of PFS**

For PFS, the survival rate is calculated using the Kaplan-Meier method for the whole and each cohort in FAS and PPS, and the median survival time (MST) and 95% confidence interval (CI) are calculated.

14.4.1.2 **ORR analysis**

The proportion (%) and 95% CI of subjects with CR or PR at the best overall response in FAS and PPS and each cohort will be calculated.

14.4.1.3 **Urinary catecholamine response rate**

In the FAS and PPS, both in the full sets and in each cohort, for all urinary catecholamines (adrenaline, noradrenaline, metanephrine, normetanephrine) with ≥3 times the upper limit of normal at screening, the response is determined when the best overall response of the urinary catecholamines ≥ 3 times the upper limit of normal achieves CR (decrease in values of all urinary catecholamines subject to evaluation of efficacy to within the standard values) or PR (decrease of more than 50% relative to baseline in all urinary catecholamine levels included in the efficacy assessment). Calculate the response rate (%) and 95% CI.

14.4.1.4 **I-123MIBG scintigraphy**

The percentage of subjects and 95% CI for scintigraphy global assessment (s CR, s PR) based on global assessment effect overall and for each cohort will be calculated according to the imaging assessment protocol.

14.4.1.5 **QOL rating**

Overall and in each cohort, EORTC QLQ-C30 is scored for each scale and item, and the mean values are shown in chronological order. Displays the average scores for each EQ-5D-5L scale in chronological order.

14.4.2 **Safety analysis**

14.4.2.1 **AEs**

The incidence of AEs from the first day of administration to Day 43 and up to 12 weeks after the end of administration will be tabulated and summarized by the time of

occurrence, content, severity, and causal relationship for the entire group and each cohort. AE incidence is defined as the percentage of subjects who experienced an AE in SAS. As with the efficacy analysis, the details will be specified separately in a statistical analysis plan. Information necessary for transferring to the next cohort will be calculated as required.

14.4.2.2 **Changes in clinical test values**

Concerning laboratory test values throughout the clinical trial period, charts will be created for the whole safety analysis population and each cohort. Details will be provided in the separately specified statistical analysis plan. Details will be provided in the separately specified statistical analysis plan. Information necessary for transferring to the next cohort will be calculated as required.

14.4.3 **Analysis of DLT**

Data analysis will also be performed during each cohort to determine DLT and transition to the next cohort. We will calculate the event name and occurrence rate of DLT in each cohort.

14.4.4 **Analysis of radiopharmacokinetics**

Data for all subjects for whom serum and urine concentrations are available for each cohort are listed. The cohort will summarize descriptive statistics in tabular form with mean, standard deviation, median, and range. Pharmacokinetic parameters will be listed individually, and descriptive statistics will be summarized by cohort. If a subject's data are insufficient for a proper assessment of pharmacokinetics (e.g., incomplete administration of study drug, missing administration and collection time information, insufficient drug concentration data for calculation of pharmacokinetic parameters), exclude the subject from the pharmacokinetic analysis. All serum and urine concentrations below the lower limit of quantitation or missing values should be indicated in drug concentration data tables and SAS datasets. Concentrations below the lower limit of quantitation are treated as zero in descriptive statistics. The clinical study report shall specify all subjects and samples to be excluded from the analysis. Concentration-time transition mean and median values after the first dose of the study drug are plotted. Individual serum concentration-time courses may also be plotted. Population pharmacokinetic analyzes of serum concentration-time data may be performed using a nonlinear mixed-effects model. Data may be combined with other selected trial data. Details will be provided in the population pharmacokinetic analysis

plan, and the results of the population pharmacokinetic analysis will be provided in a separate report. A dose-response analysis can also be performed. If implemented, describe the details in the analysis plan and report prepared separately.

14.4.5 Intermediate analysis

No interim analysis will be performed in this study.

15 Protocol compliance and deviations/changes and revisions

15.1 Protocol approval and revision

The investigator shall submit the clinical trial protocol to the clinical trial director before the start and obtain the approval of the clinical trial review board and the clinical trial director.
When revising the clinical trial protocol, the investigator will decide on the revision after discussing with the statistical analysis manager, if necessary, the appropriateness of the change and the impact on the evaluation of the clinical trial. The revised protocol must be submitted to the director of the clinical trial site and approved by the Institutional Review Board and the director of the clinical trial site. In the case of significant changes, temporary suspension of patient registration will be considered from the viewpoint of subject protection, and prior deliberations will be conducted by the Efficacy and Safety Evaluation Committee.

After obtaining approval for revisions, the investigator promptly communicates the revisions to the subinvestigators, the outsourced clinical trial management organization, and the clinical trial participants.

15.2 Protocol compliance

The investigator (person who conducts the clinical trial) agrees to the content of the protocol and case reports and certifies that they have agreed to comply with the protocol or stamp or sign and date a document in lieu thereof.

15.3 Protocol deviations or changes

The investigator (subinvestigator) will record all protocol deviations. The investigator should prepare a "Deviation (non-emergency) report (Hospital Form 8-2)" for minor deviant behavior and submit it to the director of the medical institution conducting the clinical trial and the IRB and obtain approval. Of the deviant acts, only those that did not comply with the protocol to avoid immediate danger to the subject or for other

unavoidable medical reasons should prepare a record explaining the reasons and immediately report to the director of the clinical trial site and retain a copy of it.

The investigator or subinvestigator will not deviate from or change the protocol without prior approval of the IRB for compelling medical reasons, such as to avoid immediate danger to the subject. It can be performed. At that time, the investigator should describe the details and reasons for the deviation or change. If revisions to the protocol are appropriate, the proposed revision in the "Emergency Deviation Report (Standardized Form 8)" as soon as possible and submitted to the head of the clinical trial site and the Institutional Review Board for approval.

16 Study completion, discontinuation, and termination

16.1 End of trial

The clinical trial is completed when the clinical trial schedule for all cases is completed, all case report forms are submitted, data are fixed, GCP audits are completed, and the clinical study report (including the QA statement) is completed. The investigator will report the completion of the trial to the subinvestigators, the outsourced trial management organization, the Efficacy and Safety Evaluation Committee, and the parties involved in this trial. In addition, the investigator shall promptly report the completion of the clinical trial to the director of the medical institution conducting the clinical trial in writing.

16.2 Early discontinuation/termination of clinical trials

Because newly obtained information after the start of the clinical trial revealed a serious problem with the continuation of the clinical trial regarding the safety or efficacy of the investigational drug, the Efficacy and Safety Evaluation Committee decided to discontinue the trial early. If there is a proposal to discontinue the trial, or if it is found that the trial site has violated the GCP ordinance or the trial protocol and has interfered with the proper trial, the investigator will decide to discontinue the trial early or terminate it. If the Efficacy and Safety Evaluation Committee's recommendations are not followed, the investigator will report the reason to the Efficacy and Safety Evaluation Committee.

If the investigator decides to discontinue or terminate the trial early, the investigator immediately informs the subinvestigator, the outsourced trial management organization, and the parties concerned with this trial of the reason and subsequent measures and the process after discontinuation. In addition, the investigator shall promptly report early

discontinuation or termination of the trial to the director of the clinical trial site in writing.

If the clinical trial is terminated early or interrupted, regardless of the reason, the investigator (subinvestigator) shall promptly notify the subjects who participated in the clinical trial and take appropriate measures to ensure the safety the subjects.

17 Case report form

17.1 Type

Paper case report forms will be used in this study.

17.2 How to fill out

1) The investigator (subinvestigator) will prepare a case report form according to the "Guidelines for filling in and correcting case report forms."
2) After reviewing and confirming the content, the investigator will handwrite the date (signature date) on the signature page and sign.
3) If correcting the contents of the case report after signing it, sign it again according to the procedure in 2).

17.3 Submission method

The investigator will retain the created case report form and submit a copy of the case report form to the outsourced clinical trial management organization. Corrections after submission are made using the Data Clarification form (DCF). Fill in and submit according to "17.2 How to fill out" and "17.3 How to submit."

18 Monitoring

18.1 Monitoring methods

To confirm that the clinical trial is being conducted safely and appropriately in accordance with the clinical trial protocol and related laws and regulations and that the reliability of the data is sufficiently ensured. Conduct site-visit monitoring for direct access, including collating materials and case reports.

The investigator (subinvestigator) must accept site-visit monitoring by the pre-appointed monitoring director and staff and provide direct access to all clinical trial-related records, such as source documents and case report forms. The monitoring director or contact person will report the discrepancy to the investigator and, if necessary, request correction and resubmission of the case report form.

The timing and details of monitoring will be separately stipulated in the monitoring plan.

18.2 Identification of source documents and other records

Source documents in this clinical trial refer to the following.

- Records of subject consent and subject information
- Medical records, nursing records, case registration forms, case registration confirmation forms, evaluation/examination/observation records stipulated in this clinical trial, and other records used to create case report forms
- Records of treatment prescribed in this study

However, for the following items, when the investigator (subinvestigator) directly enters the case report form, the contents of the form may become the original data.

- Judgment of inclusion/exclusion criteria
- Medical history and severity of complications
- Concomitant drugs/reason for the use of concomitant therapy
- AE severity, severity, outcome, a causal relationship to study drug
- Reason for discontinuation of this study, comments on the outcome
- Other comments and observations

19 Clinical trial quality control and quality assurance

The investigator shall ensure that the clinical trial, data preparation, recording, and reporting are conducted in compliance with the following by implementing the quality control and quality assurance system based on the operating procedures established by the investigator.

- Clinical trial protocol
- Standards stipulated in Article 14, Paragraph 3, and Article 80-2 of the Pharmaceuticals and Medical Devices Law
- Ministerial Ordinance on Standards for Conducting Clinical Trials of Drugs (GCP Ministerial Ordinance)

In addition, the investigator will confirm that the clinical trial is being conducted safely and appropriately in accordance with the protocol and related laws and regulations and that the reliability of the data is sufficiently ensured. For this purpose, the monitoring supervisor or person in charge will visit the facility based on the operating procedure manual.

In addition, the auditor shall evaluate whether the clinical trial complies with the GCP ordinance, clinical trial protocol, and procedures independently and separately

from normal monitoring and clinical trial quality control work., conduct GCP audits from a third-party standpoint as part of quality assurance activities.

19.1 Guaranteed direct access to source documents

The head of the clinical trial site and the investigator shall provide direct access to all trial-related records, including source documents, and cooperate with them during monitoring, audits, and investigations by regulatory authorities and institutional review boards.

19.2 Efficacy and safety evaluation committee

The investigator will establish an Efficacy and Safety Evaluation Committee to monitor whether the clinical trial is being conducted safely and appropriately and to make decisions on DLT and RD based on the collected data. In addition, the Efficacy and Safety Evaluation Committee will be consulted regarding handling registered cases related to the analysis population and responding to the following matters.

- Significant changes to the protocol
- Occurrence of serious AEs
- Matters for which serious problems have been identified through monitoring
- Other matters that the investigator deems necessary for objective deliberation by the Efficacy and Safety Evaluation Committee

20 Storage of records

The investigator shall retain the required documents that should be retained by the person who conducts the clinical trial until the later of the following two dates in accordance with the "Procedures for Record Retention."

1) The day on which 5 years have passed since the date of manufacturing and marketing approval for the investigational drug (the day on which 3 years have passed since the date of the decision to discontinue development if the decision was made to discontinue development). However, if the product must be reexamined after approval under the Pharmaceutical Affairs Law and the period until the reexamination is completed exceeds 5 years, the date on which the reexamination is completed.

2) The date 3 years have passed because the study was discontinued or completed.

The director of the clinical trial site shall retain the required documents to be retained at the clinical trial site until the later of the following dates.

(1) Date of manufacturing and marketing approval for the investigational drug date three years have passed since the date
(2) The date 3 years have passed since the study was discontinued or completed.

21 Monetary payments and insurance

21.1 Costs and subject burdens related to clinical trials

In this clinical trial, the treatment period (from the date of administration of the study drug to 12 weeks after administration and the date of discontinuation) will be covered by medical expenses not covered by insurance.

In addition, this clinical trial will be conducted using the Advanced Clinical Research Center management business subsidy (public funds) as a financial resource. A burden reduction fee of 10,000 yen per night of hospitalization and 10,000 yen per outpatient visit will be paid to the subject.

21.2 Compensation for health damage

The investigator and the medical institution conducting the clinical trial shall be responsible for any health damage to the subjects related to the clinical trial (including those caused when all or part of the work related to the preparation, management, or implementation of the clinical trial is outsourced). As a compensation measure, we will subscribe to insurance, prepare a medical system for the treatment of health damage, and prepare a "procedure for the subject's health damage compensation."

22 Disclosure arrangements

The results obtained in this clinical trial belong to Fukushima Medical University Hospital. The investigator will decide if the results obtained in this trial will be published in a paper or at an academic conference.

23 Ethical and scientific conduct of clinical trials

23.1 Rules to follow

All persons involved in this clinical trial must comply with the Declaration of Helsinki by the World Medical Association (revised in 2013), the Pharmaceuticals and Medical Devices Law, the Enforcement Regulations of the Pharmaceuticals Ministry of Health and Welfare Ordinance No. 28 on May 27), “Ministerial Ordinance for Partial Revision of the Ministerial Ordinance Concerning the Standards for Conducting Clinical Trials of Pharmaceuticals” (December 25, 2020 Ordinance of the Ministry of Health, Labor and Welfare No. 208), Ministerial Ordinance for Partial Revision of the

Enforcement Regulations” (October 5, 2017 Ministry of Health, Labor and Welfare Ordinance No. 106) and the latest related laws and regulations.

23.2 Preparation and revision of explanatory documents and consent documents

The investigator will prepare the explanatory document/consent document in accordance with the GCP ministerial ordinance. However, descriptions that intentionally guide the subject must not be made. In addition, the prepared explanatory document and consent form shall be approved by the Institutional Review Board and the head of the medical institution before the start of the clinical trial.

If the investigator obtains new findings that affect the subject's consent after the start of the trial and determines that it is necessary to revise the explanatory document/consent document, revise it and notify the clinical trial review board and the medical institution in advance. Get approval from the head. New knowledge affecting subject consent refers to, for example, information on new AEs related to the investigational drug, or information on developing new treatments for the target disease.

23.3 Explanation and consent (informed consent)

Before the subject participates in the clinical trial, the investigator (subinvestigator) shall provide a sufficient explanation using an explanatory document and obtain the subject's voluntary written consent to participate in the clinical trial. When obtaining written informed consent, the investigator (subinvestigator) who provided the explanation and the subject should fill in the written consent form with their names and seals or signatures after the subject fully understands the content of the written explanation. Consent forms will be preserved at the clinical trial site, and copies of the explanation and consent forms will be provided to the subject.

Suppose the investigator (subinvestigator) obtains information that may affect the subject's decision to continue participating in the study. In that case, the investigator (subinvestigator) shall immediately provide the subject with the information and record it to ascertain whether the subject will continue participating in the study. In addition, if the investigator revises the explanatory document, the investigator (subinvestigator) will explain again using the revised explanatory document and obtain voluntary written consent from the subject to continue participating in the study.

23.4 Personal information and privacy protection

Persons involved in clinical trials shall comply with applicable laws and ordinances regarding the protection of the personal information of subjects. In addition, the parties concerned recognize that privacy-related information, such as personal information and clinical information obtained in this clinical trial, should be strictly protected and handled carefully based on the principle of respecting the personality of the individual. We will do our utmost to protect information and privacy and strive to do so.

When the investigator (subinvestigator) submits the case registration form and case report form, the subject identification code or registration number will be used, and information that can identify the subject by a person other than the medical institution will not be entered.

Outsourced clinical trial management organizations manage data using subject identification codes or registration numbers. In addition, when the investigator (subinvestigator) publishes the information obtained from the clinical trial, due consideration shall be given so that the subject cannot be identified.

Personal information and clinical information obtained in the course of this clinical trial will be handled in accordance with the "Act on the Protection of Personal Information" (Enforced Act No. 57 of May 30, 2003) and the "Fukushima Prefecture Personal Information Protection Ordinance" (Based on Article 7, enforced on April 1, 2016, hereinafter referred to as the Fukushima Prefectural Ordinance), information must not be disclosed without justifiable reason. In addition, based on Article 10 of the Fukushima Prefectural Ordinance, the same shall apply even after the relevant person has retired.

24 Investigator responsibilities

The main duties of the investigator are described below (in accordance with Articles 42 to 55 of the Ministry of Health, Labor and Welfare Ordinance No. 28 of March 27, 1997, "Ministerial Ordinance Concerning Clinical Trial Practice Standards").

1) Submission of documents to the Institutional Review Board and conduct of the clinical trial in accordance with the instructions and decisions of the director of the medical institution conducting the clinical trial
2) Be able to secure subinvestigators and clinical trial collaborators
   (1) Create a list of tasks to be shared and submit it to the director of the clinical trial medical institution for approval.
   (2) To provide clinical trial information, guidance, and supervision to subinvestigators.

3) Appropriate subject selection based on ethical and scientific considerations and protocol-specified inclusion and exclusion criteria
4) Obtaining written informed consent from subjects to participate in clinical trials
   (1) Working with the study drug provider to prepare informed consent and other explanatory documents
   (2) Appropriately explain to the subject by consent form and other explanatory documents, and deliver those documents to the subject
   (3) Obtain the signature or name and seal of the person who explained the consent document. If there is an assistant explainer, obtain the signature or name and seal of the assistant explainer.
   (4) Provide a copy of the informed consent document to the subject
   (5) When new information that affects the intention of the subject is obtained, the information should be immediately given to the subject.
   (6) If new information is obtained, the explanation/consent form shall be revised as necessary, and the subject's written consent for continued participation in the clinical trial shall be obtained again.
5) Explain the administration method of this drug to the subject and confirm the administration status
6) Responsibility for all clinical trial-related medical decisions for subjects
   (1) If a subject is being treated by another doctor, inform that doctor of the subject's intention to participate in the study.
   (2) Take measures in advance to ensure that appropriate medical care is provided for AEs.
   (3) Subjects should be notified when an AE occurs, and treatment is required.
7) If a subject decides to withdraw from the course or does so, the rights of the subject should be fully respected, and efforts should be made to ascertain the reason for this.
8) Do not deviate from the protocol to avoid immediate danger to the subject, and record any deviations.
9) If there is a deviation from the clinical trial protocol to avoid an immediate danger to the subject, a record should be created and submitted to the director of the clinical trial site and the Institutional Review Board.
10) If a SAEs occurs, promptly report it to the director of the clinical trial site.
11) Report in writing to the head of the study site when the study is terminated (or discontinued)
12) Completing accurate case report forms

(1) Check the case report form prepared by the subinvestigator and confirm the contents
(2) Any changes or corrections made by the subinvestigator should also be reviewed to ensure no problems.
(3) Ensure that case numbers are used to identify subjects

13) Preservation of clinical trial documents or records related to the conduct of the clinical trial in accordance with the instructions of the director of the clinical trial site
14) Accept investigations by monitors, auditors, clinical trial review boards, and regulatory authorities, and provide relevant records such as source documents for direct testing in response to their requests.

25 Clinical trial implementation system

This clinical trial is an investigator-initiated clinical trial conducted at a single facility at Fukushima Medical University.

25.1 Clinical trial medical institution

Fukushima Medical University Hospital
1 Hikarigaoka, Fukushima City, Fukushima Prefecture 960-1295
TEL: 024-547-1252 FAX: 024-548-2735

25.2 Investigator

Department of Nuclear Medicine, Fukushima Medical University Hospital
Dr. Akira Shiga

25.3 Clinical trial/development plan support manager

Fukushima Medical University Medical Research Promotion Center
Masao Kobayakawa

25.4 Investigational drug manufacturer

Fukushima Medical University Fukushima International Medical Science Center
Advanced Clinical Research Center
Professor Kazuhiro Takahashi
1 Hikarigaoka, Fukushima City, Fukushima Prefecture 960-1295
TEL: 024-547-1674 FAX: 024-547-1666

25.5 Chief of statistical analysis

Foundation for Cancer Research Ariake Hospital
Clinical Research Center Planning and Quality Control Department
Deputy Director Naoki Ishizuka
3-8-31 Ariake, Koto Ward, Tokyo 135-8550

25.6 Pharmacokinetic analysis manager

Meiji Pharmaceutical University Laboratory of Pharmacokinetics
Professor Kazuhiko Hanada
2-522-1 Noshio, Kiyose 204-8588 Tokyo Prefecture

25.7 Efficacy and safety evaluation committee

Nagasaki University Atomic Bomb Disease Institute
Atomic Bomb and Hibakusha Medical Department
Department of Radioisotope Diagnostics and Therapeutics
Professor Takashi Kudo

National Institutes for Quantum and Radiological Science and Technology Quantum Life and Medicine Division
Institute for Quantum Medical Science Molecular Imaging Diagnostics and Therapeutic Research Department
General Manager Tatsuya Azuma

Department of Radiology, Kindai University School of Medicine
Professor Makoto Hosono

25.8 Other clinical trial management organizations

The following outsourced clinical trial management organizations are shown in Attachment 1.

- Data management officer
- Monitoring officer
- Audit manager

26 Citations

1) Solcia E, et al. eds. Tumors of Adrenal and Extra-adrenal Paraganglioma. In:WHO Histological Typing of Endocrine Tumors 2nd. IARC Press 2000:38.(I)

2) Lloyd RV, et al. eds.: WHO Classfication of Tumors of Endocrine Organs In: World Health Organization Classfication of Tumors IARC Press 2017:180.1 (I)

3) Hamidi O, et al.: Malignant Pheochromocytoma and Paraganglioma : 272 Patients Over 55 Years. J Clin Endocrinol Metab2017;102:3296-3305.(IV)

4) Lenders JW, et al.: Endocrine Society. Pheochromocytoma and paraganglioma: an endocrine society cliniycal practice guideline. J Clin Endocrinol Medab 2014;99:1915-1942.(VI)

5) Hartley L, et al.: Pheochromocytoma in Queensland 1970-1983.Aust NZ J Surg 1985;55:471-475.(IV)

6) Ayala-Ramirez M, et al.: Clinical risk factors for malignancy and overall survival in patients with pheochromocytomas and sympathetic paragangliomas: primary tumor size and primary tumor location as prognostic indication. J Clin Endocrionol Metab2011; 96(3):717-725.(IV)

7) van der Harst E, et al.: The value of plasma markers for the clinical behavior of phaeochromocytomas.Eur J Endocrinol 2002;147:85-94.(IV)

8) Dhir M, et al.: Clinical Predictors of Malignancy in Patients with Pheochromocytoma and Paraganglioma.Ann Surg Oncol 2017;24:3624-3630.(IV)

9) Goffredo P, et al.: Malignant pheochromocytoma and paraganglioma: a population level analysis of long-term survival over two decades. J Surg Oncol2013;107 : 659-664.(IV)

10) Burnichon N, et al.: Somatic NF1 inactivation is a frequent event in sporadic pheochromocytoma. Hum Mol Genet 2012;21: 5397-5405.(IV)

11) Jafri M, et al.: Evaluation of SDHB, SDHD and VHL gene susceptibility testing in the assessment of indviduals with nonsyndromic phaeochromocytoma, paraganglioma and head and neck paragaglioma. Clin Endocrinol (Oxf) 2013;78:898-906.(IV)

12) Timmeers HJ, et al.: Superiority of fluorodeoxyglucose positron emission tomography to other functional imaging techniques in the evaluation of metastatic SDHB-associated pheochromocytoma and paraganglioma. J Clin Oncol 2007;25:2262-2269.(IV)

13) Nagura S, et al.: Immunohistochemical estimations of growth activity to predict biological behavior of pheochromocytomas. Mod Pathol 1999;12:1107-111.(IV)

14) Thompson LD: Pheochromocytoma of the Adrenal Gland Scaled Score (PASS) to separate benign from malignant neoplasms: a clinicopathologic and immunophenotypic study of 100 cases.Am J Surg Pathol 2004;26:551-566.(IV)

15) Ocal I, et al.: Lack of correlations among histopathological parameters, Ki-67 proliferation index and prognosis in pheochromocytoma patients. Asian Pac J Cancer Prev2014;15:1755.(IV)

16) Kimura N, et al.: Pathological grading for predicting metastasis in phaeochromocytoma and paraganglioma. Phaeochromocytoma Study Group in Japan.Endocr Relat Cancer 2014;21:405-414. (IV)

17) Kimura N, et al.: Histological grading of adrenal and extra-adrenal pheochromocytomas and relationship to prognosis: a clinicopathological analysis of 116 adrenal pheochromocytomas and 30 extra-adrenal sympathetic paragangliomas including 38 malignant tumors. EndocrPathol 2005;16:23 ‑ 32.(IV)

18) Frederieke M, et al.: High frequency of SDHB germaline mutations in patients with malignant catecholamine-producing paragangliomas: implicatopms for genetic testing. J Clin Endocrinol Metab 2006;91:4505-4509.(IV)

19) Zhong X, et al.: Establishment and evaluation of a novel biomarker-based nomogram for malignant phaeochromocytomas and paragangliomas. Clin Endocrinol (Oxf) 2017;87:127 - 135. (IV)

20) The Japanese Endocrine Society, "Survey on the actual situation of malignant pheochromocytoma and development of clinical guidelines" Committee ed.: Clinical practice guidelines for pheochromocytoma and paraganglioma 2018.

21) Vaidyanathan G, Zalutsky MR. 1-(m-[^{211}At]astatobenzyl)guanidine: synthesis via astato demetalation and preliminary in vitro and in vivo evaluation. *Bioconjugate chemistry* 1992; **3** : 499-503.

22) JCOG, JSCO: Common Terminology Criteria for Adverse Events v5.0 (CTCAE v5.0 Japanese translation)

27 Appendix

付録 1： ECOG Performance Status（PS）の評価

（日本語訳：NCI-CTC 日本語訳 JCOG 版 第 2 版，2001 年 より）

出典 Common Toxicity Criteria, Version2.0 Publish Date April 30, 1999

http://ctep.cancer.gov/protocolDevelopment/electronic_applications/docs/ctcv20_4-30-992.pdf

JCOG ホームページ http://www.jcog.jp/

Score	定義
0	全く問題なく活動できる。<br>発病前と同じ日常生活が制限なく行える。
1	肉体的に激しい活動は制限されるが、歩行可能で、軽作業や座っての作業は行うことができる。<br>例：軽い家事、事務作業
2	歩行可能で自分の身の回りのことは全て可能だが作業はできない。<br>日中の 50%以上はベッド外で過ごす。
3	限られた自分の身の回りのことしかできない。<br>日中の 50%以上をベッドか椅子で過ごす。
4	全く動けない。<br>自分の身の回りのことは全くできない。<br>完全にベッドか椅子で過ごす。

Appendix 2: Revised RECIST Guideline version 1.1

New response evaluation criteria in solid tumors: Revised RECIST guideline (version 1.1)

EA Eisenhauer, P. Therasse, J. Bogaerts, LH Schwartz, D. Sargent, R. Ford, J. Dancey, S. Arbuck, S.

Gwyther, M. Mooney, L. Rubinstein, L. Shankar, L. Dodd, R. Kaplan, D. Lacombe, J. Verweij

EUROPEAN JOURNAL OF CANCER 45 (2009) 228–247

(From Japanese translation JCOG version ver.1.0)

Measurable tumor lesions:

Accurate measurements are possible in at least one direction (record the largest diameter (major axis) in the cross-section to be measured),

And larger than at least one of the following:

- 10 mm on CT (CT slice thickness <5 mm)
- 10 mm as measured by caliper for clinical assessment (lesions that the caliper cannot accurately measure are recorded as non-measurable)
-

Measurable malignant lymph nodes:

- 20 mm on chest radiograph. For lymph nodes judged pathologically enlarged and measurable, the short axis diameter (minor axis) evaluated by CT is 15 mm or more (CT slice thickness of 5 mm or less is recommended).

Non-measurable lesions:

- All non-measurable lesions, including small lesions (tumor lesions <10 mm in the major axis or nodal lesions ≥10 mm and <15 mm in the minor axis) and true non-measurable lesions. Lesions considered non-measurable lesions include: leptomeningeal lesions, ascites, pleural or pericardial effusion, inflammatory breast cancer, cutaneous or pulmonary lymphangiosis, abdominal masses or abdominal organ enlargement visible on physical examination but not measurable by reproducible imaging techniques.

Lesion measurement

Record all measurements in the metric system. For clinical evaluation (physical examination), measure with a caliper. All baseline assessments should be performed before and as close to treatment initiation as possible. It must be performed no later than 4 weeks before starting treatment.

Each lesion reported as a target or non-target lesion must be recorded using the same assessment method and technique throughout the baseline and follow-up periods. Imaging-based, rather than clinical, evaluation should always be performed unless a follow-up lesion is not amenable to imaging but is amenable to clinical evaluation.

Recorded at baseline assessment of 'target lesions' and 'non-target lesions'

If there are ≥2 measurable lesions at baseline assessment, up to a total of five lesions (maximum two lesions per organ) representing all invading organs were selected as target lesions, and record the measurements in the evaluation.

Target lesions are selected based on lesion size (lesions with measurable greatest diameter) and should be representative of all invading organs. In addition to this, lesions must be reproducible and repeatable. Sometimes the largest lesion is not suitable for reproducible measurement, so the next largest lesion that allows reproducible measurement should be selected.

Lymph nodes deserve special mention because they are normal anatomical structures that can be imaged without tumor invasion. Pathological nodes defined as measurable and eligible for target lesion selection, as described in Section 3, must have a minor axis of 15 mm or greater on CT. These lymph nodes add only the short axis to the sum of the diameters of the baseline assessment. The minor axis of a lymph node is the diameter commonly used by imaging physicians to determine whether solid tumors have invaded it. Lymph node size is usually reported in two planes (for CT, this is almost always the transverse plane; MRI can be transverse, sagittal, or coronal). The smaller of these measurements is the minor axis. For example, if an abdominal lymph node measures 20 mm × 30 mm, the minor axis is 20 mm and is considered a measurable lymph node lesion. In this example, 20 mm should be recorded as the measurement of lymph node size. All other pathologic adenopathies (minimum diameter ≥10 mm and <15 mm) are non-target lesions. Nodes less than 10 mm in the minor axis at baseline evaluation are nonpathological (non-lesional) and should not be recorded or followed. The sum of the diameters of all target lesions at the baseline evaluation (hereafter referred to as the sum of diameters; long diameter for non-lymph node lesions and short diameter for lymph node lesions) will be calculated and reported as the baseline sum of diameters. As mentioned above, when including the diameter of a lymph node in the sum of diameters, only the minor diameter is added. The baseline sum of diameters is used as a control (standard) for subsequent objective tumor shrinkage efficacy. Other lesions (or sites of involvement) other than target lesions, including lymph node involvement, are non-target lesions recorded at baseline assessment. These non-target lesions do not require measurement and are evaluated as "yes," "no," and rarely for "obvious exacerbation." In addition, multiple non-target lesions in the same organ may be recorded as one lesion on the Response Record Form (e.g., "multiple enlarged pelvic lymph nodes" or "multiple liver metastases").

<u>Evaluation of target lesions</u>

- Complete Response (CR):
  Disappearance of all target lesions.

All nodal lesions selected as the target must be reduced to less than 10 mm in the short axis.

- Partial Response (PR):
  ≥30% reduction in target lesion sum diameter compared to baseline sum diameter.
- Progressive Disease (PD): The sum
  ≥20% increase in target lesion and ≥5 mm absolute increase in total diameter of the target lesion compared to the lowest total diameter during the course diameter (if the baseline sum of diameters is the minimum value during the course, this shall be the minimum sum of diameters).
- Stable Disease (SD):
  No shrinkage corresponding to PR and no increase corresponding to PD relative to the smallest diameter sum over time.

Evaluation of non-target lesions

- CR:
  Disappearance of all non-target lesions and tumor marker levels below the upper limit of normal. All lymph nodes must be less than 10 mm in the minor axis to be considered pathologically enlarged.
- Non-CR/Non-PD (Non-CR/Non-PD):
  One or more residual non-target lesions and/or tumor marker levels above the upper limit of normal.
- PD:
  Obvious exacerbation of pre-existing non-target lesions.

New lesions

If a lesion is identified during a follow-up examination in an organ or site not imaged at the baseline evaluation, it is considered a new lesion and determined as progression. An example is when a subject with trunk disease at baseline had a CT or MRI of the brain during the study and was found to have metastases. Brain metastases in this subject are considered evidence of PD even in the absence of baseline evaluation brain imaging.

Overall effect

Response at each time point: with target lesions (with or without non-target lesions)

Target lesion	Non-target lesion	New lesion	Overall effect
CR	CR	none	CR

CR	Non-CR/non-PD	none	PR
CR	no rating	none	PR
PR	Non-PD or missing evaluation	none	PR
SD	Non-PD or missing evaluation	none	SD
Missing evaluation	Non-PD	none	NE
PD	I don't care	Yes or No	PD
I don't care	PD	Yes or No	PD
I don't care	I don't care	can be	PD
CR: Complete response, PR: Partial response, SD: Stable, PD: Progression, NE: Not evaluable			

Efficacy at each time point: with non-target lesions only

Non-target lesion	New lesion	Overall effect
CR	none	CR
Non-CR/non-PD	none	Non-CR/non-PD*
there is no rating	none	NE
Obvious exacerbation	Yes or No	PD
I don't care	can be	PD
CR: complete response, PD: progression, NE: not evaluable<br>*Several trials are increasingly using SD as an efficacy endpoint, and it is not recommended to apply this category in the absence of measurable disease. Therefore "stable" rather than "stable" for non-target disease. Non-CR/non-PD is preferred		

Determining the best overall response in studies that do not require confirmation of complete or PR:

The best overall response in these trials is defined as the best response across all time points (e.g., SD at first assessment, PR at second assessment, PD at last assessment, the best overall response is PR). If the best overall response is to be SD, the protocol-specified criteria for the minimum duration from baseline must also be met. If the SD is the best response except that the shortest duration criterion is not met, the best overall response depends on subsequent assessments. For example, if the first assessment is SD and the second assessment is PD, and the shortest duration criteria for SD are not met, the best overall response is PD. If similar subjects were lost to follow-up after the initial SD assessment, the best overall response would be 'not evaluable.'
