# Supplementary material for: Evaluation of pharmacokinetics, safety, and efficacy of [211At] meta-astatobenzylguanidine ([211At] MABG) in patients with pheochromocytoma or paraganglioma (PPGL): A study protocol
Source: PLoS One. 2024 May 28;19(5):e0303623. doi: 10.1371/journal.pone.0303623 (PMC11132457; doi:10.1371/journal.pone.0303623)
Supplement: Attachment — Submitted filename: renamed_c01a4.docx [file pone.0303623.s004.docx]

**Responses to Reviewer’s**

**Comment 1.** Does the manuscript provide a valid rationale for the proposed study, with clearly identified and justified research questions?

The research question outlined is expected to address a valid academic problem or topic and contribute to the base of knowledge in the field.

***Response to Comment 1:*** *Thank you for your valuable comment. This study is a first-in-human clinical trial of an anticancer drug. The rationale of the study is only from non-clinical pharmacological and biodistribution studies, which indicate the efficacy profiles for pheochromocytoma cells. They are described in the Introduction section (references: 12–17). We have added that the required toxicity studies were conducted prior to the first dose in humans, in accordance with ICH-M3 and -S9 guidelines (https://www.pmda.go.jp/files/000156321.pdf ). Based on these guidelines, we set the start dose for first administration in human, which is described in the Discussion section.*

**Comment 2.** Is the protocol technically sound and planned in a manner that will lead to a meaningful outcome and allow testing the stated hypotheses?

The manuscript should describe the methods in sufficient detail to prevent undisclosed flexibility in the experimental procedure or analysis pipeline, including sufficient outcome-neutral conditions (e.g. necessary controls, absence of floor or ceiling effects) to test the proposed hypotheses and a statistical power analysis where applicable. As there may be aspects of the methodology and analysis which can only be refined once the work is undertaken, authors should outline potential assumptions and explicitly describe what aspects of the proposed analyses, if any, are exploratory.

***Response to Comment 2:*** *This study was planned as a phase I study of an anticancer drug. The main purpose of a phase I cancer clinical trial is to determine the maximum tolerated dose and recommended dose by elucidating the dose-limiting toxicity in each dose escalation cohort. That is why, in the present study, there were no statistical hypotheses, and no statistical tests were planned. Regarding exploratory efficacy endpoints, such as objective response and progression-free survival, and pharmacological parameters, point estimations of these parameters are calculated.*

**Comment 3.** Is the methodology feasible and described in sufficient detail to allow the work to be replicable?

Descriptions of methods and materials in the protocol should be reported in sufficient detail for another researcher to reproduce all experiments and analyses. The protocol should describe the appropriate controls, sample size calculations, and replication needed to ensure that the data are robust and reproducible.

***Response to Comment 3:*** *Sample size is not calculated statistically, but is traditionally set according to the modified Fibonacci dose escalation (3 + 3 design). In addition, there are usually no controls in phase I cancer clinical trials. Therefore, we have now added an explanation about the modified Fibonacci method in the section describing sample size and statistical analysis. We believe this manuscript ensures reproducibility of study planning and conducting.*

**Comment 4.** Have the authors described where all data underlying the findings will be made available when the study is complete?

The [PLOS Data policy](https://journals.plos.org/plosone/s/materials-and-software-sharing) requires authors to make all data underlying the findings described in their manuscript fully available without restriction, with rare exception, at the time of publication. The data should be provided as part of the manuscript or its supporting information, or deposited to a public repository. For example, in addition to summary statistics, the data points behind means, medians and variance measures should be available. If there are restrictions on publicly sharing data—e.g. participant privacy or use of data from a third party—those must be specified.

***Response to Comment 4****: We have added the following sentence to the data availability subsection in the METADATA: “No datasets were generated or analyzed during the current study. All relevant data from this study will be made available upon study completion.”*

**Comment 5.** Is the manuscript presented in an intelligible fashion and written in standard English?

PLOS ONE does not copyedit accepted manuscripts, so the language in submitted articles must be clear, correct, and unambiguous. Any typographical or grammatical errors should be corrected at revision, so please note any specific errors here.

***Response to Comment 5:*** *We have checked this revised manuscript prior to resubmission, and had it proofread by native English-speaking scientific editors.*

**Comment 6.**

**Comment 6-1:** Please be consistent with the verbal tenses: over the manuscript, you switch from the present to the future tense repeatedly. Since the study is ongoing, you might want to stick to the present tense. Overall, the text reads fine; a review by a native English speaker might be, however, considered.

***Response to Comment 6-1:*** *Thank you for the advice, according to which we have amended the manuscript so that it now uses the correct tenses. In addition, as mentioned in our previous response, the manuscript has also been proofread by native-English-speaking scientific editors.*

**Comment 6-2:** Please utilize the proper radiopharmaceuticals nomenclature, as indicated in the official EANM statement: <https://www.eanm.org/content-eanm/uploads/2019/12/EANM_GUIDANCE-_TRACER_NOMENCLATURE-1.pdf>

***Response to Comment 6-2:*** *We have changed the radiopharmaceuticals’ nomenclature, in accordance with the EANM statement, from At-211 MABG, I-123 MIBG, and I-131 MIBG to [^211^ At] MABG, [^123^I] MIBG, [^131^I] MIBG, respectively.*

**Comment 6-3:** Please adjust the definition of “multiple active cancers”, which could be confusing in its current form; I suggest “presence of malignancies other than the one object of the study or history of other malignancies with a disease-free interval <5 years”

***Response to Comment 6-3:*** *Thank you for your suggestion. We have now changed the sentence as follows: ““Multiple active cancers” refers to presence of malignancies other than the one included in the study or history of other malignancies with a disease-free interval <5 years.”*

**Comment 6-4:** Please explain which drugs fall in the category described in the second point of the exclusion criteria; the recommended withdrawal period for these medications and the one mentioned in point 3 should also be added.

***Response to Comment 6-4:*** *We have now provided this information in the manuscript as follows: “Patients who cannot stop the dosage of a drug that suppresses the accumulation of MABG according to the EANM procedure guidelines for ^131^I-meta-iodobenzylguanidine (^131^I-mIBG) therapy during the study period.” In addition, we have clarified the withdrawal periods of 2 and 3 point as "during the study period."*

**Comment 6-5:** Please expand on the “uncontrolled thyroid dysfunction” that could lead to an exclusion from the study.

***Response to Comment 6-5:*** *We have now changed “thyroid dysfunction” to “thyroid dysfunction including hypothyroidism and hyperthyroidism”.*

**Comment 6-6:** Discussion: consider changing the word “penetrability” to “shorter range in water”; the sentence “have a shorter half-life of At-211” referred to alpha-radiation is confusing and must be corrected or removed.

***Response to Comment 6-6:*** *Accordingly, we have changed the word “penetrability” to “shorter range in water”, and revised the sentence as follows:*

*“[^211^At] At is an α-emitting nuclide with short half-life, and because α-rays have shorter range in water than β-rays, there is no need to isolate patients treated with this drug for long periods in an isotope treatment room.”*
