# Supplementary material for: Evaluation of pharmacokinetics, safety, and efficacy of [211At] meta-astatobenzylguanidine ([211At] MABG) in patients with pheochromocytoma or paraganglioma (PPGL): A study protocol
Source: PLoS One. 2024 May 28;19(5):e0303623. doi: 10.1371/journal.pone.0303623 (PMC11132457; doi:10.1371/journal.pone.0303623)
Supplement: Attachment — Submitted filename: renamed_657e6.docx [file pone.0303623.s005.docx]

**Responses to Reviewer’s**

**Comment of Reviewer 2.**

Major revisions: Include a comprehensive statistical analysis plan. Include details for determining the MTD and RD. Also, state the statistical methods that will be used to analyze the secondary endpoints.

Minor revisions. 1-Identify the software that will be used to capture the data as well as the software that will be used for the statistical analysis. 2-To assist in the review process, add line numbering to the document.

***Response to all Comments of Reviewer 2:*** *Thanks for your valuable comments. We have added following sentences to the “Statistical analysis” section (pages 20 to 21):*

*“MTD is determined as the highest dose level at which none of the three cases developed DLT or only one of the six cases developed DLT. RD is determined as the same dose of MTD. All secondary endpoints are described as descriptive statistics, no formal statistical hypothesis test is performed.　Statistical analyses are performed with SAS software, version 9.4 (SAS Institute).”*

*“PK/PD analyses are performed with Phoenix WinNonlin software (version 8.1, Certara, Inc.).”*
